# Systematic review and quantitative and qualitative comparative analysis of interventions to address HIV-related stigma and discrimination

**DOI:** 10.1097/QAD.0000000000003628

**Published:** 2023-06-19

**Authors:** Laura Ferguson, Sofia Gruskin, Maria Bolshakova, Mary Rozelle, Sachi Yagyu, Kasoka Kasoka, Tessa Oraro-Lawrence, Aneesa Motala, Lucy Stackpool-Moore, Susanne Hempel

**Affiliations:** a Institute on Inequalities in Global Health, University of Southern California; b Southern California Evidence Review Center, Population and Public Health Sciences, University of Southern California, Los Angeles, California, USA; c International AIDS Society, Geneva, Switzerland; d Watipa, Sydney, Australia.

**Keywords:** discrimination, evaluation, HIV, implementation, stigma, systematic review

## Abstract

A strong global commitment exists to eliminate HIV-related stigma and discrimination, and multiple strategies to reduce or eliminate stigma and discrimination have been tried. Using a PICOTS framework and applying the Grading of Recommendations, Assessment, Development, and Evaluation (GRADE) criteria, we undertook a systematic review to determine the success of interventions aiming to address internalized stigma, stigma and discrimination in healthcare, and at the legal or policy level, and to identify their critical success factors. Random effects meta-analyses summarized results wherever possible. We carried out a component analysis to identify and characterize successful interventions. Internalized stigma interventions were diverse: across all studies, we found a reduction of stigma but it was not statistically significant [standardized mean difference (SMD) 0.56; confidence interval (CI) 0.31–1.02; 17 studies). For interventions to address stigma and discrimination in healthcare settings, effect estimates varied considerably but most studies showed positive effects (SMD 0.71; CI 0.60–0.84, 8 studies). Boosted regression analyses found that a combined approach comprising education, counseling, community participation, support person, and access to a HIV specialist often yielded success. Studies of efforts to address stigma and discrimination through law and policy documented, mostly qualitatively, the effect of court cases and directives. Across a range of settings and populations, promising interventions have been identified that, through diverse pathways, have positively impacted the types of stigma and discrimination studied. This evidence base must be built upon and brought to scale to help reach global HIV-related targets and, most importantly, improve the health and quality of life of people with HIV.

## Introduction

HIV-related stigma and discrimination constitute significant barriers to HIV responses around the world. Analyzing intervention experiences to date is useful for identifying lessons that can inform more effective and efficient interventions moving forward.

HIV-related stigma has been defined by the Joint United Nations Programme on HIV/AIDS (UNAIDS) as negative beliefs, feelings and attitudes towards people with HIV, groups associated with people with HIV (e.g. their families), and other key populations at higher risk of HIV infection, such as people who use drugs, people engaged in sex work, men who have sex with men, and transgender people [1]. Different domains have been identified in attempts to categorize HIV-related stigma, including internalized, anticipated, perceived, enacted, externalized and structural stigma [2]. The diversity of co-existing definitions of stigma has spawned a multitude of diverse interventions and a lack of consensus not only on key aspects of stigma but consequently how best to address it.

HIV-related discrimination is legally grounded and most often defined as any distinction, exclusion or restriction based indirectly or directly on a person's real or perceived HIV status [3]. Despite this distinct definition, in many interventions, discrimination is used to mean many different things or is not differentiated from stigma. Working definitions adopted for this review are described in the Appendix.

There is strong commitment to eliminate HIV-related stigma and discrimination, starting with global political commitments and reflected in global and national strategies as well as in the work of the many organizations and collaborations working to address them [4]. Yet, learning across interventions designed to mitigate against the experience and harmful impacts of stigma can be hindered, in part, by the diversity of issues to be addressed [5].

Experiences of stigma and discrimination for people living with and most affected by HIV can occur at many levels. This review systematically identifies and assesses interventions to address HIV-related internalized stigma; stigma and discrimination within healthcare settings; and in laws and policies. These focus areas were selected as each requires a very different type of response, suggesting that, even as one might expect strong similarities within each domain, there might be substantial heterogeneity in interventions across these areas. Recognizing that to reduce stigma at scale, synergistic attention is required across all three domains, this is the first systematic review to look across interventions that address each of these areas.

### Review questions

The systematic review was guided by two key questions:

1. Which interventions have been evaluated that aimed to reduce these types of stigma and discrimination or mitigate their adverse effects and what are the effectiveness and unintended consequences of the interventions?
2. What common ‘critical factors for success’ can be identified across the interventions that have been evaluated that might inform future interventions?

## Methods and analysis

The systematic review followed a detailed protocol, is registered in PROSPERO (CRD42021249348), and the reporting follows the PRISMA guidelines [6–8]. It followed a transparent and rigorous procedure to minimize review selection and reporting bias. We searched multiple disciplinary and interdisciplinary sources to capture all relevant evaluations. Citations and full text publications were screened by independent reviewers to reduce reviewer errors and bias. Eligibility decisions, including reasons for exclusion, were tracked in citation management software. Data abstraction and critical appraisal were conducted in online software designed for systematic reviews using detailed, pilot-tested forms with clear reviewer instructions to avoid ambiguity and ensure replicability of coding decisions. The collected data are accessible in a review data repository [9].

The systematic review is focused on people living with or most affected by HIV, including gay men and other men who have sex with men, sex workers, transgender people, people who inject drugs and prisoners and other incarcerated people [10].

### Search strategy

To identify primary research studies, we searched PsycINFO, primarily to identify psychological and social research on stigma; searched the health-specific research database PubMed; and used the general scientific research database Web of Science primarily to identify studies on stigma and discrimination in laws and policies. For the latter, we also identified government and nongovernmental organization reports indexed in the Universal Human Rights Index, HeinOnline, PAIS Index and HIV Legal Network.

Additional grey literature searches targeted the websites of the IAS – the International AIDS Society, UNAIDS, United Nations Development Programme (UNDP), STRIVE, Health Policy Plus, and Sage. Search strategies are in the Supplementary Appendix.

Systematic reviews were instrumental for reference-mining to ensure that all relevant materials had been considered. Systematic reviews were identified through PubMed using the systematic review filter, through PsycINFO and Web of Science, as well as through the Cochrane Database of Systematic Reviews and the Campbell Collaboration. Furthermore, we searched the review registries PROSPERO and Open Science Framework to ensure that all relevant registered systematic reviews had been identified.

### Eligibility criteria

We used a PICOTS (participant, independent variable, comparator or study design, outcome/measure, timing, and setting) framework to structure the eligibility criteria. Detailed eligibility criteria are documented in the Supplementary Appendix. Briefly, publications documenting interventions addressing people living with or perceived to be living with HIV and people from groups disproportionately affected by HIV were eligible. Eligible studies had to evaluate strategies and policies aimed at preventing, reducing or mitigating HIV-related stigma and discrimination. Interventions had to address HIV-related internalized stigma, stigma and discrimination in healthcare or in laws and policies. Studies of *internalized stigma interventions* had to report on a concurrent or historic comparator [e.g. randomized controlled trial (RCT)] to be eligible. Studies of *healthcare interventions* were restricted to RCTs and controlled trials, as well as large observational studies targeting healthcare delivery organizations. For studies relating to *law or policy*, no comparator was required if the study demonstrated an alternative analysis to determine the effect of the law/policy. Intervention studies had to provide a structured evaluation of the intervention and report the effects on an indicator of stigma or discrimination to be eligible. Publications from 2008 on were included, the year in which the first People Living with HIV Stigma Index was published [11]. Searches were completed on 6 May 2021. The review was not restricted by setting but was restricted to English language.

### Data abstraction and critical appraisal

We documented the study identification details, year of publication, country, study design, sample size, participant details, context/setting; intervention type, intervention description, and intervention components, comparator type and comparator description, definition and measures of stigma and discrimination, and findings for the outcome measures. Where additional types of stigma or discrimination might be relevant to the study population, these are noted.

We abstracted data for the main effectiveness signal and adverse events or unintended consequences. Effect estimates were based on the results in the intervention group relative to a control group at the longest follow-up reported. In the absence of a concurrent control group, the change compared with the preintervention status was used to determine intervention effects. We computed measure-independent effect estimates, that is, standardized mean differences (SMD) for continuous outcomes and relative risks for categorical outcomes, to facilitate comparisons across studies.

Intervention evaluations were reviewed for potential selection, detection, performance, attrition, reporting and study-specific sources of bias, adapting RoB 2 and ROBINS-I criteria [12,13]. Data were abstracted and appraised by one reviewer and checked for accuracy by a methodologist and a content expert.

### Component analysis

To determine what characterized successful interventions, we first broadly categorized the type of intervention.

### Intervention type

The categorization was undertaken based on the abstracted intervention description and was blind to the study findings. The categories were used to form subgroups of more homogeneous intervention types.

We reviewed published systematic reviews on internalized stigma [14,15], stigma and discrimination in healthcare settings [2,16–25], and law and policy to identify categorization systems for each of these types of interventions. We adopted established categorization systems for interventions to address internalized stigma [15] and stigma and discrimination in healthcare settings [17]. We did not identify a categorization system for legal and policy approaches, requiring us to create our own. The systems used are described in the Supplementary Appendix.

### Intervention components

Next, we derived a set of common intervention components as most identified internalized and healthcare interventions were complex, multicomponent approaches. The components were drawn from the identified literature and informed by existing intervention frameworks [2,15]. The presence or absence of components was documented and tabulated for each successful and unsuccessful intervention (as determined statistically by the main relevant outcomes), together with the focus of the intervention (reduction of internalized stigma or stigma and discrimination in healthcare). Only internalized stigma and healthcare interventions formed part of this analysis as the reporting for the legal analyses mostly described a categorical change rather than a multicomponent intervention. The differentiated components and the rationale for establishing each category are in the Supplementary Appendix.

We investigated the effect of individual components as well as component combinations using quantitative and qualitative comparative analyses (QCA). Effects of individual components were investigated in quantitative meta-regressions by adding the variable to the meta-analysis model to determine whether effect estimates were associated with the variable. We applied boosted regression to determine the relative importance of components in multicomponent intervention studies.

Effects of component combination on intervention success were evaluated qualitatively. We excluded from the analysis studies not reporting on: stigma or discrimination; the effect relative to a control group where a control group was present; or statistical significance where insufficient information was provided to calculate the effect. We established a Truth Table to identify conditions (intervention component combinations) that can produce intervention success (see Supplementary Appendix) [26,27].

### Summary of findings and body of evidence assessment

We adapted the GRADE (Grading of Recommendations, Assessment, Development, and Evaluation) criteria study limitation, inconsistency, imprecision, indirectness, reporting bias to upgrade and the criteria large effect, dose–response relationship, and confounding would mask an effect, to assess the quality of evidence [28]. This assessment of the body of evidence was used to arrive at internationally accepted certainty categories that communicate our confidence in the findings. We reviewed the appropriateness of the starting point of low quality of evidence for nonrandomized studies before upgrading or downgrading the evidence to avoid floor effects and ensure meaningful differentiation.

## Results

**Fig. 1:**
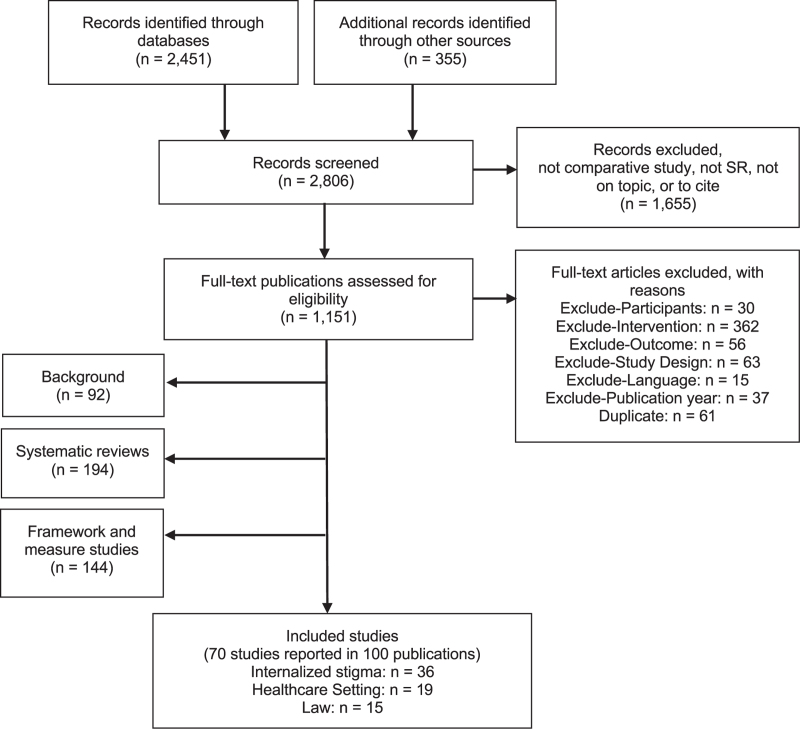
Literature flow.

The evidence review identified 70 intervention evaluations [29–98]. The literature flow is documented below (Fig. 1).

The boxes in Fig. 1 are not mutually exclusive, meaning that the numbers do not sum cleanly.

Studies included evaluate interventions to reduce internalized stigma, interventions to reduce stigma and discrimination in healthcare, and changes in law and policy. Three evidence tables in the Supplementary Appendix show the identified research.

**Fig. 2:**
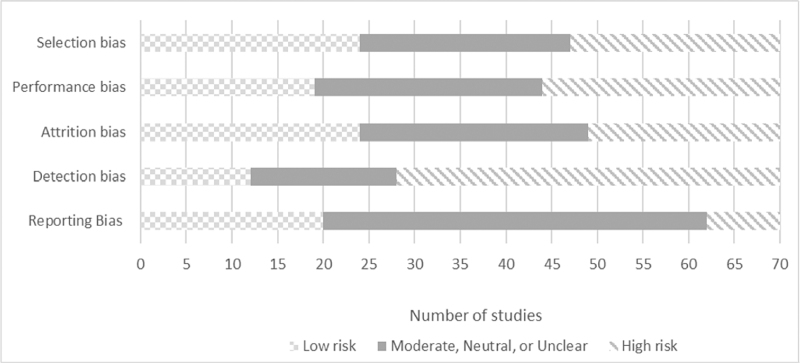
Risk-of-bias assessment for the evaluations.

Figure 2 shows the risk-of-bias assessment for the evaluations.

Studies varied widely in their potential for bias; risk of bias for each individual study is shown in the Supplementary Appendix. We classified 23 of 70 studies as high risk of selection bias. Reasons included the use of nonrandomized studies with convenience samples, often without control group, or baseline differences in controlled studies. Twenty-six were classified as high risk of performance bias with participants and study personnel aware of the intervention allocation, potentially affecting participants’ experiences. Twenty-one studies reported a substantial dropout rate and were classified as high risk for attrition bias. A large number of studies used self-reported measures in combination with lack of blinding to the intervention or control allocation, suggesting high risk of detection bias. We classified 62 studies as either low risk of bias or unclear for reporting bias, using stigma as the primary outcome. No studies were classified as low risk across all five types of bias assessed. However, four studies were classified as low-risk for four of these five types [31,48,53,56].

### Internalized stigma interventions

We identified a substantial number of interventions either explicitly targeting internalized stigma or evaluating the effect of a broader stigma intervention on internalized stigma. In the Supplementary Appendix, the evidence table provides a detailed overview and the tree diagram documents the studies’ geographic dispersion. Table 1 provides an overview of the identified studies that evaluated interventions with the primary objective to reduce internalized stigma.

**Table 1:** Interventions with primary objective to reduce internalized stigma.

First author, year Country Study design Sample size	Participant details Cooccurring stigmas Context/setting Study objective	Intervention Duration Comparator Length of follow-up	Stigma results Unintended consequences
Bauermeister, 2019 [33] Hightow-Weidman, 2017 [99] **Country:** USA **Study design:** Prepost **Sample size:***N* = 238	**Participant details:** Identify as male sex assigned at birth, be between 18 and 30 years old (inclusive), identify as African American/Black, reside in North Carolina, and have access to a mobile device with internet connectivity who were HIV-negative, HIV-positive, or HIV status unknown **Cooccurring stigmas:** sexual orientation **Context/setting:** other: mobile/internet **Study objective:** to seek to characterize whether sexuality related and HIV-related stigma scores change over time	**Intervention** Intervention arm participants had access to three spaces where they could share and receive information and experiences and garner social support; the Forum was a space where participants could initiate or contribute to conversations within various topic areas (e.g. getting tested, safer sex, dating & relationships, healthy living, fashion & entertainment, life skills, current events); in the Ask Dr W section of the site, participants could post anonymous questions to a board-certified infectious disease doctor; the question and response were then posted (within 48 h) for all participants to view; in the Getting Real section, participants could share and comment on multimedia content that they created themselves (e.g. poetry, videos, images, reflections) or that they linked to on the web (e.g. news stories, YouTube videos) **Duration (months):** 3 months **Comparator** **Length of follow-up:** 6 months	4.6% made internalized HIV stigma contributions to the forum. Steward's 10-item subscale showed a reduction in perceived HIV stigma (*P* ≤ 0.05); forum posts indicating anticipated HIV stigma reported increases in HIV stigma over time (*P* ≤ 0.01). **Unintended consequences:** NA
France, 2019 [40] **Country:** Zimbabwe **Study design:** Prepost **Sample size:***N* = 23	**Participant details:** people living with HIV were recruited from a local HIV support network via open call for volunteers; all were members of Harare-based support groups which were part of the ZNNP+ network **Cooccurring stigmas:** NA **Context/setting:** Community **Study objective:** to assess the impact of peer support for people living with HIV	**Intervention** A facilitated programme incorporating Inquiry-Based Stress Reduction: the Work of Byron Katie, which is a guided form of self-inquiry, which helps users to overcome negative thoughts and beliefs designed to support participants to question and work through self-stigmatizing beliefs; technical support and curriculum development was carried out by International Certified Facilitators, coordinated by The Work for Change organization; participants took part in 12 4 h group sessions run over 12 weeks, a weekly 1 h individual session with a facilitator, as well as homework **Duration (months):** 3 months **Comparator** **Length of follow-up:** 3 months	Participants reported significant improvements in Internalized AIDS Stigma Scale 3-month follow-up vs. baseline (median difference 0.5, IQR 0, 0.8; *P* = 0.003). **Unintended consequences:** NA
Go, 2015 [42] **Country:** Vietnam **Study design:** RCT **Sample size:***N* = 455	**Participant details:** HIV-positive male adults, able and willing to bring in an injecting network member for screening, had sex in the past 6 months, and injected drugs in the past 6 months **Cooccurring stigmas:** injection drug use stigma **Context/setting:** community **Study objective:** to develop and evaluate a behavioral intervention for people who inject drugs	**Intervention** Structural-level community stigma reduction program combined with individual-level counseling and skill-building support groups; the intervention draws on theories of social action, social identify, and diffusion of innovation; social action theory, which centers on the interaction between internal affective states (e.g. internalized stigma and avoidance coping), the social environmental context (e.g. peer networks, sexual partners, community) and self-regulation capabilities (e.g. mastery of technical, social and problem solving skills), guided intervention content **Duration (months):** Individual-level intervention arm received two individual counseling sessions, two small groups, and an optional dyad session; structural level intervention received a two-part video series and six HIV education sessions **Comparator** Standard-of-care HIV testing and counseling **Length of follow-up:** 24 months	Average HIV stigma scores across 22 items covering internalized, perceived, and experienced stigma remained stable over the follow-up and comparable across arms. **Unintended consequences:** NA
Nyamathi, 2013 [61] **Country:** India **Study design:** RCT **Sample size:***N* = 68 2 villages	**Participant details:** rural women, live with HIV, be between the ages of 18–45, be on antiretroviral therapy for at least 3 months, have a CD4^+^ cell count of over 100 **Cooccurring stigmas:** NA **Context/setting:** community **Study objective:** To assess the impact of an intervention to reduce internalized stigma and avoidant coping in a sample of rural women living with HIV in India	**Intervention** Six sessions included the following topics: HIV and dealing with the illness; learning about antiretroviral therapy and ways to overcome barriers; parenting and maintaining a healthy home environment; how to improve coping, reduce stigma and care for family members; basics of good nutrition and easy cooking tips; and benefits of engagement in a life skills class, such as computer skills, marketing, and embroidery; the primary role of the intervention Asha was to visit the four to five women living with HIV assigned to them weekly for 15–60 min, monitor barriers to ART adherence, and provide assistance to mitigate any barriers they faced in accessing healthcare or the prescribed treatment; assistance included accompanying to the district hospital or psychologist, and counseling them about coping strategies to deal with side effects, such as discrimination; the AL Asha were trained to inquire about side effects, provide basic education and counseling, promote healthy life style choices, and link women living with HIV with community resources to match health needs **Duration (months):** six sessions lasting 45 min each **Comparator** The Usual Care participants received matched sessions in terms of number and length of time and generally included topics 1–3, as described for the Asha-Life program, followed by three additional sessions, which were essentially question and answer type sessions, the women received standard education and some nutritional supplements **Length of follow-up:** 6 months	Participation in the intervention was associated with a significant reduction in internalized stigma (using the 10-item scale by Ekstrand (*P* = 0.001). **Unintended consequences:** NA
Petersen, 2014 [67] **Country:** South Africa **Study design:** RCT **Sample size:***N* = 76	**Participant details:** attending dedicated antiretroviral clinic for treatment, 18 years or older, not requiring urgent medical attention, no difficulty with hearing, speaking or cognition **Cooccurring stigmas:** NA **Context/setting:** Community, other: Public clinic in KwaZuluNatal province **Study objective:** To assess the feasibility of a group-based counselling intervention for depressed HIV-positive clients in primary healthcare in South Africa using a task shifting approach	**Intervention** Group-based Interpersonal Therapy intervention consisted of eight sessions; each session comprised a number of steps starting with introducing a common trigger or exacerbating factor using a vignette; second step involved asking participants who identify with the story to share their problem; third step drew on problem management to address the triggers of depression and cognitive behavioral techniques for exacerbating factors (internalized stigma also addressed), promoting healthy thinking in the case of negative intrusive thoughts and behavioral activation for social isolation; fourth step involved getting participants to identify problems that they were going to work on in the next week; intervention was delivered by two of the lay HIV counsellors from the clinic who were trained in the intervention **Duration (months):** 2 months **Comparator** Received normal standard of care, which included the counseling services provided by the HIV counsellors **Length of follow-up:** 3 months	The facilitators identified the session on internalized stigma as most helpful as it helped to change the way the participants thought about themselves, which then helped them to withdraw less and have more hope. **Unintended consequences:** NA
Prinsloo, 2017 [68] Prinsloo, 2016 [100] **Country:** South Africa **Study design:** Prepost **Sample size:***N* = 632 62 people living with HIV, 570 community members, live within the same municipal ward	**Participant details:** people living with HIV from two Department of Health clinics in NW South Africa; community members selected by random voluntary sampling from 780 households **Cooccurring stigmas:** received stigma^a^ **Context/setting:** healthcare, community **Study objective:** to explore, describe and determine whether an HIV stigma reduction community ‘hub’ intervention would change the HIV stigma experiences of people with HIV	**Intervention** Two HIV stigma-reduction community hubs in the ward; each hub had a team comprising two mobilisers, one person with HIV, and one noninfected person who had a close relationship with a person with HIV; mobilizers lived in the ward and were involved in a previous HIV stigma-reduction study involving understanding and coping with HIV stigma, and the planning/implementation of their own HIV stigma-reduction community project, they underwent a 4-day workshop to become community mobilizers and were trained to present workshops on ‘Understanding HIV stigma’, ‘Coping with stigma’, how to lead a support group, and also in effective record keeping of community activities during the intervention; 27 3 h workshops on ‘Understanding HIV stigma’ for both people with HIV and community member groups; 5 workshops on ‘Coping with HIV Stigma’ for those interested in continuing; and, weekly door-to-door ‘Understanding HIV stigma’ teaching with a pamphlet; after the workshops a six-session support group was run for both the community and groups of people with HIV; mobilisers presented eight psychodrama group performances on the theme of ‘HIV stigma reduction’ (e.g. churches, gatherings and clinics); further mobilizer activities included a HIV stigma-reduction community project with home visits, support and education at clinics, two stigma campaigns at taxi ranks and in Main street **Duration (months):** 5 months **Comparator** **Length of follow-up:** 5 months	The HIV/AIDS Stigma Instrument – people with HIV (HASI-P, includes an internal stigma scale to assess negative self-perception) found a mean score of 36.92 before the intervention and 34.35 after the intervention, which showed no statistically (*P* = 0.09) but a small practically (*d* = 0.25) significant difference that indicate a small reduction in stigma experienced by people with HIV after the intervention. The negative self-perception subscale found no statistically (*P* = 0.14) but a small practically (*d* = 0.21) significant difference between the mean score of 6.3 before the intervention and 5.6 afterwards. **Unintended consequences:** PWH re-lived traumatizing events when they described instances of verbal abuse. about their health condition, community members freely gossiped and spread rumors like, ‘Hey did you hear? Your neighbour is sick’. Being labelled on physical appearance was common and even being mocked for physical inabilities like having support when walking; community came up with new names for people living with HIV (’hemela’, referring to dying and going to heaven)
Rao, 2012 [70] **Country:** USA (Seattle) **Study design:** Prepost **Sample size:***N* = 24	**Participant details:** recruited from a publicly funded HIV clinic through the use of advertisements in the form of flyers and promotion from the HIV clinic nurses **Cooccurring stigmas:** NA **Context/setting:** healthcare **Study objective:** to feasibility test and gather preliminary data on the effectiveness of the intervention to reduce symptoms of internalized stigma for African American women living with HIV	**Intervention** Participants worked in break-out sessions, which included discussions (to help participants develop new coping skills for stigma) and video presentations; discussions were encouraged after viewing the videos; afterwards, a structured discussion of key concepts followed; participants also conducted an exercise in which they tossed a yarned ball to each other and exclaimed ‘positive names for the web that had been created and [that had] linked them together **Duration (months):** two consecutive afternoons with a 4–5 h workshop **Comparator** Preintervention data **Length of follow-up:** 0.25 months	Stigma Scale for Chronic Illness showed decreased internalized stigma from the start of the workshop from a mean of 38.0 (SD 11.4) to 34.2 (SD 11.7) 1 week after (*P* = 0.07). **Unintended consequences:** NA
Rao, 2018 [71] Fabian, 2020 [101] **Country:** USA (Chicago) **Study design:** RCT NCT01893112 **Sample size:***N* = 239	**Participant details:** Women who self-identified as having an African American racial/ethnic background, were 18 years of age or older, and had documentation of living with HIV **Cooccurring stigmas:** race/ethnicity **Context/setting:** Community **Study objective:** to assess the effect of a peer support workshop on HIV-related stigma among African American women living with HIV	**Intervention** The UNITY workshops (described as internalized stigma reduction intervention in prior studies) began with discussions of group expectations and what the term ‘stigma’ meant to them; after watching a ‘trigger’ video, the facilitator led a guided discussion; participants explored reactions to the video and personal experiences with stigma in a large group and in dyads; the next exercise involved brainstorming coping methods that group members have used to deal with stigma; in a segment about self-soothing, the facilitator led a guided visualization; a subsequent segment included modeling and practicing assertiveness (vs. passivity or aggression) in response to stigma; a self-esteem exercise and social support exercise finished out the first day; day 2 focused on disclosure with case studies and role play; the final exercise of UNITY engaged the group regarding how to ‘live positively’ with HIV **Duration (months):** 2 days **Comparator** A breast cancer education workshop was designed to match the UNITY workshop in terms of time and attention, and as such, was also held across two 4 h sessions using similar group formats; the facilitator for this arm was one research coordinator per site; no peer was involved **Length of follow-up:** 12 months	Both arms experienced decreases in mean stigma scores (Stigma Scale for Chronic Illness measuring internalized and enacted stigma) over time; GEE analysis was used and reduction in stigma was not statistically significant between the intervention groups (*P* = 0.7308). **Unintended consequences:** NA
Singh, 2020 [76] **Country:** India **Study design:** RCT NCT03746457 **Sample size:***N* = 752	**Participant details:** men who consumed at least one alcoholic beverage in the last 30 days, over 18 years of age, on antiretroviral therapy for at least 6 months **Cooccurring stigmas:** alcohol use **Context/setting:** Other: Government antiretroviral therapy centers **Study objective:** to identify the optimal sequence of the three interventions individual counseling, group intervention, and collective advocacy	**Intervention** The three interventions were individual counseling, group intervention, and collective advocacy; individual counseling and group intervention were based on formative research with the study population, the existing government and nongovernment organization guidelines, and infrastructure for individual counseling at antiretroviral therapy centers; group counseling at Community Support Centers included an alcohol risk-reduction module and a significant component to address stigma (sources of internalized, external and relational stigma including disclosure); the third intervention was based on the successful HIV advocacy efforts of the Network of Maharashtra Positive People, a collaborating project partner, and prior research in India by the principal investigators on community level advocacy and structural change and literature on the role of activism in reducing HIV-related stigma globally **Duration (months):** 27 months **Comparator** Received routine care approved by National AIDS Control Organization; the protocol called for people living with HIV at each visit to see the antiretroviral therapy counsellor who reviews antiretroviral therapy adherence, positive prevention and healthy living **Length of follow-up:** 27 months	There was a significant decrease in stigma from baseline measured by Berger's HIV stigma scale (*P* < 0.001). The greatest reduction was seen in the group that received interventions in the order collective advocacy, individual counseling, group intervention. **Unintended consequences:** NA
Tshabalala, 2011 [85] **Country:** South Africa **Study design:** Prepost **Sample size:***N* = 20	**Participant details:** HIV-positive African women who were receiving treatment from the clinic and who experienced difficulties in dealing with stigma, who have lived with HIV for at least 3 months have passed Grade 10, and came from a poor to average socioeconomic background **Cooccurring stigmas:** enacted **Context/setting:** community **Study objective:** to develop a therapeutic intervention to assist women to change their conception of HIV and their sense of self-worth	**Intervention** A cognitive behavioral therapy model consisting of eight individual sessions to address the five commonly identified themes that underlie the negative experiences of women with HIV; after each session, homework was assigned to facilitate change from one session to the next; the themes include feelings of powerlessness, feelings of guilt and anger about the past, destructive behavior patterns, experience of the reaction of others, and uncertainty about the future **Duration (months):** 2 months, 8 sessions **Comparator** Control group were placed on a waiting list and received psychotherapy upon completion of the study. **Length of follow-up:** 2 months	Statistically significant decrease (intervention – 15.1 SD 7.7 vs. control –6.1, SD 7.1) in internalized stigma (Serithi Internalised Stigma Scale; *P* < 0.05). **Unintended consequences:** NA
Watt, 2020 [95] **Country:** Tanzania **Study design:** RCT NCT03600142 **Sample size:***N* = 1543	**Participant details:** women who were pregnant, at least 18 years old, able to understand Swahili, were attending the first antenatal care appointment at one of the two government health centers, male partner accompanying the enrolled women were able to enroll **Cooccurring stigmas:** anticipated stigma **Context/setting:** Healthcare **Study objective:** the Maisha intervention aimed to address HIV stigma to improve early care engagement and to reduce HIV stigmatizing attitudes	**Intervention** Delivered by the facilitator to either the individual woman or the woman and her partner; sessions were conducted in a private research room; session 1 included a video and brief counseling session (depicting a couple who test positive for HIV during a first antenatal care visit, and follows them as they learn to accept their status, navigate disclosing their status to her mother-in-law, and commit to taking daily therapy); counseling provides psycho-education on various components of HIV stigma, leads participants to reflect on HIV stigmatizing attitudes, and encourages empathy and inclusion toward people living with HIV in the community; session 2 was delivered immediately following the first antenatal appointment to all women identified living with HIV, along with their male partners, if enrolled, returning to content to discuss how issues of stigma might relate to the couple's situation and to reduce barriers of HIV care; session 3 asked women living with HIV to come alone to discuss relationship concerns, Third Wave cognitive behavioral concepts are introduced in this session, the facilitator emphasizes that one's feelings (internalized stigma) and thoughts (anticipated stigma) are connected. Finally, the counselor and participants consolidate the components of Maisha into an individualized action plan, which includes goals for HIV care engagement and antiretroviral therapy adherence, strengthening social support and considering selective HIV disclosures **Duration (months):** 0.75 months **Comparator** Received the standard of care HIV testing and counseling protocol in the clinic administered by clinic nurses, education about HIV and HIV testing; if an individual tests positive, counseling is initiated and women living with HIV should be registered for prevention of mother-to-child transmission care and immediately initiated on antiretroviral therapy, which is provided during the clinic appointment. **Length of follow-up:** 3 months	Participants in the intervention condition had greater reductions in internalized stigma measured by Scale A of the HIV and Abuse Related Shame Inventory (HARSI) (*β* = − 3.5; 95% CI −9.4 to 2.4) and lesser reductions in anticipated stigma than the control group (*β* = 2.0; 95% CI − 3.3 to 7.3). **Unintended consequences:** NA

Some of the terminology used by authors has been amended in favor of person-first language.

a Received stigma is defined by the authors as ‘how people act towards people with HIV and include all types of stigmatizing behaviour towards people with HIV, as experienced or described by themselves or others’.

Six of these explicitly targeted intervention evaluations were RCTs, while the other five were before–after study designs. All five of the evaluations from sub-Saharan Africa were interventions with adults living with HIV, with two focusing on women. One also included women's male partner and one included randomly selected community members. All three of the interventions carried out in the United States were with African American populations. The two interventions that included attention to co-occurring stigmas – one relating to drug use, one relating to alcohol consumption – were carried out among men living with HIV in Asia.

**Fig. 3:**
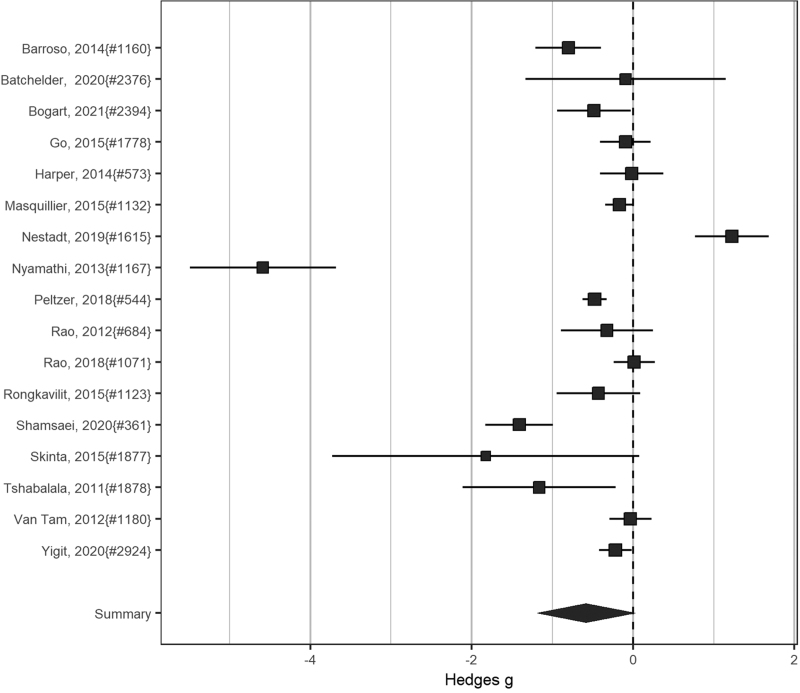
Intervention effects: internalized HIV-related stigma.

Figure 3 shows the effect of the intervention as standardized mean difference for all studies that reported sufficient detail to allow effect size calculations.

Across all studies, we found a reduction of stigma but it missed statistical significance (SMD 0.56; CI 0.31–1.02; 17 studies) and heterogeneity was substantial (*I*^2^ 98%). The intervention foci and study designs varied, and studies reported on different stigma outcomes/measures. Restricting to RCTs in a sensitivity analysis, we found similar estimates (SMD 0.57; CI 0.24–1.33; 12 RCTs; *I*^2^ 99%). Excluding two outliers, a small before–after study reporting substantial changes and an RCT with baseline imbalance, reduced heterogeneity (*I*^2^ 84%) and the effect of the stigma interventions was statistically significant (SMD 0.68; CI 0.53–0.87; 15 studies).

### Interventions to reduce stigma and discrimination in healthcare settings

Studies evaluated complex interventions (see Table 2). Due to the diversity of outcome measures, information is only presented on the study design and intervention description; full description of results and studies’ geographic distribution are in the evidence table in the Supplementary Appendix.

**Table 2:** Interventions to reduce stigma and discrimination in healthcare settings.

First author, year country, study design, sample size study population	Intervention description
Arora, 2014 [30] India, RCT, *N* = 65 Follow-up: 0.1 months Female student nurses studying in third year of BSc nursing and General nursing	A 5-day empowering program was prepared in consultation with eight experts from the community medicine and nursing field to expand the understanding of student nurses and modify their beliefs related to HIV and AIDS. Content focused on the magnitude, basic dynamics, mode of transmission and prevention of HIV, and altering the beliefs of student nurses about HIV and AIDS; lecture, group discussion, role play and case-based scenarios were used in the role plays to bring about a change in the thought process of students
Kaponda, 2009 [46] Malawi, Prepost, *N* = 855 Follow-up: 10 months Malawian urban clinical and nonclinical hospital workers	Sessions were implemented by trained peer-group facilitators; the Mzake ndi Mzake intervention for community hospital workers consisted of six sessions focused on the HIV epidemic and stigmatization; human sexuality and sexually transmitted infections; HIV prevention, AIDS, and testing; partner negotiation; condom use; and how to contribute to community HIV prevention; four new sessions were added for health workers only on HIV issues they reported that they frequently encountered: a nontechnical overview of HIV treatment and symptom management, universal precautions, helping individuals and families address HIV prevention, and ethical issues for health workers related to HIV. Every session included guided discussions, role plays, return demonstrations with corrective feedback, and an assignment to practice a specific skill before the next session
Li, 2013 [48] China, RCT, *N* = 1760 Follow-up: 12 months Health providers (aged 18 or older) who had regular contact with patients, including doctors, nurses, and lab technicians	Popular opinion leaders attended four group sessions over a 1-month period and three reunion sessions; sessions covered complying with universal precaution procedures and ensuring occupational safety, fighting against stigma and improving the provider – patient relationship, taking actions and making efforts to care for patients, and overcoming difficulties and building up a better medical environment; trained popular opinion leaders providers were inspired to serve as behavior change endorsers and disseminate intervention messages to their coworkers; interactive techniques were used to effectively deliver the messages to other providers in the hospital
Lohiniva, 2016 [50] Egypt, Controlled trial, *N* = 471 Follow-up: 3 months All physicians and nurses in the surgical units in the selected hospitals were invited to voluntarily join the study	Five interactive training modules including discussions and practical exercises: a module on HIV background and stigma, on medical ethics, on childbirth, and two modules addressing infection prevention and control measures, including standard precautions and aseptic techniques for invasive procedures
Mak, 2015 [53] China, RCT, *N* = 88 Follow-up: 1 month Healthcare professional students	The intervention focused on increasing sensitivity to the feelings of people with HIV through experiential games followed by group discussion; inspired by Cornett's empathic learning intervention, activities were designed with three goals in mind: to allow participants to gain first-hand experience of potentially stressful situations that people with HIV might encounter on a day-to-day basis; to encourage participants to experience the feelings and thoughts of people with HIV and to allow them to acknowledge their own sense of vulnerability and helpfulness in the face of similar stressors; and to provide a platform for participants to talk openly; immediately following a 30 min didactic session on HIV knowledge, the group participated in two different experiential games.
Mbela, 2011 [56] Malawi, RCT, *N* = 417 Follow-up: 30 months Intervention and control clinical and nonclinical district health workers at five rural health centers	The intervention addressed primary prevention of HIV infection for health workers in their personal lives and workplace; 10 sessions provided information and skills; the first 6 sessions focused on HIV transmission, stigmatization, safer sex and partner negotiation and were used for both health workers and community members; the remaining sessions addressed universal precautions and teaching clients about HIV; sessions included guided discussions, role playing, return demonstrations with corrective feedback, and skill-building assignments; two co-facilitators offered the intervention to mixed gender groups of 10–12 health workers; groups were divided into clinical and nonclinical workers except at very small clinics; project staff offered the initial peer groups but some health workers volunteered to be co-facilitators; volunteers received training in the peer group content, learning activities, and group facilitation skills with practice and corrective feedback
Mockiene, 2011 [58] Lithuania, RCT, *N* = 206 Follow-up: 0.1 months Lithuanian registered nurses working in three randomly selected hospitals among the nine largest hospitals in Lithuania, and in primary healthcare centers attached to those	Group 1: 2-day workshop and distribution of written material Group 2: distribution of only written materials
Norr, 2012 [60] Chile, RCT, *N* = 555 Follow-up: 3 months Participants were selected from two municipalities, Interested community clinic health workers from select intervention or control clinics in Chile; five non-STD health clinics in each municipality (total clinics = 10) supplied the participants (community clinic workers)	The intervention (Mano a Mano Para Trabajadores de Salud) is a professionally assisted peer group intervention with groups of 10–12 health workers attending eight sessions (90 min each) covering (a) the importance of community HIV prevention; (b) standard precautions in the healthcare setting; (c) HIV testing and treatment in Chile; (d) offering care that respects human dignity and confidentiality; (e) human sexuality, sexual transmission of HIV, and HIV transmission through drug use and blood; (f) partner communication and HIV prevention; (g) counseling about HIV infection; and (h) teaching HIV prevention to clients and families; each session incorporated active learning activities such as role-plays; separate groups were created for the professional/technical staff and the less educated paraprofessional/ancillary staff
Nyblade, 2018 [63] Tanzania, Prepost, *N* = 564 Follow-up: 7 months Facility staff and clients living with HIV (adults and youth)	Health Policy Plus project total facility approach to stigma and discrimination reduction at all stages of the treatment cascade in health facilities; the intervention promoted a sustainable response through capacity building, facility ownership, and youth engagement; champion teams were identified in each facility and empowered by facility management to work collaboratively to reduce stigma and discrimination; activities carried out by the teams included declaring and drawing attention to facilities’ commitment to stigma-free care via community TV and radio spots and promoting client engagement and accountability through the creation and display of codes of conduct, signboards, nametags, t-shirts, and reporting boxes
Nyblade, 2020 [62] Ghana, Controlled trial, *N* = 2308 Follow-up: 6 months Facility management and clinical and nonclinical health staff	Participatory data dissemination and review workshops with staff; in the workshops, staff identified stigma and discrimination challenges and generated potential solutions; targeting the whole facility beyond HIV services, the intervention approach included a 2-day participatory stigma-reduction training for all staff levels (clinical and nonclinical) with delivery by staff and clients from the facilities trained as stigma-reduction facilitators; trainings were delivered to all categories of staff, with a target of reaching 70% of the facility workforce; trainings were based on preexisting global training materials targeted towards actionable HIV stigma drivers and were adapted to the Ghanaian context through an in-country stake holder workshop; training coverage in individual facilities ranged from 61 to 97%; each facility also created an 8-to-10-member ‘champion team’, which was provided $5000 USD to develop facility-specific ancillary activities, including launch events, anti-stigma and discrimination banners and posters, additional staff trainings, printed codes of ethics, reporting mechanisms, and staff name tags to enable identification and reporting of stigma and discrimination
Odeny, 2013 [64] Kenya, Prepost, *N* = 343 Follow-up: 12 months 40 health facilities staffed by 200 healthcare workers (144 were nurses); recruited patients were ≥18-year-old, HIV-positive and already enrolled in HIV care	Co-location and sharing of services and resources for HIV care and primary care including clinic space, clinicians, lab work, and health education for patients; intensive training on commodity management and streamlined supply chain, staff training for HIV
Pulerwitz, 2015 [69] Vietnam, controlled trial, *N* = 795 Follow-up: 7 months Healthcare staff (clinical, administrative, and support)	The intervention included six key components: establishment of a hospital steering committee, staff training, hospital policy development, provision of material supplies to facilitate the practice of universal precautions, provision of educational materials, and monthly monitoring; staff from the other northern and southern hospitals received a half-day training on basic HIV information that included testimonials from people with HIV and a full day on Universal Precautions; in addition, an extra half-day training on social stigma; training sessions on HIV and stigma were co-facilitated by people with HIV
Siraprapasiri, 2020 [77] Thailand, Prepost, *N* = 7482 Follow-up: 12 months Health-facility staff and people living with HIV clients	Three building blocks were implemented: policy and its translation into a roadmap for action; measurement development and routinization to inform intervention design and track progress; and intervention development and implementation of a stigma and discrimination reduction package for health facilities, which included 10 training modules spanning 12 h and covering the actionable drivers of stigma and discrimination, human rights, a client panel, and action planning
Sommerland, 2020 [79] South Africa, RCT, *N* = 652 Follow-up: 24 months Professional healthcare workers (doctors, nurses, and allied health professionals such as social workers and physiotherapists); administrative and management staff; and support staff (e.g. porters, cleaners, security staff, and household staff such as kitchen and laundry workers)	The intervention to reduce HIV and tuberculosis stigma among healthcare workers consisted of a workshop for healthcare workers who would educate other healthcare workers on how to reduce stigma among colleagues, and a social marketing campaign to help reinforce and disseminate the single key antistigma message in the workplace: ‘Let's Stop Stigma – Be kind to yourself and others’; it was also translated into Afrikaans and Sesotho; in the social and behavior change communication intervention, participants were taught about different forms and effects of stigma and the related health rights and responsibilities in the workplace, and were equipped to start conversations about HIV and TB stigma in the workplace with co-workers underpinned through an exercise in confronting one's own beliefs, values, attitudes, and behaviors; they were also taught how to respond to stigmatizing situations in the workplace, received social marketing materials (e.g. branded wristbands and pens) to support their stigma-reduction communications; staff identified change agents; there was a visible social marketing campaign, including posters that the healthcare workers could point to, to spark conversations or highlight what they are saying; medical doctors who were not able to attend the general staff training were educated on stigma through presentations based on workshop materials
Srinivasan, 2021 [80] India, RCT, *N* = 3182 Follow-up: 12 months Nursing students from 28 nursing schools that included private, nonprofit, and government-run nursing schools in the state of Karnataka, India who were aged 18 years or older and willing to participate	The intervention comprised of two self-guided sessions administered on a computer tablet and a skill-based group session co-led by study staff and a person with HIV from the local network; there were four modules in each session, and a study member was available to guide the participants through the sessions and answer questions; the intervention used videos and interactive exercises to increase awareness of stigma in healthcare settings, improve HIV transmission knowledge, develop empathy for people with HIV, address casual transmission fears, and teach the correct use of standard precautions with all patients; the group session with people with HIV included a discussion of experiences with health providers, review of key lessons learned, role-playing exercises using common stigma situations encountered in hospital settings, and the session concluded with participants making stigma reduction commitments
Uys, 2009 [90] Lesotho, Malawi, South Africa, Swaziland, Tanzania, Prepost, *N* = 175 Follow-up: 1 month People living with HIV and nurses; the nurses were identified by the nurse managers of their facilities as being interested in or involved with HIV care, and people with HIV were identified by support groups active in the area	The intervention combined sharing information (e.g. information on impact of stigma), increasing contact with the affected group, and improving coping through empowerment, and consisted of bringing together a team of approximately 10 nurses and 10 people with HIV in each setting and facilitating a process in which they planned and implemented a stigma reduction intervention
Varas-Diaz, 2013 [92] Puerto Rico, RCT, *N* = 507 Follow-up: 12 months Second-year medical students recruited from the four largest medical schools in Puerto Rico	The SPACES workshops were facilitated by health professionals that had experience with HIV-related patients; sessions targeted the sources and functions of HIV stigma, issues that can increase the severity of consequences, and instrumental and symbolic stigmas that manifest for HIV and included HIV knowledge, role of negative emotions in interactions, and behavioral skills
Wu, 2008 [96] China, RCT, *N* = 138 Follow-up: 6 months Service providers consisting of doctors, nurses, and lab technicians from 4 county hospitals	After a small group discussion on discriminatory language, attitudes, and behaviors in a medical setting, participants engaged in two rounds of a role-play session on ‘Discrimination among us’, which emphasizes the prevalence of discrimination in society and how it can make everyone a potential victim; all facilitators participated in training prior to the intervention
Yiu, 2010 [98] China, RCT, N = 89 Follow-up: 1.5 months Nursing students enrolled in the bachelor's program in nursing in Hong Kong	A 50 min standardized lecture and a question-and-answer session plus in-vivo contact with people with HIV (50 min sharing session given by two men with HIV)

Five of these interventions were directed towards students, six towards health workers, four towards health facility staff, including both clinical and nonclinical staff, and four towards both health workers and their clients living with HIV. Some interventions involved only a workshop or training, while others also included activities, such as creating facility steering committees, service integration, awareness raising or availing accountability mechanisms. Interventions’ duration ranged from 1 h to 1 year, with follow-up periods from 3 days to 24 months. Sample sizes were between 65 and 7482, with four studies having more than 1000 participants.

**Fig. 4:**
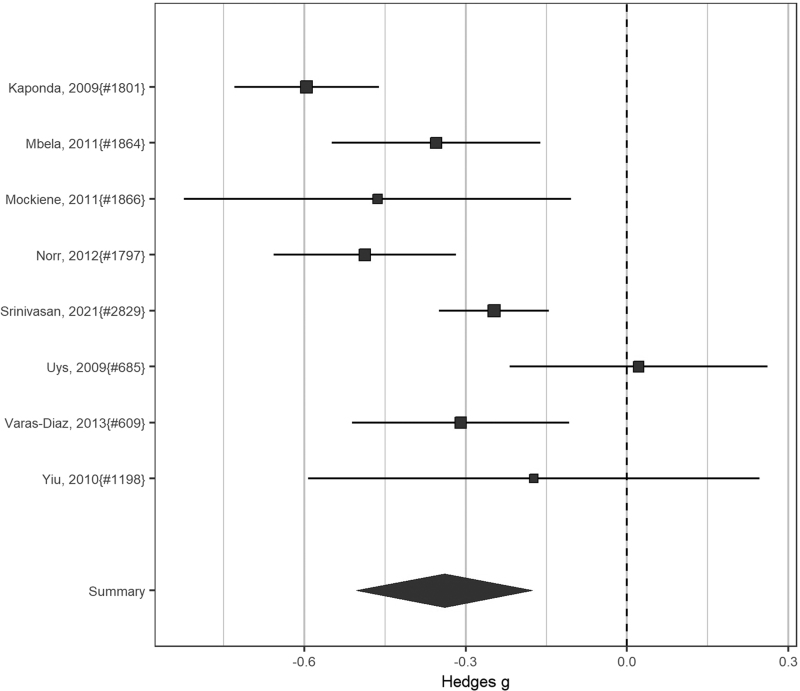
Intervention effects: HIV-related stigma and discrimination in healthcare settings.

Across studies with sufficient available data capturing stigma or discrimination, studies showed improvements (Fig. 4).

Effect estimates varied considerably but most studies showed positive effects (SMD 0.71; CI 0.60–0.84, 8 studies). Heterogeneity was considerable (*I*^2^ 76%). Restricting to RCTs, results remained statistically significant and heterogeneity was reduced (SMD 0.71; CI 0.61–0.83, 5 RCTs; *I*^2^ 43%).

### Interventions to reduce stigma and discrimination in law and policy

The evidence base on the effectiveness of interventions to reduce stigma and discrimination in law and policy is less robust than for the types of stigma studied above. Fewer studies were found through this review, and their objectives and outcomes were too diverse to analyze statistically, with more emphasis on qualitative documentation. The identified evaluations primarily documented the effects of court cases, directives, and legal empowerment strategies. Five studies reported on changes in the national legal and policy environment, four of which reported improvements, while one reported a negative change. Four court decisions were reported on with a focus on potential effects, all of which were decided in favor of people with HIV. Two studies reported on perceptions of HIV-related laws, two reported on advocacy efforts to improve the legal and policy environment, one reported on legal empowerment and promoting access to justice, and one provided a comparison of different policy options for HIV testing. Few studies documented the effects on stigma or discrimination specifically. The diverse set of studies is shown in detail in the Supplementary Appendix alongside their geographic locations.

### Summary of findings

Table 3 provides an overview of the evidence base from this systematic review across all three areas of stigma and discrimination reviewed. Based on the GRADE framework, it provides an overall assessment of the findings and the quality of the data for interventions addressing each type of stigma and discrimination.

**Table 3:** Summary of findings.

Intervention	Number of studies and RCTs	Reasons for upgrading or downgrading	Findings	GRADE
Interventions addressing internalized stigma	36 studies (20 RCTs)	Inconsistency (2)^a^	Reduction in internalized stigma but not statistically significant (SMD 0.56; CI 0.31–1.02; 17 studies)	Low
Interventions explicitly targeting internalized stigma	11 studies (6 RCTs)	Inconsistency, imprecision^b^	No systematic effect across studies (SMD 0.30; CI 0.03–3.26; 5 studies)	Low
Interventions to address stigma and discrimination in healthcare settings	19 studies (11 RCTs)	Indirectness^c^	Interventions reduce stigma and discrimination (SMD 0.71; CI 0.60–.84, 8 studies)	Moderate
Law and policy changes	15 evaluations	Study limitations, inconsistency, imprecision^d^	Studies describe effects of policy changes, court decisions, legal changes, legal empowerment, and discriminatory law or policy	Very low

The second row in this table is a subset of the studies in the first row.

a Downgraded because of heterogeneity and differences in direction of effects.

b Downgraded because of heterogeneity and wide confidence intervals.

c Downgraded because of diversity in assessed outcomes.

d Downgraded for study design (descriptive, no control group), diversity in evaluated law and policy changes, and lack of effect estimates.

### Component analysis

The distribution of the components across interventions and studies is presented in the Supplementary Appendix. As noted in the methodology, only interventions to address internalized stigma and stigma and discrimination in healthcare settings contribute to the component table.

Frequent intervention components found across internalized stigma and healthcare studies were *education* (34/55) and *counseling* (35/55).

Classifying the studies using existing intervention categorization systems [15,17] showed variety in their complexity, with a substantial proportion addressing a single level (e.g. an individual-level intervention). Dichotomizing the sample into interventions that were successful and those that were not, seven out of the 13 studies not reporting a statistically significant effect on stigma or discrimination addressed only the individual level. Of the 14 studies that included intervention at the structural level either alone or in combination with other components, 13 were successful. However, the effects of the ‘intervention’ and ‘structural’ categories were not statistically significant (*P* = 0.4246 and *P* = 0.9503, respectively).

We analyzed the effect of individual components as well as component combinations on intervention effectiveness. None of the individual components showed a systematic effect on the study effect size, indicating that according to these criteria, no single intervention component determined intervention success. Furthermore, the features *rights holders* (i.e. interventions targeting people in their capacity of having human rights) and *duty bearers* (i.e. interventions targeting people who are responsible for helping to ensure that the State fulfills its human rights obligations, such as health workers) were confounded with the context of the interventions (i.e. internalized stigma studies exclusively addressed rights holders). In addition, where *co-occurring stigma* is noted, this reflects a characteristic of the study population rather than an active intervention component, that is, interventions remained focused on addressing HIV-related stigma even if they were implemented with populations who may also experience stigma and discrimination based on other factors such as drug use. The number of intervention components included in individual studies ranged from two to eight. The total number of components was not associated with the effect estimate (*P* = 0.1906), that is, it cannot be assumed that interventions with more components will be more successful. We also tested whether the length of follow-up from baseline affected estimates but found no systematic effect (*P* = 0.4221).

Results of boosted regressions indicated that *community* participation (45.7), providing *education* (33.6), providing *counseling* (12.6), access to an *HIV specialist* (5.0), and engaging a *support person* (3.0) contributed to the success of interventions to address internalized stigma and stigma and discrimination in healthcare settings (relative information value in parentheses). However, this analysis should be interpreted with caution as it was not conducted within a meta-analytic framework, instead treating individual studies as cases with results dichotomized as either successful or not.

Finally, we assessed potentially winning combinations of intervention components. We assessed all possible combinations of the components: *community, education, counseling, HIV specialist*, and *support person.* Only 11 of 32 of all possible configurations have been evaluated in the literature to date. The analysis showed several paths to success (14 configurations with incl (consistency) ≥0.75) indicating equifinality. Combining all five components showed consistent success (all of the studies with this combination achieved a statistically significant effect on stigma or discrimination); however, this was only three studies. The minimization process identified the combination of *education, counseling,* and *support person* as one necessary condition to achieve intervention success consistently, but coverage of this specific configuration (incl 1, covS (raw coverage) 0.162) and the overall coverage across all component combinations was somewhat limited (covS 0.676).

Given the different approaches to address internalized stigma and stigma and discrimination in healthcare settings, we analyzed the datasets separately in sensitivity analyses. Based on conceptual considerations, we used the combination of *community* engagement, *counseling*, access to a *support person*, and involvement of a person openly *living with HIV* for the internalized stigma interventions dataset. However, we found no empirical study using all four components and the coverage of the model solution across all combinations was poor (covS 0.391). Adding the intervention component *education* increased the overall coverage of the model (covS 0.696) and identified the combination of *community*, *education*, *counselling*, and *support person* as a necessary condition (all four components need to be present to show success; incl 1, covS 0.130). In the dataset on healthcare settings, we considered the components *community*, *education*, and *total facility*. However, a minimization analysis indicated that a combination of *community* engagement and *education* as well as *education* and a *total facility approach* was sufficient to be associated with success (incls 0.90, covS 0.643). While *education* was a key component in these combinations, several studies showed lack of success when studies only used *education* without *community* engagement or *total facility approach* (7/17 studies, incl 0.714). The truth tables and model fit statistics are in the Supplementary Appendix.

### Unintended consequences

Across all of the studies reviewed, only six reported on unintended consequences – three relating to internalized stigma and three relating to stigma and discrimination in law and policy. In four cases, these unintended consequences were negative – increased experience of stigma because of the visibility of the intervention (*n* = 2), difficulty re-living traumatizing events that participants were asked to describe (*n* = 1) and reduced adherence to ART (*n* = 1) [32,55,57,68]. Two of the interventions to address HIV-related stigma and law and policy reported positive unintended consequences – one study found that a law criminalizing HIV exposure was not, as feared, an inadvertent deterrent on seropositive status disclosure, and legal advocacy to strengthen legal environments usefully started difficult conversations around adolescent sexual and reproductive health and abortion [41,88].

## Discussion

This review documents the evidence base for interventions addressing HIV-related internalized stigma, stigma and discrimination in healthcare settings, and in law and policy. Our detailed exploration of intervention components aims to disaggregate the different aspects of evaluated interventions to ascertain which (combination of) approaches can most effectively enable the prevention, reduction or mitigation of HIV-related internalized stigma and stigma and discrimination in healthcare settings.

Across, and even within, the interventions to address the reviewed types of stigma and discrimination, diversity of interventions and outcome measures make comparisons difficult, with very high levels of heterogeneity where meta-analysis was even possible. It is not simply that stigma is locally constructed and interventions are, therefore, locally tailored, but stigma and discrimination are complex phenomena that require varied and multicomponent interventions. As such, it is unsurprising that no single component was found to be consistently central to ‘successful’ interventions, and, as evidenced by several successful interventions, multiple pathways to success exist.

Although not statistically significant, the high level of ‘success’ among interventions to address internalized stigma and stigma and discrimination in healthcare settings with a structural component appears important. Given that stigma is a social construct, it is logical that we need to intervene, not only at the individual level where stigma is experienced in order to have a positive impact but also at the structural level. This idea has also come to the fore in recent work on intersectional stigma. Bowleg has noted the lack, in most policy and research, of ‘how the intersection of multiple interlocking identities at the microlevel reflects multiple and interlocking structural-level inequality at the macro levels of society’ [102]. Stigma and discrimination in healthcare settings and in law and policy are examples of macrolevel drivers of inequalities that result in experiences at the individual level.

It is striking that all of the interventions to address internalized stigma targeted rights-holders (most often people with HIV themselves), while interventions to address stigma and discrimination in healthcare settings all targeted duty-bearers, with one exception that also targeted rights-holders. Among the interventions to address stigma and discrimination in law and policy, excluding court decisions and descriptions of the impacts of laws and policies, it was more common for interventions to include attention to both rights-holders and duty-bearers. As noted above, given that stigma is socially constructed, often structurally entrenched, and experienced at the community and individual level, it is intuitive that engaging both rights-holders and duty-bearers would be important to maximize success. Even internalized stigma, experienced at the individual level, is shaped and perpetuated by the actions and attitudes of others, which means that successfully addressing it likely requires involving more people than the individual experiencing it.

While analyses looking at the effects of legal change often examined laws or policies that had been changed years earlier, the median follow-up for internalized stigma and healthcare interventions was 6 months [mean 9.00 (SD 8.01) months]. It also seems important that the sustained impact of interventions to address internalized stigma and stigma and discrimination in healthcare settings be captured. There is a need for using structured, standardized and validated measures to facilitate research and for assessing long-term effects of interventions to address each of the different types of stigma and discrimination reviewed in order to increase our shared understanding of HIV-related stigma and discrimination.

Across interventions to address internalized stigma and stigma and discrimination in healthcare settings, the identified studies tested complex interventions with multiple intervention components. We did not identify individual components associated with intervention success but multiple components were associated with the interventions’ success and a promising combination was identified. Many components and combinations have not yet been tested empirically.

It is widely accepted that meaningful involvement of people with HIV in all HIV-related interventions is a cornerstone of good practice. It was, therefore, surprising that this review did not find articles explicitly noting the involvement of people with HIV to be associated with intervention success. This raises questions about what authors include in (and exclude from) peer-reviewed articles with limited word counts. Authors tend to report on what they did rather than how they did it – yet the process of any intervention is critical to its potential success [103]. Process evaluation can help fill this knowledge gap on success factors in interventions to address HIV-related stigma.

The largest evidence base was found in relation to interventions to address stigma and discrimination in healthcare settings. Relatively few interventions to address stigma and discrimination in law and policy were identified. The interventions identified were heterogeneous in activities and outcomes precluding any quantitative analysis. This imbalance can help direct future research to help fill existing gaps.

Perhaps reflecting the limited attention to interventions at the structural level, the evidence base linking legal and policy interventions to HIV-related outcomes remains weak. In general, laws that promote nondiscrimination were found to be empowering and laws that criminalize HIV exposure or behaviors relevant to HIV risk detrimental, but the importance of knowledge and implementation of the law in both cases was insufficiently highlighted: laws only have an impact insofar as they are implemented and people know about them.

The geographic distribution of interventions varies by the type of stigma and discrimination being addressed. For internalized stigma and stigma and discrimination in law and policy, around a quarter of studies were carried out in the USA, with the rest in low-income and middle-income countries, including primarily countries in Africa and South East Asia. For stigma and discrimination in healthcare settings, studies reviewed were almost exclusively set in low-income and middle-income countries, mostly China, India, Malawi and other countries in Africa and Southeast Asia. There is a need for greater diversity in scholarship about interventions to address stigma and discrimination across geographies, in different domains, and from different disciplinary perspectives.

Despite the relative recency of the studies included in this review, we changed some language in the evidence table as it fell outside guidelines for acceptable terminology. Although some authors clearly prioritize this, it is important that people-first language be used consistently in research tools and academic publications, particularly those relating to stigma and discrimination. Authors, reviewers and editors all have a role to play in this.

### Limitations

This systematic review only included English-language articles. We adapted eligibility criteria to the type of intervention and available body of evidence, for example, legal analyses did not have to report on a control group. Although appropriate for a systematic review, this made the different areas more difficult to compare. In addition, we used a best evidence strategy throughout the review and used the longest reported follow-up to determine the effects of the intervention. Although ensuring a consistent approach to the evidence documentation, this may have masked initial but temporary success of the interventions. Finally, for the meta-analyses, only studies that reported sufficient detail to determine effect sizes could be considered for the effect estimates.

The component analysis relied on the interventions as described in the publications. The identified interventions were complex, and some interventions may have included components that were not explicitly reported but were nonetheless present and could not be considered in the analysis. The component analysis also relied on the empirical cases of the studies identified through searches rather than allowing for purposefully sampling, which may have limited the conclusions. Relevant data may have been missing from publications.

The co-existence and interrelationship between HIV-related stigma and other devaluing attitudes related to, for example, drug use, sex work, sexual orientation and/or gender identity that affect populations disproportionately affected by HIV is critical, but systematic study of these interactions is beyond the scope of this review.

In conclusion, addressing stigma and discrimination requires action at multiple levels, from the individual to the structural, that involves rights-holders and duty-bearers alike. Although there is no single ‘success factor’ for interventions to address HIV-related stigma and discrimination, multiple pathways to success exist. Furthermore, although interventions must be context-specific, including community intervention, education, counselling, involvement of a support person, and an HIV medical specialist is a promising combination of interventions. The meaningful involvement of people with HIV in all interventions remains an essential component but insufficiently documented. People-first language should also be standard in all publications. The evidence base for interventions to address stigma and discrimination in laws and policies is the thinnest and requires the most strengthening moving forward. However, across a range of settings and diverse populations, promising interventions have been identified that have positive impacts on all three types of stigma and discrimination studied. This evidence base provides a strong foundation for action. It must be built upon and brought to scale to help reach global HIV-related targets and, most importantly, improve the health and quality of life for people with HIV around the world.

## Acknowledgements

The systematic review protocol was peer-reviewed by international content experts and a representative of the community of persons with HIV to ensure that the review asks the right questions. Stakeholders were asked to review the draft review to ensure that all relevant frameworks, measures, and intervention evaluations have been captured and that the review contributes meaningfully to the knowledge base and to ensure that the evidence review is as impactful as possible. The review is registered in PROSPERO, and the data are available in the Agency for Healthcare Research and Quality Systematic Review Data Repository. We thank Brent Allan, and members of the Heart of Stigma country teams for helpful comments.

This work was supported, in whole or in part, by the Bill & Melinda Gates Foundation (INV-004364). Open access publication was supported by the International AIDS Society.

### Conflicts of interest

There are no conflicts of interest.

## Supplementary Material

Supplemental Digital Content

## References

[1] Joint United Nations Programme on HIV/AIDS. Reduction of HIV-related stigma and discrimination: guidance note. Geneva, Switzerland: UNAIDS; 2014.

[2] Stangl AL Lloyd JK Brady LM Holland CE Baral S. A systematic review of interventions to reduce HIV-related stigma and discrimination from 2002 to 2013: how far have we come?. *J Int AIDS Soc* 2013; 16: (3 Suppl 2): 18734. 24242268 10.7448/IAS.16.3.18734 PMC3833106

[3] UNAIDS. Evidence for eliminating HIV-related stigma and discrimination. Gemeva. Switzerland: Joint United Nations Programme on HIV/AIDS; 2020.

[4] United Nations General Assembly. Political Declaration on HIV and AIDS: Ending Inequalities and Getting on Track to End AIDS by 2030. A/RES/75/284. Geneva, Switzerland: United Nations; 2021.

[5] Ferguson L Gruskin S Bolshakova M Yagyu S Fu N Cabrera N. Frameworks and measures for HIV-related internalized stigma, stigma and discrimination in healthcare and in laws and policies: a systematic review. *J Int AIDS Soc* 2022; 25 Suppl 1:e25915. 35818866 10.1002/jia2.25915 PMC9274352

[6] Hempel S Ferguson L Bolshakova M Yagyu S Fu N Motala A Gruskin S. Frameworks, measures, and interventions for HIV-related internalised stigma and stigma in healthcare and laws and policies: systematic review protocol. *BMJ Open* 2021; 11:e053608. 10.1136/bmjopen-2021-053608 PMC8663079 34887280

[7] Hempel S, Ferguson L, Bolshakova M, Yagyu S, Motala S, Gruskin S. Getting to the Heart of Stigma: Systematic Review Protocol. PROSPERO 2021 CRD42021249348. 2021. Available at: https://www.crd.york.ac.uk/PROSPERO/display_record.php?RecordID=249348 [Accessed 5 May 2022]

[8] PRISMA: transparent reporting of systematic reviews and meta-analyses. Available at: http://www.prisma-statement.org/ [Accessed 5 May 2022]

[9] Agency for Healthcare Research and Quality. SRDR systematic review data repository plus. Available at: https://srdrplus.ahrq.gov/ [Accessed 5 May 2022]

[10] UNAIDS. Key populations. Available at: https://www.unaids.org/en/topic/key-populations. [Accessed 12 January 2021]

[11] Global Network Of People Living with HIV (GNP+). The people living with HIV Stigma Index. 2008. Available at: https://www.stigmaindex.org/. [Accessed 22 February 2021]

[12] Sterne JA Hernan MA Reeves BC Savovic J Berkman ND Viswanathan M. ROBINS-I: a tool for assessing risk of bias in nonrandomised studies of interventions. *BMJ* 2016; 355:i4919. 27733354 10.1136/bmj.i4919 PMC5062054

[13] Cochrane Methods Bias. RoB 2: A revised Cochrane risk-of-bias tool for randomized trials. Available at: https://methods.cochrane.org/bias/resources/rob-2-revised-cochrane-risk-bias-tool-randomized-trials. [Accessed 12 January 2020]

[14] Ma PHX Chan ZCY Loke AY. Self-stigma reduction interventions for people living with HIV/AIDS and their families: a systematic review. *AIDS Behav* 2019; 23:707–741. 30298241 10.1007/s10461-018-2304-1

[15] Pantelic M Steinert JI Park J Mellors S Murau F. ’Management of a spoiled identity’: systematic review of interventions to address self-stigma among people living with and affected by HIV. *BMJ Glob Health* 2019; 4:e001285. 10.1136/bmjgh-2018-001285 PMC6441299 30997170

[16] Geter A Herron AR Sutton MY. HIV-related stigma by healthcare providers in the United States: a systematic review. *AIDS Patient Care STDS* 2018; 32:418–424. 30277814 10.1089/apc.2018.0114 PMC6410696

[17] Feyissa GT Lockwood C Woldie M Munn Z. Reducing HIV-related stigma and discrimination in healthcare settings: a systematic review of quantitative evidence. *PLoS One* 2019; 14:e0211298. 30682131 10.1371/journal.pone.0211298 PMC6347272

[18] Nyblade L Stockton MA Giger K Bond V Ekstrand ML Lean RM. Stigma in health facilities: why it matters and how we can change it. *BMC Med* 2019; 17:25. 30764806 10.1186/s12916-019-1256-2 PMC6376713

[19] Feyissa GT Lockwood C Woldie M Munn Z. Reducing HIV-related stigma and discrimination in healthcare settings: a systematic review of guidelines, tools, standards of practice, best practices, consensus statements and systematic reviews. *J Multidiscip Healthc* 2018; 11:405–416. 30214222 10.2147/JMDH.S170720 PMC6118284

[20] Kaladharan S Daken K Mullens AB Durham J. Tools to measure HIV knowledge, attitudes & practices (KAPs) in healthcare providers: a systematic review. *AIDS Care* 2020; 33:1500–1506. 32964738 10.1080/09540121.2020.1822502

[21] Pleuhs B Quinn KG Walsh JL Petroll AE John SA. Healthcare provider barriers to HIV pre-exposure prophylaxis in the United States: a systematic review. *AIDS Patient Care STDS* 2020; 34:111–123. 32109141 10.1089/apc.2019.0189 PMC7087402

[22] Cheema E Abbas A Al-Hamid A. Healthcare-related factors affecting the management of HIV infected patients: a systematic review of qualitative evidence. *Int J STD AIDS* 2019; 30:1350–1361. 31739748 10.1177/0956462419875357

[23] Phillips JC Caine V Dewart G de Padua A dela Cruz AM Rickards T. Teaching HIV-specific content for prelicensure nursing and health professions students: a review and synthesis. *AIDS Care Psychol Socio-Med Asp Aids-HIV* 2018; 30:1614–1621. 10.1080/09540121.2018.1510108 30112926

[24] Siegfried N, Beanland R. Systematic review on the evidence-base for eliminating stigma and discrimination in healthcare settings (prepared for CDC and ICAP). April 2017. 1–236.

[25] Brown L Macintyre K Trujillo L. Interventions to reduce HIV/AIDS stigma: what have we learned?. *AIDS Educ Prev* 2003; 15:49–69. 12627743 10.1521/aeap.15.1.49.23844

[26] Ragin CC. The comparative method: moving beyond qualitative and quantitative strategies. California: University of California Press; 1989.

[27] Oana IE Schneider CQ Thomann E. Qualitative comparative analysis using R: a beginner's guide. Cambridge: Cambridge University Press; 2021.

[28] GRADE working group. GRADE. Available at: https://www.gradeworkinggroup.org/. [Accessed 12 January 2020]

[29] Adia AC Restar AJ Lee CJ Payawal MP Quilantang MI Nazareno J Operario D. Sword and Shield: Perceptions of law in empowering and protecting HIV-positive men who have sex with men in Manila, Philippines. *Global Public Health* 2020; 15:52–63. 31134838 10.1080/17441692.2019.1622762 PMC12273262

[30] Arora SS Jyoti S Chakravarty S. Effectiveness of an empowering programme on student nurses’ understanding and beliefs about HIV/AIDS. *Int J Nurs Educ* 2014; 6:88–92.

[31] Barroso J Relf MV Williams MS Arscott J Moore ED Caiola C Silva SG. A randomized controlled trial of the efficacy of a stigma reduction intervention for HIV-infected women in the Deep South. *AIDS Patient Care STDS* 2014; 28:489–498. 25084499 10.1089/apc.2014.0014 PMC4135326

[32] Batchelder AW Moskowitz JT Jain J Cohn M Earle MA Carrico AW. A novel technology-enhanced internalized stigma and shame intervention for HIV-positive persons with substance use disorders. *Cogn Behav Pract* 2020; 27:55–69. 33790528 10.1016/j.cbpra.2019.03.001 PMC8009529

[33] Bauermeister JA Muessig KE LeGrand S Flores DD Choi SK Dong W. HIV and sexuality stigma reduction through engagement in online forums: results from the HealthMPowerment Intervention. *AIDS Behav* 2019; 23:742–752. 30121727 10.1007/s10461-018-2256-5 PMC6379154

[34] Bhatta DN Liabsuetrakul T. Efficacy of a social self-value empowerment intervention to improve quality of life of HIV infected people receiving antiretroviral treatment in Nepal: a randomized controlled trial. *AIDS Behav* 2016; 21:1620–1631. 10.1007/s10461-016-1546-z PMC5422450 27613646

[35] Bogart LM Barreras JL Gonzalez A Klein DJ Marsh T Agniel D. Pilot randomized controlled trial of an intervention to improve coping with intersectional stigma and medication adherence among HIV-positive Latinx sexual minority men. *AIDS Behav* 2021; 25:1647–1660. 33231847 10.1007/s10461-020-03081-z PMC8084890

[36] Brito M. On an alternative to a punitive standard in response to a more modern understanding of the HIV/AIDS epidemic in Florida notes and comments. *Nova L Rev* 2015; 40:285–348.

[37] Chidrawi HC Greeff M Temane QM. HIV stigma experiences and stigmatisation before and after an intervention. *Health SA Gesondheid* 2016; 21:196–205.

[38] Denison JA Burke VM Miti S Nonyane BAS Frimpong C Merrill KG. Project YES! Youth engaging for success: a randomized controlled trial assessing the impact of a clinic-based peer mentoring program on viral suppression, adherence and internalized stigma among HIV-positive youth (15-24 years) in Ndola, Zambia. *PLoS One* 2020; 15:e0230703. 32240186 10.1371/journal.pone.0230703 PMC7117673

[39] Denison JA Packer C Nyambe N Hershow RB Caldas S Miti S. Family Connections randomized controlled trial: assessing the feasibility and acceptability of an intervention with adolescents living with HIV and their caregivers in Ndola, Zambia. *AIDS Care* 2021; 34:459–468. 33764845 10.1080/09540121.2021.1902935 PMC12959607

[40] France NF Macdonald SHF Conroy RR Chiroro P Cheallaigh DN Nyamucheta M. ‘We are the change’—An innovative community-based response to address self-stigma: a pilot study focusing on people living with HIV in Zimbabwe. *PLoS One* 2019; 14:24. 10.1371/journal.pone.0210152 PMC6373928 30759114

[41] Galletly CL Pinkerton SD DiFranceisco W. A quantitative study of Michigan's criminal HIV exposure law. *AIDS Care* 2012; 24:174–179. 21861631 10.1080/09540121.2011.603493 PMC3428201

[42] Go VF Frangakis C Minh NL Latkin C Ha TV Mo TT. Efficacy of a multilevel intervention to reduce injecting and sexual risk behaviors among HIV-infected people who inject drugs in Vietnam: a four-arm randomized controlled trial. *PLoS One* 2015; 10:e0125909. 26011427 10.1371/journal.pone.0125909 PMC4444299

[43] Gruskin S Safreed-Harmon K Ezer T Gathumbi A Cohen J Kameri-Mbote P. Access to justice: evaluating law, health and human rights programmes in Kenya. *J Int AIDS Soc* 2013; 16: (3 Suppl 2): 18726. 24242267 10.7448/IAS.16.3.18726 PMC3833108

[44] Harper GW Lemos D Hosek SG. Stigma reduction in adolescents and young adults newly diagnosed with HIV: findings from the Project ACCEPT intervention. *AIDS Patient Care STDS* 2014; 28:543–554. 25216106 10.1089/apc.2013.0331 PMC4183905

[45] Hickey MD Ouma GB Mattah B Pederson B DesLauriers NR Mohamed P. The Kanyakla study: randomized controlled trial of a microclinic social network intervention for promoting engagement and retention in HIV care in rural western Kenya. *PLoS One* 2021; 16:e0255945. 34516557 10.1371/journal.pone.0255945 PMC8437299

[46] Kaponda CP Jere DL Chimango JL Chimwaza AF Crittenden KS Kachingwe SI. Impacts of a peer-group intervention on HIV-related knowledge, attitudes, and personal behaviors for urban hospital workers in Malawi. *J Assoc Nurses AIDS Care* 2009; 20:230–242. 19427600 10.1016/j.jana.2008.12.005 PMC4177099

[47] Lane HH. Kiyutin v. Russia: The European Court of Human Rights Acknowledges the Need for Protection of a Class of Individuals with HIV/AIDS Recent Developments. *Tul J Int’l & Comp L* 2011; 20:505–518.

[48] Li L Wu Z Liang LJ Lin C Guan J Jia M. Reducing HIV-related stigma in healthcare settings: a randomized controlled trial in China. *Am J Public Health* 2013; 103:286–292. 23237175 10.2105/AJPH.2012.300854 PMC3556241

[49] Lifson AR Workneh S Hailemichael A Demisse W Slater L Shenie T. Implementation of a peer HIV Community Support Worker Program in Rural Ethiopia to Promote Retention in Care. *J Int Assoc Provid AIDS Care* 2017; 16:75–80. 26518590 10.1177/2325957415614648

[50] Lohiniva AL Benkirane M Numair T Mahdy A Saleh H Zahran A. HIV stigma intervention in a low-HIV prevalence setting: a pilot study in an Egyptian healthcare facility. *AIDS Care* 2016; 28:644–652. 26717980 10.1080/09540121.2015.1124974

[51] Lowther K Harding R Simms V Gikaara N Ahmed A Ali Z. Effect of participation in a randomised controlled trial of an integrated palliative care intervention on HIV-associated stigma. *AIDS Care* 2018; 30:1180–1188. 29663828 10.1080/09540121.2018.1465176

[52] Mahajan AP Kinsler JJ Cunningham WE James S Makam L Manchanda R. Does the Centers for Disease Control and Prevention's Recommendation of opt-out HIV screening impact the effect of stigma on HIV test acceptance?. *AIDS Behav* 2016; 20:107–114. 26462670 10.1007/s10461-015-1222-8

[53] Mak WW Cheng SS Law RW Cheng WW Chan F. Reducing HIV-related stigma among health-care professionals: a game-based experiential approach. *AIDS Care* 2015; 27:855–859. 25671591 10.1080/09540121.2015.1007113

[54] Maluccio JA Wu F Rokon RB Rawat R Kadiyala S. Assessing the impact of food assistance on stigma among people living with HIV in Uganda using the HIV/AIDS Stigma Instrument-PLWA (HASI-P). *AIDS Behav* 2017; 21:766–782. 27372803 10.1007/s10461-016-1476-9

[55] Masquillier C Wouters E Mortelmans D le Roux Booysen F. The impact of community support initiatives on the stigma experienced by people living with HIV/AIDS in South Africa. *AIDS Behav* 2015; 19:214–226. 25129453 10.1007/s10461-014-0865-1

[56] Mbeba MM Kaponda CP Jere DL Kachingwe SI Crittenden KS McCreary LL. Peer group intervention reduces personal HIV risk for Malawian health workers. *J Nurs Scholarsh* 2011; 43:72–81. 21342427 10.1111/j.1547-5069.2011.01384.x PMC3073810

[57] Miller RL Rutledge J Ayala G. Breaking down barriers to HIV care for gay and bisexual men and transgender women: the Advocacy and Other Community Tactics (ACT) Project. *AIDS Behav* 2021; 25:2551–2567. 33730253 10.1007/s10461-021-03216-w PMC7966621

[58] Mockiene V Suominen T Valimaki M Razbadauskas A Martinkenas A Caplinskas S. The impact of an education intervention to change nurses’ HIV-related knowledge and attitudes in Lithuania: a randomized controlled trial. *J Assoc Nurses AIDS Care* 2011; 22:140–149. 21123087 10.1016/j.jana.2010.07.006

[59] Nestadt DF Saisaengjan C McKay MM Bunupuradah T Pardo G Lakhonpon S. CHAMP+ Thailand: Pilot randomized control trial of a family-based psychosocial intervention for perinatally HIV-infected early adolescents. *AIDS Patient Care and STDs* 2019; 33:227–236. 31067121 10.1089/apc.2019.0021 PMC6531900

[60] Norr KF Ferrer L Cianelli R Crittenden KS Irarrazabal L Cabieses B. Peer group intervention for HIV prevention among health workers in Chile. *J Assoc Nurses AIDS Care* 2012; 23:73–86. 21497113 10.1016/j.jana.2011.02.001 PMC3140569

[61] Nyamathi A Ekstrand M Salem BE Sinha S Ganguly KK Leake B. Impact of Asha intervention on stigma among rural Indian women with AIDS. *West J Nurs Res* 2013; 35:867–883. 23539322 10.1177/0193945913482050 PMC3725982

[62] Nyblade L Addo NA Atuahene K Alsoufi N Gyamera E Jacinthe S. Results from a difference-in-differences evaluation of health facility HIV and key population stigma-reduction interventions in Ghana. *J Int AIDS Soc* 2020; 23:e25483. 32329153 10.1002/jia2.25483 PMC7180216

[63] Nyblade L, Mbuya-Brown RJ, Sabasaba AN, Ezekiel M, Kiwia P, *et al*. Understanding and responding to stigma and discrimination in health facilities in Tanzania. In. Washington, DC: Palladium, Health Policy Plus; 2018.

[64] Odeny TA Penner J Lewis-Kulzer J Leslie H H Shade S B Adero W. Integration of HIV care with primary healthcare services: effect on patient satisfaction and stigma in Rural Kenya. *AIDS Res Treat* 2013; 2013:485715. 23738055 10.1155/2013/485715 PMC3664481

[65] Onyemelukwe C. Discrimination on the basis of HIV status: an analysis of recent developments in Nigerian Law and Jurisprudence. *Int J Discrimination Law* 2017; 17:160–179.

[66] Peltzer K Babayigit S Rodriguez VJ Jean J Sifunda S Jones DL. Effect of a multicomponent behavioural PMTCT cluster randomised controlled trial on HIV stigma reduction among perinatal HIV positive women in Mpumalanga province, South Africa. *Sahara J* 2018; 15:80–88. 30134772 10.1080/17290376.2018.1510787 PMC6116698

[67] Petersen I Hanass Hancock J Bhana A Govender K. A group-based counselling intervention for depression comorbid with HIV/AIDS using a task shifting approach in South Africa: a randomized controlled pilot study. *J Affect Disord* 2014; 158:78–84. 24655769 10.1016/j.jad.2014.02.013

[68] Prinsloo CD Greeff M Kruger A Khumalo IP. HIV stigma experiences and stigmatisation before and after a HIV stigma-reduction community ‘hub’ intervention. *Afr J AIDS Res* 2017; 16:203–213. 28978287 10.2989/16085906.2017.1349683

[69] Pulerwitz J Oanh KT Akinwolemiwa D Ashburn K Nyblade L. Improving hospital-based quality of care by reducing HIV-related stigma: evaluation results from Vietnam. *AIDS Behav* 2015; 19:246–256. 25382350 10.1007/s10461-014-0935-4

[70] Rao D Desmond M Andrasik M Rasberry T Lambert N Cohn SE Simoni J. Feasibility, acceptability, and preliminary efficacy of the unity workshop: an internalized stigma reduction intervention for African American women living with HIV. *AIDS Patient Care STDS* 2012; 26:614–620. 22984780 10.1089/apc.2012.0106 PMC3462391

[71] Rao D Kemp CG Huh D Nevin PE Turan J Cohn SE. Stigma reduction among African American women with HIV: UNITY Health Study. *J Acquir Immune Defic Syndr* 2018; 78:269–275. 29528941 10.1097/QAI.0000000000001673 PMC5997522

[72] Rongkavilit C Wang B Naar-King S Bunupuradah T Parsons JT Panthong A. Motivational interviewing targeting risky sex in HIV-positive young Thai men who have sex with men. *Arch Sex Behav* 2015; 44:329–340. 24668304 10.1007/s10508-014-0274-6 PMC4177013

[73] Schwartz SR Nowak RG Orazulike I Keshinro B Ake J Kennedy S. The immediate effect of the Same-Sex Marriage Prohibition Act on stigma, discrimination, and engagement on HIV prevention and treatment services in men who have sex with men in Nigeria: analysis of prospective data from the TRUST cohort. *Lancet HIV* 2015; 2:e299–e306. 26125047 10.1016/S2352-3018(15)00078-8 PMC4481876

[74] Sears B, Cooper C, Younai FS, Donohoe T. HIV discrimination in dental care: results of a testing study in Los Angeles County. 2012.

[75] Shamsaei F Tahour N Sadeghian E. Effect of stress management training on stigma and social phobia in HIV-positive women. *J Int Assoc Provid AIDS Care* 2020; 19: 2325958220918953. 10.1177/2325958220918953 PMC7153174 32274969

[76] Singh RJ Sarna A Schensul JJ Mahapatra B Ha T Schensul SL. A multilevel intervention to reduce stigma among alcohol consuming men living with HIV receiving antiretroviral therapy: findings from a randomized control trial in India. *AIDS* 2020; 34: (Suppl 1): S83–S92. 32881797 10.1097/QAD.0000000000002604

[77] Siraprapasiri T Srithanaviboonchai K Chantcharas P Suwanphatthana N Ongwandee S Khemngern P. Integration and scale-up of efforts to measure and reduce HIV-related stigma: the experience of Thailand. *AIDS* 2020; 34: (Suppl 1): S103–S114. 32881799 10.1097/QAD.0000000000002586

[78] Skinta MD Lezama M Wells G Dilley JW. Acceptance and compassion-based group therapy to reduce HIV stigma. *Cogn Behav Pract* 2015; 22:481–490.

[79] Sommerland N Masquillier C Rau A Engelbrecht M Kigozi G Pliakas T. Reducing HIV- and TB-Stigma among healthcare co-workers in South Africa: results of a cluster randomised trial. *Soc Sci Med* 2020; 266:113450. 33126096 10.1016/j.socscimed.2020.113450

[80] Srinivasan K Heylen E Raj T Nyblade L Devadass D Pereira M. Reduction in stigma drivers partially mediates the effect of a stigma reduction intervention among nursing students in India: the DriSti Cluster Randomized Controlled Trial. *J Acquir Immune Defic Syndr* 2021; 86:182–190. 33105394 10.1097/QAI.0000000000002543 PMC7884286

[81] Step MM Knight K McMillen Smith J Lewis SA Russell TJ Avery AK. Positive peers mobile application reduces stigma perception among young people living with HIV. *Health Promot Pract* 2020; 21:744–754. 32757838 10.1177/1524839920936244

[82] The Center for HIV Law & Policy. HIV Criminalization in the United States: A Sourcebook on State and Federal HIV Criminal Law and Practice. 3rd ed; 2022. Available at: https://www.hivlawandpolicy.org/sites/default/files/HIV%20Criminalization%20in%20the%20U.S.%20A%20Sourcebook%20on%20State%20Fed%20HIV%20Criminal%20Law%20and%20Practice%20022722.pdf [Accessed 22 June 2023]

[83] Tsai AC Bangsberg DR Bwana M Haberer JE Frongillo EA Muzoora C. How does antiretroviral treatment attenuate the stigma of HIV? Evidence from a cohort study in rural Uganda. *AIDS Behav* 2013; 17:2725–2731. 23670710 10.1007/s10461-013-0503-3 PMC3773278

[84] Tsai AC Hatcher AM Bukusi EA Weke E Lemus Hufstedler L Dworkin SL. A livelihood intervention to reduce the stigma of HIV in rural Kenya: longitudinal qualitative study. *AIDS Behav* 2017; 21:248–260. 26767535 10.1007/s10461-015-1285-6 PMC5444205

[85] Tshabalala J Visser M. Developing a cognitive behavioural therapy model to assist women to deal with HIV and stigma. *S Afr J Psychol* 2011; 41:17–28.

[86] UNAIDS. Legal and policy trends impacting people living with HIV and key populations in Asia and the Pacific 2014-2019. 2021. Available at: https://www.unaids.org/sites/default/files/media_asset/legal-and-policy-trends-asia-pacific_en.pdf [Accessed 20 April 2021]

[87] UNDP. Evaluation of the ‘Building capacity for reform of HIV-related law and policy in Jamaica’ Project’ 2016. Available at: https://erc.undp.org/evaluation/evaluations/detail/7308 [Accessed 20 April 2021]

[88] UNDP. End-term review HIV Law SIDA Project (Africa). 2019. Available at: https://erc.undp.org/evaluation/evaluations/detail/9379 [Accessed 20 April 2021]

[89] UNDP. Global Project for the Evaluation of the Global Commission on HIV and the Law. 2021. Available at: https://erc.undp.org/evaluation/evaluations/detail/9380# [Accessed 20 April 2021]

[90] Uys L Chirwa M Kohi T Greeff M Naidoo J Makoae L. Evaluation of a health setting-based stigma intervention in five African countries. *AIDS Patient Care STDS* 2009; 23:1059–1066. 20025515 10.1089/apc.2009.0085 PMC2832642

[91] Van Tam V Larsson M Pharris A Diedrichs B Nguyen HP Nguyen CT. Peer support and improved quality of life among persons living with HIV on antiretroviral treatment: a randomised controlled trial from north-eastern Vietnam. *Health Qual Life Outcomes* 2012; 10:53. 22606977 10.1186/1477-7525-10-53 PMC3491019

[92] Varas-Díaz N Neilands TB Cintrón-Bou F Marzán-Rodríguez M Santos-Figueroa A Santiago-Negrón S. Testing the efficacy of an HIV stigma reduction intervention with medical students in Puerto Rico: the SPACES project. *J Int AIDS Soc* 2013; 16: (3 Suppl 2): 18670. 24242260 10.7448/IAS.16.3.18670 PMC3833102

[93] Wagner GJ Ghosh-Dastidar B Garnett J. Impact of HIV antiretroviral therapy on depression and mental health among clients with HIV in Uganda. *Psychosom Med* 2012; 74:883–890. 22923701 10.1097/PSY.0b013e31826629db

[94] Watt MH Aronin EH Maman S Thielman N Laiser J John M. Acceptability of a group intervention for initiates of antiretroviral therapy in Tanzania. *Glob Public Health* 2011; 6:433–446. 20635269 10.1080/17441692.2010.494162 PMC3530384

[95] Watt MH Minja L Knettel BA Mwamba RN Osaki H Ngocho JS. Pilot outcomes of Maisha: an HIV stigma reduction intervention developed for antenatal care in Tanzania. *AIDS Behav* 2020; 25:1171–1184. 33180253 10.1007/s10461-020-03093-9 PMC7979435

[96] Wu S Li L Wu Z Liang LJ Cao H Yan Z Li J. A brief HIV stigma reduction intervention for service providers in China. *AIDS Patient Care STDS* 2008; 22:513–520. 18462076 10.1089/apc.2007.0198 PMC2700336

[97] Yigit I Modi RA Weiser SD Johnson MO Mugavero MJ Turan JM Turan B. Effects of an intervention on internalized HIV-related stigma for individuals newly entering HIV care. *AIDS* 2020; 34 Suppl 1: (Suppl 1): S73–S82. 32881796 10.1097/QAD.0000000000002566 PMC8075078

[98] Yiu JW Mak WW Ho WS Chui YY. Effectiveness of a knowledge-contact program in improving nursing students’ attitudes and emotional competence in serving people living with HIV/AIDS. *Soc Sci Med* 2010; 71:38–44. 20430503 10.1016/j.socscimed.2010.02.045

[99] Hightow-Weidman L, Legrand S, Simmons R, Egger J, Choi SK, Muessig KE. **healthMpowerment: Effects of a mobile phone-optimized, Internet-based intervention on condomless anal intercourse among young black men who have sex with men and transgender women.** *9th IAS Conference on HIV Science*. Paris, France; 23–26 July 2017.

[100] Prinsloo CD Greeff M. A Community ‘Hub’ network intervention for HIV stigma reduction: a case study. *J Assoc Nurses AIDS Care* 2016; 27:166–179. 26627447 10.1016/j.jana.2015.10.007

[101] Fabian K Molina Y Kemp CG Nevin PE McCoy K Simoni JM. Internalized HIV-related stigma and breast health beliefs among African-American women receiving care for HIV in the USA. *J Racial Ethn Health Disparities* 2020; 7:45–51. 31452148 10.1007/s40615-019-00632-6 PMC6980483

[102] Bowleg L. The problem with the phrase women and minorities: intersectionality-an important theoretical framework for public health. *Am J Public Health* 2012; 102:1267–1273. 22594719 10.2105/AJPH.2012.300750 PMC3477987

[103] Pinnock H Epiphaniou E Sheikh A Griffiths C Eldridge S Craig P Taylor SJ. Developing standards for reporting implementation studies of complex interventions (StaRI): a systematic review and e-Delphi. *Implement Sci* 2015; 10:42. 25888928 10.1186/s13012-015-0235-z PMC4393562

