# Supplementary material for: Systematic review and quantitative and qualitative comparative analysis of interventions to address HIV-related stigma and discrimination
Source: AIDS. 2023 Jun 19;37(13):1919–39. doi: 10.1097/QAD.0000000000003628 (PMC10552822; doi:10.1097/QAD.0000000000003628)
Supplement: Supplemental Digital Content [file aids-37-1919-s001.docx]

# Appendix

## Key Definitions

- Stigma refers to negative beliefs and/or attitudes about HIV.
- Discrimination refers to the institutional behaviors that result from negative attitudes or beliefs about HIV.
- Internalized stigma (self-stigma) refers to a person living with HIV internalizing negative attitudes associated with HIV and accepting these as applicable to themselves.
- Stigma and discrimination in healthcare refers to negative beliefs and behaviors based on perceived or actual HIV status experienced in healthcare delivery settings.
- Discrimination in laws and policies refers to distinctions, exclusion or restrictions as written or applied based on perceived HIV status or membership of a group that is vulnerable to HIV.

## Search Strategy

Targeted search strategies for each key question combined free text search terms with controlled vocabulary of the individual databases. The searches were designed, executed, and documented by an Evidence-based Practice Center librarian. The multiple sources and interdisciplinary approach aimed to reduce the introduction of selection bias in the literature review.

PUBMED

10/4/2021

797 results

("social stigma"[MeSH Terms] OR ("social"[All Fields] AND "stigma"[All Fields]) OR "social stigma"[All Fields] OR "stigma"[All Fields] OR "stigmas"[All Fields] OR "stigma s"[All Fields] OR ("discriminabilities"[All Fields] OR "discriminability"[All Fields] OR "discriminable"[All Fields] OR "discriminably"[All Fields] OR "discriminance"[All Fields] OR "discriminant"[All Fields] OR "discriminants"[All Fields] OR "discriminate"[All Fields] OR "discriminated"[All Fields] OR "discriminates"[All Fields] OR "discriminating"[All Fields] OR "discrimination, psychological"[MeSH Terms] OR ("discrimination"[All Fields] AND "psychological"[All Fields]) OR "psychological discrimination"[All Fields] OR "discrimination"[All Fields] OR "discriminations"[All Fields] OR "discriminative"[All Fields] OR "discriminatively"[All Fields] OR "discriminator"[All Fields] OR "discriminators"[All Fields])) AND ("hiv"[MeSH Terms] OR "hiv"[All Fields] OR ("acquired immunodeficiency syndrome"[MeSH Terms] OR ("acquired"[All Fields] AND "immunodeficiency"[All Fields] AND "syndrome"[All Fields]) OR "acquired immunodeficiency syndrome"[All Fields] OR "aids"[All Fields])) AND ("randomized controlled trial"[Publication Type] OR "randomized controlled trials as topic"[MeSH Terms] OR "randomized controlled trial"[All Fields] OR "randomised controlled trial"[All Fields] OR ("prospective studies"[MeSH Terms] OR ("prospective"[All Fields] AND "studies"[All Fields]) OR "prospective studies"[All Fields] OR ("prospective"[All Fields] AND "study"[All Fields]) OR "prospective study"[All Fields]) OR ("clinical trial"[Publication Type] OR "clinical trials as topic"[MeSH Terms] OR "clinical trial"[All Fields])) AND 2008/01/01:3000/12/31[Date - Publication] AND "english"[Language]

PsycINFO

10/4/2021

274 results

(ti(stigma OR discrimin*) OR ab(stigma OR discrimin*) OR su(stigma OR discrimin*)) AND (ti(HIV OR "acquired immunodeficiency syndrome") OR ab(HIV OR "acquired immunodeficiency syndrome") OR su(HIV OR "acquired immunodeficiency syndrome")) AND (ti("randomized controlled trial" OR "randomized controlled trials" OR "RCT" OR "RCTs" OR "prospective study" OR "prospective studies" OR "clinical trial" OR "clinical trials") OR ab("randomized controlled trial" OR "randomized controlled trials" OR "RCT" OR "RCTs" OR "prospective study" OR "prospective studies" OR "clinical trial" OR "clinical trials") OR su("randomized controlled trial" OR "randomized controlled trials" OR "RCT" OR "RCTs" OR"prospective study" OR "prospective studies" OR "clinical trial" OR "clinical trials")) AND (la.exact("ENG") AND pd(20080101-20211005))

Web of Science

10/4/2021

94 results

(((TS=(stigma OR discrimin*)) AND TS=(HIV OR "acquired immunodeficiency syndrome" ))) AND TS=("randomized controlled trial" OR "randomized controlled trials" OR "prospective study" OR "prospective studies" OR "clinical trial" OR "clinical trials") and Psychology or Health Care Sciences Services (Research Areas)

Editions = BKCI-SSH, BKCI-S, ESCI, SCI-EXPANDED, SSCI

2008-01-01 to 2021-10-05

PAIS

10/4/2021

13 results

noft(hiv OR "acquired immunodeficiency syndrome") AND noft(stigma OR discrimin*) AND noft("randomized controlled trial" OR "randomized controlled trials" OR "prospective study" OR “prospective studies" OR "clinical trial" OR "clinical trials")

Limits applied:

Date: After January 01 2008

Language:

English

HeinOnline

Limits applied

40 results

((((stigma or discrimin*) AND (HIV OR "acquired immunodeficiency syndrome")) AND ("randomized controlled trial" OR "randomized controlled trials" OR "prospective study" OR "prospective studies" OR "clinical trial" OR "clinical trials")))

AND

Pathfinder Subject: AIDS

AND

Publication range: 2008-2021

LegalTrac

10/4/201

0 results

HIV Legal Network

10/4/2021

0 results

SAGE: ENGAGING AFRICAN, CARIBBEAN AND BLACK COMMUNITIES IN ADDRESSING HIV

10/4/2021

0 results

### Scoping searches

PubMed

Date: 1/11/2021

Yield: 112

Terms

("framework"[All Fields] OR "framework s"[All Fields] OR "frameworks"[All Fields] OR "logic model"[All Fields] OR "analytic model"[All Fields]) AND ("stigma"[Title] OR "discrimination"[Title]) AND ("hiv"[MeSH Terms] OR "hiv"[All Fields] OR ("acquired immunodeficiency syndrome"[MeSH Terms] OR ("acquired"[All Fields] AND "immunodeficiency"[All Fields] AND "syndrome"[All Fields]) OR "acquired immunodeficiency syndrome"[All Fields] OR "aids"[All Fields]))

OR

Date: 1/11/2021

Yield: 112

Filter: 2008-date

Terms

("measure*"[Title] OR "test"[Title] OR ("scale s"[All Fields] OR "scaled"[All Fields] OR "scaling"[All Fields] OR "scalings"[All Fields] OR "weights and measures"[MeSH Terms] OR ("weights"[All Fields] AND "measures"[All Fields]) OR "weights and measures"[All Fields] OR "scale"[All Fields] OR "scales"[All Fields]) OR "tool"[All Fields] OR "assess*"[Title] OR ("psychometrical"[All Fields] OR "psychometrically"[All Fields] OR "psychometrics"[MeSH Terms] OR "psychometrics"[All Fields] OR "psychometric"[All Fields])) AND ("stigma"[Title] OR "discrimination"[Title]) AND ("hiv"[MeSH Terms] OR "hiv"[All Fields] OR ("acquired immunodeficiency syndrome"[MeSH Terms] OR ("acquired"[All Fields] AND "immunodeficiency"[All Fields] AND "syndrome"[All Fields]) OR "acquired immunodeficiency syndrome"[All Fields] OR "aids"[All Fields]))

OR

Date: 1/11/2021

Filter: Systematic reviews

Yield: 225

Terms

("social stigma"[MeSH Terms] OR ("social"[All Fields] AND "stigma"[All Fields]) OR "social stigma"[All Fields] OR "stigma"[All Fields] OR "stigmas"[All Fields] OR "stigma s"[All Fields] OR ("discriminabilities"[All Fields] OR "discriminability"[All Fields] OR "discriminable"[All Fields] OR "discriminably"[All Fields] OR "discriminance"[All Fields] OR "discriminant"[All Fields] OR "discriminants"[All Fields] OR "discriminate"[All Fields] OR "discriminated"[All Fields] OR "discriminates"[All Fields] OR "discriminating"[All Fields] OR "discrimination, psychological"[MeSH Terms] OR ("discrimination"[All Fields] AND "psychological"[All Fields]) OR "psychological discrimination"[All Fields] OR "discrimination"[All Fields] OR "discriminations"[All Fields] OR "discriminative"[All Fields] OR "discriminatively"[All Fields] OR "discriminator"[All Fields] OR "discriminators"[All Fields])) AND ("hiv"[MeSH Terms] OR "hiv"[All Fields] OR ("acquired immunodeficiency syndrome"[MeSH Terms] OR ("acquired"[All Fields] AND "immunodeficiency"[All Fields] AND "syndrome"[All Fields]) OR "acquired immunodeficiency syndrome"[All Fields] OR "aids"[All Fields]))

OR

Date: 2/5/2021

Yield: 199 results

Filters: Randomized Controlled Trial, English language, 2008 - 2021

Terms

("social stigma"[MeSH Terms] OR ("social"[All Fields] AND "stigma"[All Fields]) OR "social stigma"[All Fields] OR "stigma"[All Fields] OR "stigmas"[All Fields] OR "stigma s"[All Fields] OR ("discriminabilities"[All Fields] OR "discriminability"[All Fields] OR "discriminable"[All Fields] OR "discriminably"[All Fields] OR "discriminance"[All Fields] OR "discriminant"[All Fields] OR "discriminants"[All Fields] OR "discriminate"[All Fields] OR "discriminated"[All Fields] OR "discriminates"[All Fields] OR "discriminating"[All Fields] OR "discrimination, psychological"[MeSH Terms] OR ("discrimination"[All Fields] AND "psychological"[All Fields]) OR "psychological discrimination"[All Fields] OR "discrimination"[All Fields] OR "discriminations"[All Fields] OR "discriminative"[All Fields] OR "discriminatively"[All Fields] OR "discriminator"[All Fields] OR "discriminators"[All Fields]))

AND

("hiv"[MeSH Terms] OR "hiv"[All Fields] OR ("acquired immunodeficiency syndrome"[MeSH Terms] OR ("acquired"[All Fields] AND "immunodeficiency"[All Fields] AND "syndrome"[All Fields]) OR "acquired immunodeficiency syndrome"[All Fields] OR "aids"[All Fields]))

OR

(systematic reviews)

Date: 5/6/2021

Yield: 235

Publication Date Range: 2008-05/06/2021

Filter: Systematic Review

Terms

Social stigma[MESH terms] OR stigma OR discrimination OR discrimination, psychological[MESH terms]

AND

HIV[Mesh terms] OR hiv OR acquired immunodeficiency syndrome[MESH terms] OR "acquired immunodeficiency syndrome" OR "AIDS"[Title/Abstract]

PsycINFO

(Frameworks; limited ‘framework’ terms to title field, 2008-2021 publication date range)

Date: 3/26/2021

Yield: 41

Terms

(ti(stigma OR discrimin*) OR ab(stigma OR discrimin*) OR su(stigma OR discrimin*)) AND (ti(HIV OR "acquired immunodeficiency syndrome") OR ab(HIV OR "acquired immunodeficiency syndrome") OR su(HIV OR "acquired immunodeficiency syndrome")) AND ti(framework OR frameworks OR "logic model" OR "analytic model") AND la.exact("ENG")

OR

(Measures; limited ‘measures’ terms to title field)

Date: 3/26/2021

Yield: 116

Terms

(ti(stigma OR discrimin*) OR ab(stigma OR discrimin*) OR su(stigma OR discrimin*)) AND (ti(HIV OR "acquired immunodeficiency syndrome") OR ab(HIV OR "acquired immunodeficiency syndrome") OR su(HIV OR "acquired immunodeficiency syndrome")) AND ti(measure OR measures OR scale OR scales OR tool OR tools) AND (la.exact("ENG") AND pd(20080101-20211231))

OR

Date: 3/19/2021

Yield: 58

Terms

((ti(stigma OR discrimin*) OR ab(stigma OR discrimin*) OR su(stigma OR discrimin*)) AND (ti(HIV OR "acquired immunodeficiency syndrome") OR ab(HIV OR "acquired immunodeficiency syndrome") OR su(HIV OR "acquired immunodeficiency syndrome")))

AND

(la.exact("ENG") AND me.exact("Prospective Study" OR "Clinical Trial")

AND

pd(20080101-20211231))

OR

Date: 5/6/2021

Yield: 100

Publication Date Range: After January 1, 2008

Methodology: Systematic Review

Terms

noft(stigma OR discriminat*) AND noft("hiv" OR "acquired immunodeficiency syndrome" OR "AIDS")

Web of Science

(Framework; omitted conference proceedings, added Research Areas, limited ‘framework’ terms to title field, 2008-2021 publication date range)

Yield: 14

Terms

(TS=(stigma OR discrim*) AND TS=(HIV OR "acquired immunodeficiency syndrome") AND TI=(framework OR frameworks OR "logic model" OR "logic models" OR "analytic model" OR "analytic models")) AND LANGUAGE: (English)

Refined by: RESEARCH AREAS: (HEALTH CARE SCIENCES SERVICES OR PSYCHOLOGY )

Indexes=SCI-EXPANDED, SSCI, BKCI-S, BKCI-SSH, ESCI Timespan=1900-2021

OR

(Measures; omitted conference proceedings, added Research Areas, limited ‘measures’ terms to title field)

Yield: 75

TS=(stigma OR discrim*) AND TS=(HIV OR "acquired immunodeficiency syndrome") AND TI=(measure OR measures OR scale OR scales OR tool OR tools)) AND LANGUAGE: (English)

Refined by: RESEARCH AREAS: ( PSYCHOLOGY OR HEALTH CARE SCIENCES SERVICES )

Indexes=SCI-EXPANDED, SSCI, BKCI-S, BKCI-SSH, ESCI Timespan=2008-2021

OR

Interventions (omitted conference proceedings, added Research Areas, limited ‘RCT’ terms to topic field)

Yield: 76

(TS=(stigma OR discrim*) AND TS=(HIV OR "acquired immunodeficiency syndrome") AND TS=("randomized controlled trial" OR "randomized controlled trials" OR RCT OR RCTs OR "intervention study" OR "intervention studies")) AND LANGUAGE: (English)

Refined by: RESEARCH AREAS: (PSYCHOLOGY OR HEALTH CARE SCIENCES SERVICES)

Indexes=SCI-EXPANDED, SSCI, BKCI-S, BKCI-SSH, ESCI Timespan=2008-2021

OR

Date: 5/6/2021

Yield: 263

Publication Date Range: 2008-2021

Terms

Indexes=SCI-EXPANDED, SSCI, A&HCI, CPCI-S, CPCI-SSH, BKCI-S, BKCI-SSH, ESCI, CCR-EXPANDED

Search Strategy

TOPIC: (stigma OR discriminat*) AND TOPIC: ("hiv" OR "acquired immunodeficiency syndrome" OR "AIDS") AND TOPIC: ("systematic review")

Campbell Collaboration

Date: 1/19/2021

Yield: 81

Campbell Systematic Reviews

Terms

stigma* OR discriminat*" and "HIV OR AIDS OR "acquired immunodeficiency"

Research evidence by topic

(stigma OR discrimination)

(HIV OR AIDS)

Cochrane Database of Systematic Reviews

Date: 1/29/2021

Yield: 6

Terms

(stigma OR discrimination):ti,ab,kw (Word variations have been searched)

AND

MeSH descriptor: [HIV] explode all trees OR MeSH descriptor: [Acquired Immunodeficiency Syndrome] 5 tree(s) exploded OR HIV OR "acquired immunodeficiency syndrome" OR "acquired immunodeficiency syndrome"):ti,ab,kw (Word variations have been searched)

PROSPERO

Date: 1/19/2021

Yield: 173

Terms

(stigma or discrimin*)

AND

MeSH DESCRIPTOR HIV EXPLODE ALL TREES OR MeSH DESCRIPTOR Acquired Immunodeficiency Syndrome EXPLODE ALL OR "acquired immunodeficiency syndrome" OR "acquired immunodeficiency" OR HIV

Open Science Framework

Date: 1/27/2021

Yield: 4 relevant projects

Terms: (stigma OR discrimination) AND (HIV OR AIDS)

Universal Human Rights Index

Date: 1/27/2021

Platform: https://www.ohchr.org/EN/Issues/HIV/Pages/Documents.aspx

Yield: 2 relevant documents

Terms: “Persons living with HIV/AIDS” AND (stigma OR discrimination)

PAIS

Date: 4/9/2021

Yield: 9

Terms: NOFT(hive and stigma and law and intervention)

International AIDS Society (IAS)

Date: 1/27/2021

Yield: 5 relevant documents

Terms: (stigma OR discrimination) AND (HIV OR AIDS)

Joint United Nations Programme on HIV/AIDS (UNAIDS)

Date: 1/27/2021

Yield: 15 relevant documents

Terms: (stigma OR discrimination) AND (HIV OR AIDS)

United Nations Development Programme (UNDP)

Date: 4/13/2021

Yield: 6 relevant documents

Terms: (stigma OR discrimination) AND (HIV OR AIDS)

STRIVE:

Date: 1/27/2021

Yield: 2 relevant documents

Terms: (stigma OR discrimination) AND (HIV OR AIDS)

Health Policy Plus

Date: 1/27/2021

Yield: 5 relevant documents

Terms: (stigma OR discrimination) AND (HIV OR AIDS)

The Center for HIV Law and Policy

https://www.hivlawandpolicy.org/

Date: 4/19/2021

Yield: 15 relevant documents

Terms: topic: “stigma”

The Global Fund

https://www.theglobalfund.org/en/funding-model/throughout-the-cycle/community-rights-gender/

Date: 4/13/2021

Yield: 20 relevant documents

Terms: “Human rights”

HIV Legal Network

http://www.hivlegalnetwork.ca/site/?lang=en

Date: 4/16/2021

Publications/Find Publications/ keyword search:

Yield: 11 relevant documents

Terms: stigma

Sage: A resource-sharing community for Canadian HIV and hepatitis C service providers

http://sagecollection.ca/en

Limited publication type: Reports

Date: 4/16/2021

Yield: 3 potentially relevant documents

Terms: STIGMA AND (LAW OR LEGAL)

HeinOnline

Date: 3/19/2021

Yield: 272

Terms

(stigma OR discrmin*) AND (HIV OR "acquired immunodeficiency syndrome" ) AND (framework OR frameworks OR "logic model" OR "logic models" OR "analytic model" OR "analytic models") Topic: AIDS AND Timespan: 2008-2021

OR (stigma OR discrmin*) AND (HIV OR "acquired immunodeficiency syndrome" ) AND (measure OR measures OR scale OR scales OR asses* OR tool OR tools) AND Topic: AIDS AND Timespan: 2008-2021

OR

(stigma OR discrmin*) AND (HIV OR "acquired immunodeficiency syndrome" ) AND ("randomized controlled trial" OR "randomized controlled trials" OR "RCT" OR "RCTs") Topic: AIDS AND Timespan: 2008-2021

OR

(stigma OR discrmin*) AND (HIV OR "acquired immunodeficiency syndrome" ) AND ("systematic review" OR "systematic reviews") Topic: AIDS AND Timespan: 2008-2021

LegalTrac

Date: 5/12/2021

Yield: 78

Terms

keyword search - ("acquired immunodeficiency syndrome" OR HIV) AND (stigma OR discriminat*)

AND

Publication Date Range: 2008 - present

AND

Document Type: Article

## Eligibility criteria

We used a PICOTS (participant, independent variable, comparator or study design, outcome/measure, timing, and setting) framework to structure the eligibility criteria:

- Participants: People living with or perceived to be living with HIV and people from groups disproportionately affected by HIV infection. We excluded studies of mixed populations and other participant targets unless the study provides HIV-relevant subgroup analyses.
- Intervention: Eligible intervention evaluations had to evaluate strategies and policies aimed at preventing, reducing, or mitigating HIV-related stigma and discrimination. Intervention evaluations aimed at internalized stigma, stigma and discrimination in healthcare, and stigma and discrimination at the legal or policy level were eligible. We excluded interventions assessing the effect of access to HIV care alone (e.g., antiretroviral therapy), studies evaluating a reorganization of care delivery without focus on healthcare providers (e.g., integrating HIV treatment into primary care), and studies exclusively addressing community and cultural stigma.
- Comparator / study design: Evaluations of *internalized stigma interventions* had to report on a concurrent or historic comparator (e.g., randomized controlled trial [RCT]) to be eligible. Evaluations of *healthcare interventions* were restricted to RCTs and controlled trials, as well as large observational studies targeting healthcare delivery organizations; we excluded non-RCTs targeting convenience samples of individual participants rather than organizations. For studies evaluating *the impacts of laws*, no comparator was required if the study author demonstrated an alternative analysis of determining the effect of the law. We included reports published by organizations in the public domain; we excluded student theses.
- Outcome: Intervention research had to provide a structured evaluation of the intervention and report the effects on an indicator of stigma or operationalization of discrimination to be eligible. Evaluations of legal changes or policies had to report empirical data from affected stakeholders.
- Timing: For interventions, only those published from 2008 on were included, building on the first People Living with HIV Stigma Index published in 2008, which transformed thinking around HIV-related stigma measurement [1].
- Setting: The review was not restricted regarding setting even as we restricted to English language publications.

## Critical appraisal

For *selection bias*, we assessed the allocation mechanism to intervention groups, baseline differences and potential confounders. *Performance bias* evaluated whether knowledge of the intervention allocation or circumstances such as the study context may have affected the outcome, and whether any deviations from intended interventions were balanced between groups. *Attrition bias* considered the number of dropouts, any imbalances across study arms, and whether missing values may have affected the reported outcomes. *Detection bias* assessed whether outcome assessors were aware of the intervention allocation, whether this knowledge could have influenced the outcome measurement, and whether the outcome ascertainment could differ between study arms. *Reporting bias* assessment included an evaluation of whether a pre-specified analysis plan was described (e.g., a published protocol), whether the numerical results likely had been selected on the basis of the results, and whether key outcomes were not or inadequately reported.

## Intervention Categorization Systems

The following systems were used for categorization of the type of interventions in the three different settings:

Internalized stigma

- Individual level only (e.g. health education, behavior change, stigma coping, and psychological support)
- Individual and relational levels (individual level combined with community-wide stigma awareness and reduction campaigns or and cognitive-behavioral therapy for partners of women living with HIV; interventions had to target existing relationships)
- Individual and structural levels (individual level combined with social or economic empowerment approaches)
- Structural level only (social or economic empowerment approaches, including ART roll-out and economic strengthening through saving schemes, housing, nutrition support, and income-generating)

Stigma and discrimination in healthcare settings

- Information-based (written and verbal information to understand HIV and stigma); alone or in combination
- Skills building (demonstrations and roll play); alone or in combination
- Structural (availing supplies, operating procedures); alone or in combination
- Contact strategies (testimonials and interaction); alone or in combination
- Biomedical interventions (access to care, HIV counseling and testing); alone or in combination
- Counseling and support (help cope with secondary stigma); alone or in combination

Stigma and discrimination in law and policy

- Legal advocacy
- Legal empowerment
- Discriminatory law or policy
- Court decision
- Policy change
- Legal change

## Component analysis

We hypothesized that:

- Community: Community participation in intervention design and implementation is a critical component of successful interventions to address HIV-related stigma and discrimination.
- Conceptual framework: Interventions that explicitly adopt a conceptual framework would be more effective given their strong conceptual foundation.
- Cooccurring stigma: Addressing intersectionality may support success of the intervention.
- Counseling: The provision of counselling and/or psychosocial support is an important feature of successful interventions to address HIV-related stigma and discrimination.
- Duty-bearers: It is important to understand the importance of working with duty-bearers to reduce stigma and discrimination in their actions.
- Education: The provision of information and/or education as well as skills-building are important features of successful interventions to reduce HIV-related stigma and discrimination.
- HIV medical specialist: Participation of an HIV medical specialist in the intervention would help reduce stigma and discrimination.
- Internalized focus: Interventions that explicitly addressed stigma would have a greater impact on internalized stigma than interventions where this was not a stated objective of the intervention.
- People living with HIV: Involvement of people openly living with HIV in the implementation of intervention activities is a critical success factor.
- Referral: When interventions provide referrals to other health and support services, they may be more effective in reducing HIV-related stigma and discrimination by connecting people to other sources of help and support.
- Rights-holders: It is important to work with rights-holders to help them claim their rights, seek appropriate services and develop ways to cope with HIV and/or related stigma and discrimination.
- Support person: Social support, including family support, plays an important role in addressing internalized HIV-related stigma.
- Total facility approach: It is important to work with everyone who works in a health facility in order to change the organizational culture around HIV-related stigma and discrimination.

Component combination analysis

For this, we reviewed components across the successful interventions to determine which configurations of components are associated with success [2]. We explored differences in the component structure between interventions that were determined to be successful and those that were determined to be unsuccessful. We analyzed the data as a crisp set with dichotomized presence or absence of components and intervention success. Categorizing evaluations as successful or unsuccessful followed a transparent algorithm. To reduce errors and bias, two independent literature reviewers blind to the intervention description or other study details determined success. We assessed the statistically significant reduction of stigma or discrimination compared to a control group (in the absence of a concurrent control group, the change compared to the pre-intervention status was used to determine intervention success).

Each component was analyzed as a potentially necessary condition, combined with other components through Boolean operators. We evaluated the parameters consistency (incl) and raw coverage (covS) for individual combinations as well as across all combinations identified in the dataset. A minimization analysis identified the simplest possible combination associated with success. Venn diagrams were used to visualize all possible and empirically tested combinations. Analyses used the R packages metafor, QCA, and gbm.

## Risk of Bias Table

| **Author, year** | **Selection bias** | **Performance bias** | **Attrition bias** | **Detection bias** | **Reporting bias** |
| --- | --- | --- | --- | --- | --- |
| Adia, 2020 [3] | High risk | Moderate/Unclear | Moderate/Unclear | Moderate/Unclear | Moderate/Unclear |
| Arora, 2014 [4] | Low risk | High risk | Moderate/Unclear | High risk | Moderate/Unclear |
| Barroso, 2014 [5] | Low risk | Low risk | Low risk | Low risk | Moderate/Unclear |
| Batchelder, 2020 [6] | High risk | High risk | High risk | High risk | High risk |
| Bauermeister, 2019 [7] | High risk | High risk | Moderate/Unclear | High risk | Moderate/Unclear |
| Bhatta, 2017 [8] | Low risk | Moderate/Unclear | Moderate/Unclear | High risk | Moderate/Unclear |
| Bogart, 2021 [9] | Moderate/Unclear | Moderate/Unclear | Low risk | High risk | Low risk |
| Brito, 2015 [10] | High risk | Moderate/Unclear | Moderate/Unclear | Moderate/Unclear | High risk |
| Chidrawi, 2016 [11] | Moderate/Unclear | High risk | Moderate/Unclear | High risk | Moderate/Unclear |
| Denison, 2020 [12] | Low risk | Moderate/Unclear | High risk | High risk | Moderate/Unclear |
| Denison, 2021 [13] | Moderate/Unclear | High risk | Moderate/Unclear | High risk | Low risk |
| France, 2019 [14] | Moderate/Unclear | High risk | Low risk | High risk | Moderate/Unclear |
| Gallently, 2012 [15] | High risk | High risk | Moderate/Unclear | Moderate/Unclear | Moderate/Unclear |
| Go, 2015 [16] | Low risk | Moderate/Unclear | Low risk | High risk | Moderate/Unclear |
| Gruskin, 2013 [17] | Moderate/Unclear | Low risk | Moderate/Unclear | Moderate/Unclear | Moderate/Unclear |
| Harper, 2014 [18] | High risk | High risk | Low risk | High risk | Moderate/Unclear |
| Hickey, 2021 [19] | Moderate/Unclear | Moderate/Unclear | Low risk | High risk | Low risk |
| Kaponda, 2009 [20] | High risk | High risk | Moderate/Unclear | High risk | Moderate/Unclear |
| Lane, 2011 [21] | High risk | Moderate/Unclear | Moderate/Unclear | Moderate/Unclear | Low risk |
| Li, 2013 [22] | Low risk | Low risk | Low risk | High risk | Low risk |
| Lifson, 2017 [23] | Moderate/Unclear | High risk | Low risk | High risk | Moderate/Unclear |
| Lohiniva, 2016 [24] | High risk | High risk | High risk | High risk | Low risk |
| Lowther, 2018 [25] | Low risk | Low risk | Moderate/Unclear | Moderate/Unclear | Moderate/Unclear |
| Mahajan, 2016 [26] | Low risk | Low risk | Moderate/Unclear | Moderate/Unclear | Moderate/Unclear |
| Mak, 2015 [27] | Low risk | Low risk | Low risk | Low risk | Moderate/Unclear |
| Maluccia, 2017 [28] | Moderate/Unclear | Moderate/Unclear | High risk | High risk | Low risk |
| Masquillier, 2015 [29] | Low risk | Moderate/Unclear | Low risk | High risk | Moderate/Unclear |
| Mbela, 2011 [30] | Low risk | Low risk | Low risk | High risk | Low risk |
| Miller, 2021 [31] | High risk | High risk | Moderate/Unclear | Moderate/Unclear | Low risk |
| Mockiene, 2011 [32] | Low risk | High risk | High risk | High risk | Low risk |
| Nestadt, 2019 [33] | Low risk | Moderate/Unclear | Low risk | High risk | Moderate/Unclear |
| Norr, 2012 [34] | Moderate/Unclear | Low risk | High risk | High risk | Moderate/Unclear |
| Nyamathi, 2013 [35] | Moderate/Unclear | Low risk | Low risk | Low risk | Moderate/Unclear |
| Nyblade, 2018 [36] | Moderate/Unclear | High risk | Moderate/Unclear | High risk | Moderate/Unclear |
| Nyblade, 2020 [37] | Moderate/Unclear | Low risk | Low risk | Moderate/Unclear | Moderate/Unclear |
| Odeny, 2013 [38] | High risk | High risk | High risk | High risk | High risk |
| Onyemelukwe, 2017 [39] | High risk | Moderate/Unclear | Moderate/Unclear | Moderate/Unclear | Moderate/Unclear |
| Peltzer, 2018 [40] | Low risk | Low risk | High risk | Low risk | Moderate/Unclear |
| Petersen, 2014 [41] | Low risk | Low risk | High risk | Low risk | High risk |
| Prinsloo, 2017 [42] | High risk | High risk | Moderate/Unclear | High risk | Low risk |
| Pulerwitz, 2015 [43] | Moderate/Unclear | Low risk | High risk | Low risk | Moderate/Unclear |
| Rao, 2012 [44] | Moderate/Unclear | High risk | Low risk | Low risk | Moderate/Unclear |
| Rao, 2018 [45] | Low risk | Low risk | High risk | Low risk | Moderate/Unclear |
| Rongkavilit, 2015 [46] | Low risk | Low risk | High risk | Low risk | High risk |
| Schwartz, 2015 [47] | Moderate/Unclear | Moderate/Unclear | High risk | High risk | Moderate/Unclear |
| Sears, 2013 [48] | High risk | Moderate/Unclear | Low risk | Low risk | Moderate/Unclear |
| Shamsaei, 2020 [49] | Moderate/Unclear | High risk | Low risk | High risk | High risk |
| Singh, 2020 [50] | Moderate/Unclear | Low risk | High risk | High risk | Low risk |
| Siraprapasiri, 2020 [51] | Moderate/Unclear | High risk | High risk | High risk | High risk |
| Skinta, 2015 [52] | High risk | High risk | High risk | High risk | Low risk |
| Sommerland, 2020 [53] | Moderate/Unclear | Moderate/Unclear | High risk | Moderate/Unclear | Low risk |
| Sourcebook, 2020 [54] | High risk | High risk | Moderate/Unclear | Moderate/Unclear | Low risk |
| Srinivasan, 2021 [55] | Low risk | Moderate/Unclear | Moderate/Unclear | High risk | Moderate/Unclear |
| Step, 2020 [56] | Moderate/Unclear | High risk | Moderate/Unclear | High risk | Moderate/Unclear |
| Tsai, 2013 [57] | High risk | High risk | Low risk | High risk | Moderate/Unclear |
| Tsai, 2017 [58] | Moderate/Unclear | Moderate/Unclear | High risk | High risk | High risk |
| Tshabalala, 2011 [59] | Moderate/Unclear | Moderate/Unclear | Low risk | High risk | Low risk |
| UNAIDS, 2021 [60] | High risk | Moderate/Unclear | Moderate/Unclear | Moderate/Unclear | Moderate/Unclear |
| UNDP, 2016 [61] | High risk | Moderate/Unclear | Moderate/Unclear | Moderate/Unclear | Moderate/Unclear |
| UNDP, 2019 [62] | High risk | Moderate/Unclear | Moderate/Unclear | Moderate/Unclear | Low risk |
| UNDP, 2021 [63] | High risk | Moderate/Unclear | Moderate/Unclear | Moderate/Unclear | Low risk |
| Uys, 2009 [64] | Moderate/Unclear | High risk | Low risk | High risk | Low risk |
| Van Tam, 2012 [65] | Low risk | Moderate/Unclear | High risk | High risk | Moderate/Unclear |
| Varas-Diaz, 2013 [66] | Low risk | Low risk | High risk | Low risk | Moderate/Unclear |
| Wagner, 2012 [67] | High risk | Low risk | Low risk | High risk | Moderate/Unclear |
| Watt, 2011 [68] | High risk | High risk | High risk | High risk | Moderate/Unclear |
| Watt, 2020 [69] | Low risk | Moderate/Unclear | Low risk | High risk | Moderate/Unclear |
| Wu, 2008 [70] | Low risk | High risk | Moderate/Unclear | High risk | Moderate/Unclear |
| Yigit, 2020 [71] | Low risk | Moderate/Unclear | Low risk | High risk | Low risk |
| Yiu, 2010 [72] | Low risk | Low risk | Low risk | Low risk | Moderate/Unclear |

## Evidence table: Internalized Stigma Interventions

| **Author, year**  **Country**  **Study design**  **Sample size**  **Domain** | **Participant details**  **Cooccurring stigmas**  **Context/setting**  **Study objective**  **Definitions** | **Intervention**  **Duration**  **Comparator**  **Length of follow up** | **Stigma results**  **Alternative results**  **Unintended consequences** |
| --- | --- | --- | --- |
| Barroso, 2014 [5]  Relf, 2015 [73]; Relf, 2015 [74]  **Country:** USA  **Study design:** RCT  **Sample size:** N = 100  **Domain:** Internalized stigma | **Participant details:** Women living with HIV or AIDS, at least 18 years of age, able to communicate in English and were mentally competent to provide informed consent  **Cooccurring stigmas:** NA  **Context / setting:** Healthcare  **Study objective:** To compare outcomes across time in HIV-positive women living in the Deep South who received a stigma reduction intervention  **Implementer:** Academic researchers  **Definitions:**  HIV-related stigma = prejudice, discounting, discrediting, and discrimination directed at people perceived to have AIDS or HIV  Internalized stigma = having negative feelings and beliefs associated with HIV and applying them to the self  Anticipated stigma = expectations of discrimination, stereotyping, and/or prejudice from others in the future because of one’s HIV  Enacted Stigma = experiences of discrimination, stereotyping, and/or prejudice from others in the past or present because of one’s HIV | **Intervention**  Women in the intervention arm received an iPod Touch with the video loaded on it as an MP4 file (45-min video ‘‘Maybe Someday: Voices of HIV-Positive Women’);’ characters in the video are designed to connect with viewers on multiple levels and acknowledge the interplay, connections, and potential disconnections between their HIV status and other aspects of their lives; video shows experience of being an HIV-positive women, fear of the negative social effects that come with telling other people about one’s HIV status, women’s struggle about whether or not to tell their children, importance of communicating with nurses, doctors, and those family members and friends whom they trust; positive effects of disclosure; extra stigma and discrimination connected with being a woman, being a minority woman, and being a mother; and ambivalence about disclosing one’s HIV status to potential and actual sex partners  **Duration (months):** 3 months total; 45 minute video watched at least weekly for 1 month, then as much or as little as desired for the remaining 2 months  **Comparator**  Control group received an iPod Touch with nothing loaded on it  **Length of follow up:** 3 months | **Stigma results:** There was a treatment-by-time effect for stigma scores (Internalized HIV Stigma Scale; p=0.0060) with both treatment groups showing a significantly greater linear decrease in scores over time relative to the control arm.  **Alternative results:**  **Unintended consequences:** NA |
| Batchelder, 2020 [6]  **Country:** USA  **Study design:** Pre-post  **Sample size:** N = 10  **Domain:** Internalized stigma | **Participant details:** 18 years of age or older, HIV-positive, able to complete informed consent, consent to release their medical records, and own a cell phone with text message capacity, and experienced moderate substance use in the last three months for two or more substances including alcohol, excluding cannabis and tobacco  **Cooccurring stigmas:** Substance use disorder  **Context / setting:** Healthcare  **Study objective:** To improve emotion regulation related to internalized stigma and shame, differing from existing interventions addressing substance use or depression as barriers to HIV-care  **Implementer:** Academic researchers  **Definitions:**  Stigma = self-conscious emotions, such as shame, are considered social emotions as they result from the appraisal of how an experience pertains to one’s self in relation to others | **Intervention**  Each participant attended 5 in-person, individual intervention sessions with an interventionist that lasted 60 minutes for 5 weeks; participants received two emotion query text messages daily and for about two months after the last session; by the end of each session, each participant developed compassionate self-statements based on the session content; session content included metacognitive awareness of emotions and cognitions, cognitive reframing (compassionate self-statements), and identifying and refining self-care goals (goal setting, problem-solving); in the final session, participants selected the compassionate self-statements they felt would be most helpful when experiencing each of the self-conscious emotion response options from the daily text queries and for 2 months following the sessions received by text their chosen statements to model using compassionate self-reframing when experiencing self-conscious emotions  **Duration (months):** 3.25 months total; 5 weekly sessions (60 minutes) and daily emotion query text messages which followed for 2 months after completion of sessions  **Comparator**  Baseline  **Length of follow up:** 3.25 months | **Stigma results:** Participants self-reported statistically significant less self-judgment post-intervention consistent with a reduction in internalized stigma (HIV and Abuse Related Shame (HARSI))  **Alternative results:**  **Unintended consequences:** From participants self-reports, the mean adherence to antiretroviral therapy decreased from 94% (SD 5.3) to 89% (SD 14.3) after the intervention. |
| Bauermeister, 2019 [7]  Hightow-Weidman, 2017 [75]  **Country:** USA  **Study design:** Pre-post  **Sample size:** N = 238  **Domain:** Internalized stigma | **Participant details:** Identify as male sex assigned at birth, be between 18 and 30 years old (inclusive), identify as African American/Black, reside in North Carolina, and have access to a mobile device with internet connectivity who were either HIV-negative, HIV-positive, or HIV status unknown  **Cooccurring stigmas:** Sexual orientation  **Context / setting:** Other : Mobile/Internet  **Study objective:** To seek to characterize whether sexuality- and HIV-related stigma scores change over time  **Implementer:** Academic researchers  **Definitions:**  Anticipated stigma = expression of fear of, concern about, or expectation of future stigma or discrimination from an action, identity, characteristic, or behavior  Experienced stigma = stigmatizing or discriminatory events and experiences  Internalized stigma = acceptance or endorsement of negative societal attitudes or language around HIV/sexual minority status as part of one's own values, beliefs, or language | **Intervention**  Intervention arm participants had access to 3 spaces where they could share and receive information and experiences and garner social support; the Forum was a space where participants could initiate or contribute to conversations within various topic areas (e.g. Getting Tested, Safer Sex, Dating & Relationships, Healthy Living, Fashion & Entertainment, Life Skills, Current Events); in the Ask Dr. W section of the site, participants could post anonymous questions to a board-certified infectious disease doctor; the question and response were then posted (within 48 h) for all participants to view; in the Getting Real section participants could share and comment on multimedia content that they created themselves (e.g. poetry, videos, images, reflections) or that they linked to on the web (e.g. news stories, YouTube videos)  **Duration (months):** 3 months  **Comparator**  Baseline  **Length of follow up:** 6 months | **Stigma results:** 4.6% made internalized HIV stigma contributions to the forum.  Steward's 10 item sub scale showed a reduction in perceived HIV stigma (p ≤ 0.05); forum posts indicating anticipated HIV stigma reported increases in HIV stigma over time (p ≤ 0.01).  **Alternative results:**  **Unintended consequences:** NA |
| Bhatta, 2017 [8]  **Country:** Nepal  **Study design:** RCT  **Sample size:** N = 132  **Domain:** Internalized stigma | **Participant details:** HIV infected, aged 18 years or older, and have been receiving ART between 6 months and 2 years prior to the study as per the national ART guidelines of National Center for AIDS and STD Control  **Cooccurring stigmas:** NA  **Context / setting:** Community  **Study objective:** To assess the efficacy of a social self-value empowerment intervention package to improve quality of life  **Implementer:** Academic researchers  **Definitions:**  NA | **Intervention**  Culturally accepted and adopted components were developed after several consultations with experts and pre-tested among people living with HIV; the empowerment intervention focused on autonomy and community activism, self-esteem/self-efficacy, self-care, optimism, and control over the future, family and social relationships, power-powerlessness, management of stress and righteous anger, self-respect/self-worth, stigma and defeat with stigma, stigma and discrimination issues, legal provisions, and human and health rights  **Duration (months):** 6 weekly sessions  **Comparator**  All participants received routine standard care, including pre ART counseling, routine medical and laboratory tests and monthly follow up for ART; standard care is provided by government organizations and ART is dispensed free of charge  **Length of follow up:** 6 months | **Stigma results:** Stigma scores were reduced by half at 3 months in the intervention group with no further changes at 6 months (23-item scale; p<0.001).  **Alternative results:**  **Unintended consequences:** NA |
| Bogart, 2021 [9]  **Country:** United States  **Study design:** RCT  **Sample size:** N = 76  **Domain:** Internalized stigma | **Participant details:** Self-identify as hispanic or latino, at least 18 years old, verified HIV-positive, biologically male, ever had sex with another man, not currently taking antiretroviral therapy or taking antiretroviral therapy and reported missing at least 1 dose in the past month, or reported fewer than 2 HIV visits in the past 12 months  **Cooccurring stigmas:** Sexual orientation  **Context / setting:** Community  **Study objective:** To improve coping with intersectional stigma, address medical mistrust, and improve antiretroviral treatment adherence  **Implementer:** Civil Society (RAND and Fenway Institute) and Academic researchers  **Definitions:**  Internalized stigma = barrier to effective coping that could lead to non-adherence of ART | **Intervention**  The 8-session Spanish-language intervention had 7 sessions of content plus a “graduation” ceremony; the intervention included psychoeducation about discrimination, HIV disparities, and HIV treatment adherence, including defining discrimination to enable participants to be more aware in recognizing identity-based mistreatment  **Duration (months):** 2 months  **Comparator**  Wait-list control group were offered the intervention sessions after all data collection was completed for the pilot trial  **Length of follow up:** 7 months | **Stigma results:** Internalized HIV stigma (Internalized AIDS-Related Stigma Scale) showed small-to-medium, statistically significant effect sizes.  **Alternative results:**  **Unintended consequences:** NA |
| Chidrawi, 2016 [11]  **Country:** South Africa  **Study design:** Pre-post  **Sample size:** N =  **Domain:** Internalized stigma | **Participant details:** Over 18 years old, conversant in either Afrikaans, English or Setswana, HIV-positive for a minimum of six months, willing to give informed consent for participation and recording of discussions, and required to openly share in HIV-status disclosure workshops with other PLWH  **Cooccurring stigmas:**  **Context / setting:** Community  **Study objective:**  **Implementer:** Academic researchers  **Definitions:**  Stigma = deeply discrediting personal phenomenon  HIV stigma = manifests as the assertion of a socially constructed “undesired differentness” through acts of ostracism, discrimination, social control, marginalization and social domination  Internal stigma = self-stigmatizing by people living with HIV or their insider view  Received stigma = relates to concepts like perceived/anticipated, outsider-view/external or felt stigma and as such experienced by people living with HIV as directed towards them  Associated stigma or secondary stigma = relates to stigma that stems from someone's association with people living with HIV and often includes people living close to people living with HIV | **Intervention**  Sharing of information on HIV stigma and coping with HIV stigma, equalizing relationships between PLWH and providers through increased interaction and contact among them by grouping them together, and empowerment of members of both groups towards leadership in HIV stigma reduction through practical knowledge and experience of project planning regarding HIV stigma reduction and implementation in their communities  **Duration (months):** 5  **Comparator**  Baseline  **Length of follow up:** 12 months | **Stigma results:** Perceived AIDS Stigma Instrument PLWA (HASI-P) and the AIDS-Related Stigma Measure for Community HIV Stigma found that stigma experiences were reduced following the community-based stigma reduction intervention (p < 0.01).  **Alternative results:**  **Unintended consequences:** |
| Denison, 2020 [12]  Denison, 2020 [76]  **Country:** Zambia  **Study design:** RCT NCT04115813  **Sample size:** N = 276  **Domain:** Internalized stigma | **Participant details:** Aged between 15-24, were aware of their HIV status, had been on ART for at least 6 months, and spoke Bemba or English  **Cooccurring stigmas:** NA  **Context / setting:** Healthcare  **Study objective:** To test a peer mentoring program among HIV positive youths  **Implementer:** NGO and academic researchers  **Definitions:**  NA | **Intervention**  All participants received an orientation meeting with a health care provider, their assigned youth peer mentor, and an adult caregiver (if invited by the youth participant); following the orientation meeting, intervention arm participants continued to meet with their assigned mentor for one-on-one meetings once per month over six months; participants were also invited to monthly youth group meetings, the first of which was required in order to encourage those youth to try a group format; simultaneously, the adult caregivers were offered 3 caregiver group meetings, held every other month, designed to provide enhanced knowledge and skills to support their youth living with HIV; the project also provided Youth and Caregiver Journals that participants could use to track adherence, engage with educational topics, and reflect on their journeys; after the midline data collection, intervention arm participants started a maintenance phase  **Duration (months):** 6 months  **Comparator**  Participants in the comparison arm received the standard of care for adolescents and young adults as offered at the HIV clinics, including regular clinic visits and the option of joining monthly youth group meetings; after the midline data collection, comparison arm participants started the Project YES! intervention, including transitioning eligible youth from the children’s hospital to the HIV clinic in the adult hospital  **Length of follow up:** 6 months | **Stigma results:** Internalized stigma was measured using three questions from the Internalized AIDS Stigma Scale, and, after conducting a GEE model to evaluate the intervention's effect on stigma, results showed positive effects on internalized stigma (OR 0.39, CI 0.21, 0.73).  **Alternative results:**  **Unintended consequences:** NA |
| Denison, 2021 [13]  **Country:** Zambia  **Study design:** RCT NCT04442399  **Sample size:** N = 100  **Domain:** Internalized stigma | **Participant details:** Adolescents between 15 and 19 years old, aware of HIV status, and had been on antiretroviral therapy for at least 6 months, caregivers at least 20 years old, knew the adolescent took antiretroviral therapy, and helped adolescent with their HIV care, both needed to live within a 30-minute drive from study clinic, had no plans of moving from area in within 6 months, willing to attend 10 group sessions over 5 months, and able to speak Bemba or English  **Cooccurring stigmas:** NA  **Context / setting:** Healthcare  **Study objective:** To determine if adolescents would invite, and caregivers would attend, group sessions twice a month over 5–6 months  **Implementer:** Academic researchers and NGO  **Definitions:**  Internalized stigma = feeling of worthlessness, shame or guilt because they are HIV-positive | **Intervention**  The intervention was based on the WHO-endorsed manual entitled Positive Connections: Leading Information and Support Groups for Adolescents Living with HIV, designed for adults to lead information and support groups for ALHIV in resource-poor settings. Healthcare providers at each clinic underwent a weeklong training and facilitated the 10 sessions held every other Saturday at their HIV clinic over a six-month period; the pair arrived together and attended separate group sessions, sometimes participating in join activities; after each group session, participants completed a post-session survey using audio computer-assisted self-interview software  **Duration (months):** 6 months total, 10 two hour sessions  **Comparator**  Standard of care was offered, which included regular clinic visits and the option of joining existing monthly youth group meetings; adolescent met with study staff monthly to collect their medication electronic monitoring system adherence data, caregivers completed baseline and enplane surveys only  **Length of follow up:** 6 months | **Stigma results:** Internalized stigma (3 questions from the Internalized AIDS Stigma Scale (IA-RSS)) found a greater reduction in feelings of shame (p=0.07) and worthlessness (p=0.06) in the intervention group compared to the comparison group.  **Alternative results:**  **Unintended consequences:** NA |
| France, 2019 [14]  **Country:** Zimbabwe  **Study design:** Pre-post  **Sample size:** N = 23  **Domain:** Internalized stigma | **Participant details:** People living with HIV were recruited from a local HIV support network via open call for volunteers; all were members of Harare-based support groups which were part of the ZNNP+ network  **Cooccurring stigmas:** NA  **Context / setting:** Community  **Study objective:** To assess the impact of peer support for people living with HIV  **Implementer:** Academic researchers  **Definitions:**  Self-stigma = negative self-judgements, which at times may result in feelings of shame, worthlessness and self-blame | **Intervention**  A faciltated programme incorporating Inquiry-Based Stress Reduction: the Work of Byron Katie, which is a guided form of self-inquiry which helps users to overcome negative thoughts and beliefs designed to support participants to question and work through self-stigmatizing beliefs; technical support and curriculum development was carried out by International Certified Facilitators, coordinated by The Work for Change organization; participants took part in 12 four hour group sessions run over 12 weeks using peer and mentor methods, a weekly one-hour individual session with a facilitator, as well as homework  **Duration (months):** 3 months  **Comparator**  Baseline  **Length of follow up:** 3 months | **Stigma results:** Participants reported significant improvements in factors including self-stigma (Internalized AIDS Stigma Scale, 1-month follow-up vs baseline p = 0.039; 3-month follow-up vs baseline p = 0.003, n = 23, Wilcoxon Matched Pairs Signed Rank Test). Qualitatively, participants reported improvements including lessened fears around disclosure of their HIV status, reduced feelings of life limitations due to HIV, and greater positive mentality. Improvements persisted at three-month follow-up.  **Alternative results:** Participants reported significant improvements in depression (1mo vs baseline Z = 3.7, p = <0.001; 3mo vs baseline Z = 3.3, p = 0.001).  **Unintended consequences:** NA |
| Go, 2015 [16]  **Country:** Vietnam  **Study design:** RCT  **Sample size:** N = 455  **Domain:** Internalized stigma | **Participant details:** HIV-positive male adults, able and willing to bring in an injecting network member for screening, had sex in the past 6 months, and injected drugs in the past 6 months  **Cooccurring stigmas:** Injection drug use stigma  **Context / setting:** Community  **Study objective:** To develop and evaluate an behavioral intervention for people who inject drugs  **Implementer:** Academic researchers  **Definitions:**  NA | **Intervention**  Structural-level community stigma reduction program combined with individual-level counseling and skill-building support groups; the intervention draws on theories of social action, social identify, and diffusion of innovation; social action theory, which centers on the interaction between internal affective states (e.g., internalized stigma and avoidance coping), the social environmental context (e.g., peer networks, sexual partners, community) and self-regulation capabilities (e.g., mastery of technical, social and problem solving skills), guided intervention content; 2 individual counseling sessions, 2 small group education sessions, and an optional dyad session with a significant other  **Duration (months):** Standard of care  **Comparator**  Standard of care HIV testing and counseling  **Length of follow up:** 24 months | **Stigma results:** Average HIV stigma scores (perceived and experienced) remained stable over the follow-up and comparable across arms.  **Alternative results:**  **Unintended consequences:** NA |
| Harper, 2014 [18]  Hosek, 2011 [77]  **Country:** USA  **Study design:** Pre-post  **Sample size:** N = 50  **Domain:** Internalized stigma | **Participant details:** Participants were between the ages of 16 and 24, diagnosed with HIV, aware of the diagnosis for at least 15 months, and received services at one of the selected clinical sites were approached by the study staff  **Cooccurring stigmas:** NA  **Context / setting:** Healthcare  **Study objective:** To describe a group-based intervention for adolescents and young adults newly diagnosed with HIV  **Implementer:** Academic researchers  **Definitions:**  Stigma = a type of spoiled identity that occurs when a person or group possesses a particular attribute that is viewed by others in society as an undesirable difference resulting in social sanctions against those who possess that attribute  HIV-related stigma = socially shared attitudes, beliefs, or actions that promote and perpetuate the devalued status of people living with or affected by HIV | **Intervention**  Project ACCEPT is a group-based behavioral intervention based on the disability-stress-coping model; participants took part in 3 individual session and 9 gender-specific weekly group sessions addressing HIV information, coping skills, and group sessions provide contact with other youth who are living with HIV to improve social support  **Duration (months):** 9 group sessions, 3 individual sessions, and 9 two hour individual modules  **Comparator**  Baseline  **Length of follow up:** 3 months | **Stigma results:** Results from the combined sample (males and females) revealed overall reductions in stigma (Berger scale) in three dimensions: personalized stigma, disclosure concerns, and negative self-image, although only the combined-sample effects for negative self-image were maintained at 3-month follow-up.  **Alternative results:**  **Unintended consequences:** NA |
| Hickey, 2021 [19]  **Country:** Kenya  **Study design:** RCT NCT02474992  **Sample size:** N = 304  **Domain:** Internalized stigma | **Participant details:** Participants were over 18 years, missed a clinic visit by more than 3 days during study enrollment  **Cooccurring stigmas:** Anticipated, enacted  **Context / setting:** Unclear  **Study objective:** To describe a trial of a targeted microclinic intervention at nine public clinics in rural western Kenya  **Implementer:** Academic researchers and NGO (Family AIDS CARE Education Services in Kenya)  **Definitions:** | **Intervention**  The microclinic intervention consisted of formation of a group of 5–10 close family, friends and other members of the participant’s social support system, irrespective of the HIV status of these individuals. Microclinic groups were assigned a CHW group facilitator and were guided through eight sessions scheduled every two weeks at a location of the group’s choosing. Microclinic curriculum topics for the eight sessions included 1) program overview and group confidentiality, 2) HIV local epidemiology and prevention, 3) HIV treatment basics, 4) group support for HIV medication adherence and engagement in care, 5) local beliefs about HIV, herbs and nutrition, 6) group support for combating stigma, 7) group HIV status disclosure, and 8) debriefing of group disclosure and group support moving forward.  **Duration (months):** 4 months total, 8 sessions every 2 weeks  **Comparator**  Guaranteed an opportunity to participate in a microclinic group and training program following completion of the study period  **Length of follow up:** 12 months | **Stigma results:** HIV-associated stigma scale internalized stigma scores were lower in the intervention arm (baseline adjusted difference -0.43, CI -0.62, -0.23; p<0.001).  **Alternative results:**  **Unintended consequences:** NA |
| Lifson, 2017 [23]  **Country:** Ethiopia  **Study design:** Pre-post  **Sample size:** N = 142  HIV clinics in Arba Minch Hospital and 5 local health centers  **Domain:** Internalized stigma | **Participant details:** Patients 18 years or older and newly enrolled in HIV clinical care within the previous 3 months at HIV clinics and local health centers  **Cooccurring stigmas:** NA  **Context / setting:** Healthcare,Other : Community health support workers provide a bridge to people living with HIV/AIDS  **Study objective:** To describe the impact of a peer support program on retention in HIV care  **Implementer:** Academic researcher and Government  **Definitions:**  NA | **Intervention**  Each participant was assigned one community health support worker with a caseload of 20 clients, visiting 1 to 4 times/month to provide education on HIV treatment, nutrition, and other health promoting behaviors; counseling and social support, discussing with clients negative personal feelings about being HIV-positive and disclosure of HIV status to others; facilitated communication with the HIV clinic nurse; and referrals as needed to community organizations for assistance with nutrition, clothing, housing, or income-generating activities; all community health support workers were people living with HIV/AIDS themselves  **Duration (months):** Client visits 1 to 4 times a month  **Comparator**  Baseline  **Length of follow up:** 12 months | **Stigma results:** The HIV/AIDS Stigma Instrument - PLWA (HASI-P) results showed that internalized stigma was reduced from 1.6 at baseline to 0.05 at 12 months (p < 0.001).  **Alternative results:**  **Unintended consequences:** NA |
| Lowther, 2018 [25]  Lowther, 2014 [78]; Lowther, 2012 [79]  **Country:** Kenya  **Study design:** RCT  **Sample size:** N = 30  **Domain:** Internalized stigma | **Participant details:** HIV positive patients who were at least 18 years old, on antiretroviral therapy for more than one month, reported moderate to severe pain or symptoms lasting at least 2 weeks  **Cooccurring stigmas:** NA  **Context / setting:** Healthcare  **Study objective:** To describe experiences of stigma and stigma resistance in a nurse-led palliative care intervention  **Implementer:** Academic Researchers  **Definitions:**  Stigma = mark of social disgrace, where the stigmatized are excluded from social acceptance and are socially devalued | **Intervention**  Patients randomly allocated to the intervention arm received clinical care from a nurse with 2 weeks of training in palliative care and ongoing clinical support and supervision from experienced palliative care providers; the nurse had the option to refer complex cases for management at a hospice; the training was provided by local expert palliative care sites, delivered in a 2-week period, tailored to the needs of patients living with HIV who are not necessarily in advanced stages and who are currently on anti-retroviral therapy; the training was designed using national and international guidelines with a specific focus on the management of common HIV symptoms  **Duration (months):** 0.5  **Comparator**  Received the usual clinical care delivered by the HIV clinic; nurses who have had no exposure to palliative care provided this service  **Length of follow up:** 5 months | **Stigma results:** Inductive thematic analysis revealed increased resistance to HIV-associated stigma in both the intervention and control groups. Intervention participants reported improved self-esteem, self image and acceptance, and helped participants resist internalized stigma.  **Alternative results:**  **Unintended consequences:** NA |
| Maluccia, 2017 [28]  Maluccio, 2015 [80]; Rawat, 2014 [81]  **Country:** Uganda  **Study design:** Cohort study  **Sample size:** N = 904  **Domain:** Internalized stigma | **Participant details:** HIV-positive, non-pregnant, above the age of 18, eligible for food assistance based on World food program's poverty assessment criteria but had not received any source in the previous 12 months, were antiretroviral therapy naive, had a CD4 count between 200 and 450  **Cooccurring stigmas:** NA  **Context / setting:** Community  **Study objective:** To examine the program impact of a food assistance intervention on stigma  **Implementer:** Government and NGO  **Definitions:**  Internalized stigma = thoughts and behaviors stemming from the person’s own negative perceptions about herself or himself based on her/his HIV status  External/enacted/received stigma = all types of stigmatizing behavior towards a person with HIV/AIDS experienced or described by people living with HIV/AIDS | **Intervention**  AIDS support organization partnering with World food program to deliver food assistance in the form of monthly food baskets for participants, treatment support (treatment and disclosure counselling)  **Duration (months):** 12 months  **Comparator**  Control did not receive World food program assistance  **Length of follow up:** 12 months | **Stigma results:** Significant effect on reported internalized stigma of approximately 0.2 standard deviations (p<0.01).  **Alternative results:**  **Unintended consequences:** NA |
| Masquillier, 2015 [29]  Wouters, 2014 [82]; Bhargava, 2018 [83]  **Country:** South Africa  **Study design:** RCT NCT00821366  **Sample size:** N = 294  **Domain:** Internalized stigma | **Participant details:** Participants at least 18 years of age, have commenced antiretroviral therapy in the previous 5 weeks, and resided in the town/village where the participating public antiretroviral therapy health facility was located  **Cooccurring stigmas:** NA  **Context / setting:** Healthcare  **Study objective:** To determine whether a peer support intervention had an impact on stigma  **Implementer:** Academic Researchers  **Definitions:**  External stigma = consists of negative attitudes expressed to people living with HIV/AIDS  Internal stigma = results from the internalization of AIDS stigmatizing beliefs | **Intervention**  Peer adherence support comprised bi-weekly visits by a trained community-based peer adherence supporter who at recruitment had been on ART for at least 12 months; recruited peer adherence supporters were provided with four days of theoretical and practical training on antiretroviral treatment and adherence support; peer adherence supporters performed a wide range of adherence counselling tasks, included asking the patients about their primary complaints, talking to them about these complaints, improving their knowledge about HIV/AIDS/ART, referring them to the facility if needed, motivating them to adhere to treatment, performing a weekly pill-count to assess adherence and, if needed, providing counselling in order to address possible barriers to medication compliance  **Duration (months):** 18 months  **Comparator**  Control group received standard care  **Length of follow up:** 12 months | **Stigma results:** Berger's HIV Stigma scale found a significant difference in average internalized stigma at follow-up 1 vs follow-up 2 after some participants partnered with a treatment buddy (p=0.042).  **Alternative results:**  **Unintended consequences:** Social side effects; exacerbating experienced levels of stigma |
| Nestadt, 2019 [33]  Mellins, 2014 [84]; Pardo, 2017 [85]  **Country:** Thailand  **Study design:** RCT  **Sample size:** N = 88  **Domain:** Internalized stigma | **Participant details:** Perinatal transmission of HIV child, 9–14 years old, aware of HIV+ status, and currently taking antiviral therapy  **Cooccurring stigmas:** NA  **Context / setting:** Community  **Study objective:** To evaluate the intervention CHAMP+ in Thailand  **Implementer:** Academic Researchers  **Definitions:**  NA | **Intervention**  The CHAMP model was adapted to address the unmet needs of early adolescents and caregivers; the model is grounded in a modified Social Action Theory, retained the focus on family strengthening, but also included HIV-related content such as medication adherence, feelings about HIV and identity, disclosure, and stigma  **Duration (months):** 6 months, 11 sessions  **Comparator**  The standard of care condition consisted of routine health education and practice offered to the child and families by clinic staff at regularly scheduled medical appointments,  including reviewing medications, as well as guidance regarding maintaining overall health and psychosocial support  **Length of follow up:** 9 months | **Stigma results:** Within the CHAMP+ group, at 6 months, statistically significant improvements were seen in caregiver-reported internalized and externalized HIV stigma (adapted from a measure originally developed for pediatric epilepsy-related stigma). At 9 months, within-group improvements were sustained.  **Alternative results:**  **Unintended consequences:** NA |
| Nyamathi, 2013 [35]  **Country:** India  **Study design:** RCT  **Sample size:** N = 68  2 villages  **Domain:** Internalized stigma | **Participant details:** Rural women, live with HIV/AIDS, be between the ages of 18-45, be on antiretroviral therapy for at least 3 months, had to have a CD4 cell count of over 100  **Cooccurring stigmas:** NA  **Context / setting:** Community  **Study objective:** To assess the impact of an intervention to reduce internalized stigma and avoidant coping in a sample of rural women living with HIV/AIDS in India  **Implementer:** Academic researchers  **Definitions:**  NA | **Intervention**  Six sessions included the following topics: HIV/AIDS and dealing with the illness; learning about anti-retroviral therapy and ways to overcome barriers; parenting and maintaining a healthy home environment; how to improve coping, reduce stigma and care for family members; basics of good nutrition and easy cooking tips; and benefits of engagement in a life skills class, such as computer skills, marketing, and embroidery; the primary role of the intervention Asha was to visit the four to five women living with HIV/AIDS assigned to them weekly for 15 – 60 minutes, monitor barriers to ART adherence, and provide assistance to mitigate any barriers they faced in accessing health care or the prescribed treatment; assistance included accompanying to the district hospital or psychologist, and counseling them about coping strategies to deal with side effects, such as discrimination; the AL Asha were trained to inquire about side effects, provide basic education and counseling, promote healthy life style choices, and link women living with HIV/AIDS with community resources to match health needs  **Duration (months):** 6 sessions lasting 45 minutes each  **Comparator**  The Usual Care participants received matched sessions in terms of number and length of time and generally included topics 1–3, as described for the Asha-Life program, followed by three additional sessions which were essentially question and answer type sessions, the women received standard education and some nutritional supplements  **Length of follow up:** 6 months | **Stigma results:** Paired t-tests and linear regression were used to measure program effects and found a reduction in internalized stigma (10-item scale by Ekstrand, p=0.001).  **Alternative results:** Found a reduction in avoidance coping (p=0.001).  **Unintended consequences:** NA |
| Peltzer, 2018 [40]  **Country:** South Africa  **Study design:** RCT NCT02085356  **Sample size:** N = 699  12 community centers  **Domain:** Internalized stigma | **Participant details:** Above the age of 18, HIV-seropositive, and between 8 and 24 weeks pregnant  **Cooccurring stigmas:** NA  **Context / setting:** Healthcare  **Study objective:** To increase prevention of mother to child transmission uptake in 12 rural community health centers in South Africa  **Implementer:** Government and academic research ers  **Definitions:**  NA | **Intervention**  The Enhanced Intervention condition included standard care prevention of mother to child transmission counseling as well as the 'Protect Your Family intervention' which consisted of 4 antenatal and 2 postnatal group sessions and were delivered by trained lay health workers; topics covered include HIV knowledge, adherence to prevention of mother to child transmission medicine, HIV disclosure, prevention of HIV transmission and stigma, communication with partners, intimate partner violence, and dual method sexual barrier use; the first two sessions focused on stigma in regards to communication with partners, medicine adherence, and HIV status disclosure  **Duration (months):** 6 total sessions conduced over 12 months  **Comparator**  Standard care counselling by nursing staff during perinatal care plus time-equivalent attention-control videos  **Length of follow up:** 14 months | **Stigma results:** HIV related stigma (Berger scale) and the four scale factors (personalized stigma, disclosure concerns, negative self-image, and concern public attitudes) decreased at follow-up in the intervention group while baseline scores of overall stigma and three scale factors (personalized stigma, negative self-image, and concern public attitudes) increased at follow-up in the control group.  **Alternative results:**  **Unintended consequences:** NA |
| Petersen, 2014 [41]  **Country:** South Africa  **Study design:** RCT  **Sample size:** N = 76  **Domain:** Internalized stigma | **Participant details:** Attending dedicated antiretroviral clinic for treatment, 18 years or older, not requiring urgent medical attention, no difficulty with hearing, speaking or cognition  **Cooccurring stigmas:** NA  **Context / setting:** Community,Other : Public clinic in KwaZuluNatal province  **Study objective:** To assess the feasibility of a group-based counselling intervention for depressed HIV-positive patients in primary health care in South Africa using a task shifting approach  **Implementer:** Academic researchers  **Definitions:**  NA | **Intervention**  Group-based Interpersonal Therapy intervention consisted of 8 session; each session comprised a number of steps starting with introducing a common trigger or exacerbating factor using a vignette; second step involved asking participants who identify with the story to share their problem; third step drew on problem management to address the triggers of depression and cognitive behavioral techniques for exacerbating factors (internalized stigma also addressed), promoting healthy thinking in the case of negative intrusive thoughts and behavioral activation for social isolation; fourth step involved getting participants to identify problems that they were going to work on in the next week; intervention was delivered by two of the lay HIV counsellors from the clinic who were trained in the intervention  **Duration (months):** 2 months  **Comparator**  Received normal standard of care which included the counseling services provided by the HIV counsellors  **Length of follow up:** 3 months | **Stigma results:** The facilitators identified the session on internalized stigma as most helpful as it helped to change the way the participants thought about themselves, which then helped them to withdraw less and have more hope.  **Alternative results:**  **Unintended consequences:** NA |
| Prinsloo, 2017 [42]  Prinsloo, 2016 [86]  **Country:** South Africa  **Study design:** Pre-post  **Sample size:** N = 632  62 people living with HIV, 570 community members, both live within the same municipal ward  **Domain:** Internalized stigma | **Participant details:** People living with HIV from 2 Department of Health clinics in NW South Africa; community members selected by random voluntary sampling from 780 households  **Cooccurring stigmas:** Received stigma  **Context / setting:** Healthcare,Community  **Study objective:** To explore, describe and determine whether an HIV stigma-reduction community “hub” intervention would change the HIV stigma experiences of people living with HIV  **Implementer:** Academic researcher and NGO (Optentia Research Focus Area)  **Definitions:**  Received stigma = how people act towards people living with HIV and includes all types of stigmatizing behavior towards people living with HIV, as experienced or described by themselves or others  Internalized stigma = thoughts and behaviors stemming from the affected persons’ own negative perceptions about themselves, based on HIV status  Associated stigma = results from an individual’s association with someone living with, working with or otherwise associated with people living with HIV, like health workers | **Intervention**  Two HIV stigma-reduction community hubs in the ward; each hub had a team comprising 2 mobilisers, 1 PLWH, and 1 non-infected person who had a close relationship with a PLWH; mobilisers lived in the ward and were involved in a previous HIV stigma-reduction study involving understanding and coping with HIV stigma, and the planning / implementation of their own HIV stigma-reduction community project, they underwent a four-day workshop to become community mobilisers and were trained to present workshops on “Understanding HIV stigma”, “Coping with stigma”, how to lead a support group, and also in effective record keeping of community activities during the intervention; 27 three-hour workshops on "Understanding HIV stigma” for both PLWH and community member groups; 5 workshops on “Coping with HIV Stigma” for those interested in continuing; and, weekly door-to-door “Understanding HIV stigma” teaching with a pamphlet; after the workshops a 6-session support group for both the community and PLWH groups were run; mobilisers presented 8 psychodrama group performances on the theme of “HIV stigma reduction” (eg, churches, gatherings and clinics); further mobiliser-activities included a HIV stigma-reduction community project with home visits, support and education at clinics, 2 stigma campaigns at taxi ranks and in main street  **Duration (months):** 5 months  **Comparator**  Baseline  **Length of follow up:** 5 months | **Stigma results:** The HIV/AIDS Stigma Instrument — PLWH (HASI-P, includes an internal stigma scale to assess negative self-perception) found a mean score of 36.92 before the intervention and 34.35 after the intervention, which showed no statistically (p = 0.09) but a small practically (d = 0.25) significant reduction in stigma experienced; the negative self-perception sub-scale found no statistically (p = 0.14) but a small practically (d = 0.21) significant difference post intervention.  **Alternative results:**  **Unintended consequences:** Participants re-lived traumatizing events when they described instances of verbal abuse, community gossip (“Hey did you hear? Your neighbor is sick”), being labelled on physical appearance or being mocked for physical inabilities, community came up with a name for people living with HIV (“hemela”, referring to dying and going to heaven) |
| Rao, 2012 [44]  **Country:** USA  **Study design:** Pre-post  **Sample size:** N = 24  **Domain:** Internalized stigma | **Participant details:** Recruited from a publicly funded HIV clinic through the use of advertisements in the form of flyers and promotion from the HIV clinic nurses  **Cooccurring stigmas:** Race  **Context / setting:** Healthcare  **Study objective:** To feasibility test and gather preliminary data on the effectiveness of the intervention to reduce symptoms of internalized stigma for African American women living with HIV  **Implementer:** Academic researchers  **Definitions:**  Public stigma = negative attitudes held by members of the public, such as health care professionals, clergy, or employers, about people with devalued characteristics that result in stereotypes, prejudice, and discrimination  Internalized stigma = Once public stigmas are enacted (personally experienced), they can be internalized by the stigmatized individual if the individual endorses the public stigmas | **Intervention**  Internalized stigma reduction intervention with break-out sessions which included discussions (to help participants develop new coping skills for stigma) and video presentations; discussions were encouraged after viewing the videos; afterwards, a structured discussion of key concepts followed; participants conducted also an exercises in which they tossed a yarned ball to each other and exclaimed "positive names for the web that had been created and [that had] linked them together  **Duration (months):** 2 consecutive afternoons with a 4-5 hour workshop  **Comparator**  Baseline  **Length of follow up:** 0.25 months | **Stigma results:** Stigma Scale for Chronic Illness showed decreased internalized stigma from the start of the workshop to the end (p = 0.05) and 1 week after ( p = 0.07).  **Alternative results:**  **Unintended consequences:** NA |
| Rao, 2018 [45]  Fabian, 2020 [87]  **Country:** USA  **Study design:** RCT NCT01893112  **Sample size:** N = 239  **Domain:** Internalized stigma | **Participant details:** Women who self-identified as having an African American racial/ethnic background, were 18 years of age or older, and had documentation of living with HIV  **Cooccurring stigmas:** Race  **Context / setting:** Community  **Study objective:** To assess the effect of a peer support workshop on HIV-related stigma among African American women living with HIV  **Implementer:** Academic researchers  **Definitions:**  NA | **Intervention**  The UNITY workshops (described as internalized stigma reduction intervention in prior studies) began with discussions of group expectations and what the term ‘stigma’ meant to them; after watching a “trigger” video, the facilitator lead a guided discussion; participants explored reactions to the video and personal experiences with stigma in a large group and in dyads; the next exercise involved brainstorming coping methods that group members have used to deal with stigma; in a segment about self-soothing, the facilitator lead a guided visualization; a subsequent segment included modeling and practicing assertiveness (vs passive or aggressive) in response to stigma; a self-esteem exercise and social support exercise finished out the first day; day two focused on disclosure with case studies and role play; the final exercise of UNITY engaged the group regarding how to “live positively” with HIV  **Duration (months):** 2 days  **Comparator**  A breast cancer education workshop was designed to match the UNITY workshop in terms of time and attention, and as such, was also held across two 4-hour sessions using similar group formats; the facilitator for this arm was one research coordinator per site; no peer was involved  **Length of follow up:** 12 months | **Stigma results:** Both arms experienced decreases in mean stigma scores (Stigma Scale for Chronic Illness measuring internalized and enacted stigma) over time; GEE analysis was used and reduction in stigma was not statistically significant between intervention and control (p=0.7308).  **Alternative results:**  **Unintended consequences:** NA |
| Rongkavilit, 2015 [46]  **Country:** Thailand  **Study design:** RCT  **Sample size:** N = 74  **Domain:** Internalized stigma | **Participant details:** Aged between 16–25 years, being HIV+ and understanding spoken and written Thai language  **Cooccurring stigmas:** MSM  **Context / setting:** Community  **Study objective:**  **Implementer:** Academic researchers  **Definitions:**  NA | **Intervention**  4-session motivational interviewing based Healthy Choices intervention  **Duration (months):** 6  **Comparator**  4 session control condition  **Length of follow up:** 6 months | **Stigma results:** There were no significant differences in HIV stigma scores between the two groups at any follow up point.  **Alternative results:**  **Unintended consequences:** NA |
| Shamsaei, 2020 [49]  **Country:** Iran  **Study design:** Pre-post  **Sample size:** N = 55  **Domain:** Internalized stigma | **Participant details:** Reside in Hamadan province, 6-month history of HIV positive, definitive diagnosis of the disease based on rapid test diagnostic tests, absence of any psychiatric diseases, lack of physical and mental disabilities, lack of a stressful experience in the last month; and minimum level of reading and writing literacy  **Cooccurring stigmas:** NA  **Context / setting:** Healthcare  **Study objective:** To determine the effect of stress management training on stigma and social phobia among HIV-positive women  **Implementer:** Healthcare nursing staff and psychologist of academic research center  **Definitions:**  Stigma = severely agonizing state that changes an ordinary person to an unimportant person with low self-esteem and self-efficacy | **Intervention**  Stress management training interventions in 5 group sessions offered weekly (45-60 minutes; introduction, stress definition, stress management skills, relaxation practice and diaphragmatic breathing, and barriers)  **Duration (months):** 1.25 months (1 session per week lasting 45 to 60 minutes each)  **Comparator**  Baseline  **Length of follow up:** 1.25 months | **Stigma results:** Berger HIV Stigma Scale (measuring personalized stigma, disclosure concerns, negative self-image, and public attitudes) found significant difference in stigma scores before and after the intervention (p<0.05).  **Alternative results:**  **Unintended consequences:** NA |
| Singh, 2020 [50]  **Country:** India  **Study design:** RCT NCT03746457  **Sample size:** N = 752  **Domain:** Internalized stigma | **Participant details:** Men who consumed at least one alcoholic beverage in the last 30 days, over 18 years of age, on antiretroviral therapy for at least 6 months  **Cooccurring stigmas:** Alcohol use  **Context / setting:** Other : Government antiretroviral therapy centers  **Study objective:** To identify the optimal sequence of the three interventions individual counseling, group intervention, and collective advocacy  **Implementer:** Government, academic researcher  **Definitions:**  Internalized stigma = self-blame for having contracted the disease  Anticipated stigma = People living with HIV expect a negative reaction from family, peers, and society and avoice situations where their expectations might be realized  Enacted stigma = involving discrimination experienced by people living with HIV | **Intervention**  The 3 interventions were individual counseling, group intervention, and collective advocacy; individual counseling and group intervention were based on formative research with study population, the existing government and non-government organizations guidelines, and infrastructure for individual counseling at antiretroviral therapy centers and group counseling at Community Support Centers and included an alcohol risk-reduction module and a significant component to address stigma (sources of internalized, external and relational stigma including disclosure); the third intervention was based on the successful HIV advocacy efforts of the Network of Maharashtra Positive People, a collaborating project partner, and prior research in India by the principal investigators on community level advocacy and structural change and literature on the role of activism in reducing HIV-related stigma globally  **Duration (months):** 27 months  **Comparator**  Received routine care approved by National AIDS Control Organization; the protocol called for people living with HIV at each visit to see the antiretroviral therapy counsellor who reviews antiretroviral therapy adherence, positive prevention and healthy living  **Length of follow up:** 27 months | **Stigma results:** Results show that there was a significant decrease in stigma measured by Berger’s HIV stigma scale during the third cycle of the intervention (p<0.001). Negative self-image as a  concern was successfully tackled by each intervention (group intervention, individual counselling and collective advocacy) alone, and each sequence of interventions.  **Alternative results:**  **Unintended consequences:** NA |
| Skinta, 2015 [52]  **Country:** USA  **Study design:** Pre-post  **Sample size:** N = 5  **Domain:** Internalized stigma | **Participant details:** HIV-positive gay- or bisexual-identified men who were receiving mental health services at an HIV community mental health clinic and a HIV diagnosis greater than 1 year prior  **Cooccurring stigmas:** Sexual orientation  **Context / setting:** Community  **Study objective:** To describe one example of the integration of acceptance and commitment therapy and compassion-focused therapy techniques  **Implementer:** Academic researchers  **Definitions:**  NA | **Intervention**  Incorporated ideas and exercises from compassion-focused therapy into acceptance and commitment therapy; the acceptance and commitment therapy consist of 6 core processes: contact with the present moment, delusion, acceptance, self-as-context, valuing, and committed action; session 1 focused on introducing and attuning the client to core acceptance and commitment therapy perspectives; session 2 covered costs and unworkability of responding to HIV-related stigma; session 3 began an exploration of values-driven behaviors to replace control efforts; session 4 continued with a discussion of acceptance and mindfulness and how to apply these concerns regarding HIV; session 5 explored the underlying principle of self-as-context versus self-as-content; session 6 explored the importance of willingness; session 7 introduced visualization of a compassionate friend; session 8 focused on generalizing acceptance, mindfulness, and delusion exercised learned during the course of the group  **Duration (months):** 8 sessions  **Comparator**  Baseline  **Length of follow up:** 2 months | **Stigma results:** 28-item scale developed with an urban, HIV-positive, predominantly gay male US sample assessed internalized HIV-related stigma and results showed two out of the three respondents reported lower HIV-related stigma.  **Alternative results:**  **Unintended consequences:** NA |
| Step, 2020 [56]  **Country:** USA  **Study design:** Pre-post  **Sample size:** N = 128  **Domain:** Internalized stigma | **Participant details:** Participants were between 13 and 34, HIV-positive, receiving care at MetroHealth, had access to a smartphone, diagnosed within the past 12 months or not fully engaged in HIV care (viral load > 200 copies/ml in past 24 months, did not have office visit or labs drawn in each f 6-month time periods during past 24 months)  **Cooccurring stigmas:** NA  **Context / setting:** Other : Social Media  **Study objective:** To explore stigma among Positive Peers users and gain understanding of how they may think about stigma in relation to the app  **Implementer:** Academic researchers  **Definitions:**  Stigmatization = sociocognitive process based on labeling human differences by others, associating stereotypes with these labels, categorizing and separating labeled others from larger society, and debased status and discrimination among the stigmatized, leading to unequal social outcomes  Internalized HIV stigma = enduring negative feelings and beliefs associated with HIV and applying them to oneself | **Intervention**  Positive Peers is a free mobile app that offers users gamified health management tools (e.g., medication and wellness trackers), SMS communication functions (i.e., forums and private chat), and over 250 HIV-related blogs and first-person narratives; the app is complemented with a website (www.positivepeers.org) and a daily dissemination of blog and other app news via various social media platforms (@PositivePeers4U)  **Duration (months):** NA  **Comparator**  Baseline  **Length of follow up:** 12 months | **Stigma results:** Overall stigma scores (10 question scale measuring negative feelings toward HIV, internalized stigma, concerns about status disclosure, and perceived public attitudes toward HIV) declined throughout the overall study period (n.s.).  **Alternative results:**  **Unintended consequences:** NA |
| Tsai, 2013 [57]  **Country:** Uganda  **Study design:** Pre-post  **Sample size:** N = 262  **Domain:** Internalized stigma | **Participant details:** Newly initiating antiretroviral therapy patients were 18 years of age or older and lived within 20 km of the clinic who are treatment naive  **Cooccurring stigmas:** NA  **Context / setting:** Unclear  **Study objective:** To determine whether participants’ time on HIV treatment was associated with reductions in stigma  **Implementer:** Academic research  **Definitions:**  Internalized stigma results when persons with a stigmatized attribute (such as HIV) accept prevailing discriminatory attitudes as valid | **Intervention**  Persons who tested positive for HIV infection and who met medical criteria for antiviral therapy were required to have 2 sessions of pre-treatment counseling before they were permitted to initiate ART; if a patient was thought to require immediate treatment, the counseling was either provided on the day of initiation or suspended altogether; supportive counseling was provided for patients experiencing psychosocial difficulties on an as-needed basis; ready-to-use therapeutic food supplements were given to patients who met criteria for extreme malnutrition; once patients were on a stable ART regimen, they generally returned to the clinic and had contact with a medical officer, clinical officer, or nurse 2–5 times annually  **Duration (months):** 40.8 months  **Comparator**  Baseline  **Length of follow up:**  41 months | **Stigma results:** Over time on treatment, internalized stigma (Internalized AIDS-Related Stigma Scale (IARSS)) declined steadily, with the largest decline observed during the first 2 years of treatment. This trend remained statistically significant after multivariable adjustment (P = 0.03), and appeared to be driven by ART-induced improvements in HIV symptom burden, physical and psychological wellbeing, and depression symptom severity.  **Alternative results:**  **Unintended consequences:** NA |
| Tsai, 2017 [58]  Cohen, 2015 [88]; Weiser, 2015 [89]  **Country:** Kenya  **Study design:** Controlled trial NCT01548599  **Sample size:** N = 54  **Domain:** Internalized stigma | **Participant details:** Aged 18-49 years, taking antiretroviral therapy at time of enrollment, had access to sufficient land and water for irrigation, either were classified as moderately to severely food insecure on the Household Food Insecurity Access Scale, or had a body mass index <18.5 documented in the medical records at any time during the year prior to enrollment, and expressed willingness to save the down payment (approximately $6 USD) required to qualify for a loan that was provided as part of the intervention  **Cooccurring stigmas:** NA  **Context / setting:** Other : In participant's home or anywhere of the participant's choosing.  **Study objective:** To explore the intervention’s perceived impacts on HIV stigma and to understand the mechanisms through which any stigma-related changes may have occurred  **Implementer:** Microfinance institution (private sector), NGO, and academic center  **Definitions:**  Anticipated/felt stigma = perception of negative attitudes and expectation of rejection from others, irrespective of whether enacted stigma actually occurs  Internalized stigma (or self-stigma) = acceptance of negative attitudes as valid, internalizing them, and the development of self-defacing beliefs | **Intervention**  The trial was conducted in the Rongo and Migori districts at government health facilities supported by Family AIDS Care and Education Services (FACES), a collaboration between the University of California at San Francisco and the Kenyan Medical Research Institute; the livelihood intervention consisted of a small loan (approximately $125 USD) to purchase a locally available handheld water pump and associated agricultural implements and commodities, along with an eight-session training program on agriculture and financial management. The training sessions were group-based. The intervention did not contain any of the psycho-educational components (e.g., information provision, counseling, or testimonials) that typically characterize anti-HIV stigma interventions  **Duration (months):** 8 sessions  **Comparator**  The control group did not receive any components of the livelihood intervention, after 12 months they were eligible to receive the intervention  **Length of follow up:** 12 months | **Stigma results:** Initially, participants felt ashamed of their seropositivity and were socially isolated (internalized stigma). At follow-up, participants in the treatment arm qualitatively described feeling less stigma and voiced positive changes in confidence and self-esteem; whereas previously participants reported ‘‘hiding’’ from the community in shame, subsequent to their participation in the intervention they felt less shame about their seropositivity and felt freer to disclose  **Alternative results:**  **Unintended consequences:** NA |
| Tshabalala, 2011 [59]  **Country:** South Africa  **Study design:** RCT  **Sample size:** N = 20  **Domain:** Internalized stigma | **Participant details:** HIV-positive African women who were receiving treatment from the clinic and who experienced difficulties in dealing with stigma, who have lived with HIV for at least 3 months have passed Grade 10, and came from a poor to average socio-economic background  **Cooccurring stigmas:** Enacted  **Context / setting:** Community  **Study objective:** To develop a therapeutic intervention to assist women to change their conception of HIV and their sense of self-worth  **Implementer:** Academic researcher  **Definitions:**  Internalized/felt stigma = stigmatized person's experience or fear of being stigmatized; affected persons often accept some of the negative social judgements that label and discredit them  Enacted stigma = actual experiences of stigmatization and resulting incidences of discrimination  Perceived community stigma = generalized perception of community attitudes | **Intervention**  A cognitive behavioral therapy model consisting of 8 individual sessions to address the 5 commonly identified themes that underlie the negative experiences of HIV-positive women; after each session, homework was assigned to facilitate change from one session to the next; the themes include feelings of powerlessness, feelings of guilt and anger about the past, destructive behavior patterns, experience of the reaction of others, and uncertainty about the future  **Duration (months):** 2 months, 8 sessions  **Comparator**  Control group were placed on a waiting list and received psychotherapy upon completion of the study  **Length of follow up:** 2 months | **Stigma results:** Pre- and post-test scores and found a statistically significant decrease in internalized stigma (Serithi Internalised Stigma Scale w; p<0.05).  **Alternative results:**  **Unintended consequences:** NA |
| Van Tam, 2012 [65]  **Country:** Vietnam  **Study design:** RCT  **Sample size:** N = 228  71 clusters  **Domain:** Internalized stigma | **Participant details:** HIV-positive patients who were ARV-naïve and eligible to initiate ART according to the Vietnamese national guidelines at the time of the study. Enrolled HIV positive patients who were ARV-naïve and eligible to initiate ART according to the Vietnamese national guidelines at the time of the study. Inclusion criteria were as following: clinical stage 4 of HIV disease (AIDS related illnesses) regardless of CD4+ count, clinical stage 3 (severe opportunistic infections) with CD4+ <350/μl, clinical stage 1 and 2 (asymptomatic or mild infection) with CD4+ count of <200//μl  **Cooccurring stigmas:** NA  **Context / setting:** Community  **Study objective:** To determine the effect of peer support on quality of life and internal stigma during the first year after initiating ART  **Implementer:** Academic researchers  **Definitions:**  NA | **Intervention**  Compared peer support impact on viral suppression and resistance development versus standard of care; intervention group participants received adherence support from trained peer supporters who visited participants’ houses biweekly during the first two months, and thereafter weekly  **Duration (months):** 12 months  **Comparator**  Compared to the standard guidelines that include adherence counseling, monthly health checks, and drug refills for the control group  **Length of follow up:** 12 months | **Stigma results:** The internal AIDS-related stigma scale measuring internal stigma (focused on self-blame and concealment of HIV status) did not differ between the intervention and control groups or between the different clinical stage groups after 12 months.  **Alternative results:**  **Unintended consequences:** NA |
| Wagner, 2012 [67]  **Country:** Uganda  **Study design:** Cohort study  **Sample size:** N = 602  **Domain:** Internalized stigma | **Participant details:** Consecutive new clinic clients who had just completed evaluation for ART eligibility who were 18 years or older, who were either about to start ART if eligible (had also to demonstrate treatment readiness by identifying a ‘‘treatment supporter’’ (typically a relative or a friend) and attend clinic regularly) or had CD4 cell count less than 400 cells/mm3 if not ART eligible  **Cooccurring stigmas:** NA  **Context / setting:** Healthcare  **Study objective:** To examine the effects of antiretroviral therapy on depression and other mental health indicators  **Implementer:** Civil Society (RAND and Clinical Research Center in Uganda)  **Definitions:**  NA | **Intervention**  Clients on antiretroviral therapy come to the clinic on a monthly basis at first and then bimonthly  **Duration (months):** 9 months  **Comparator**  Non-antiretroviral therapy patients had appointments between 2 and 6 months depending on CD4 count; counseling services were available to clients when requested or recommended by the provider, and generally consisted of pre- and post-HIV test counseling, antiretroviral therapy adherence counseling, and counseling related to HIV disclosure and sexual and reproductive health issues and all participants received HIV primary medical care, which includes monitoring and treatment of active infections, and prescription of appropriate prophylactic medications  **Length of follow up:** 12 months | **Stigma results:** Levels of internalized HIV stigma (8 item scale developed by Kalichman) decreased in the antiretroviral and the non-antiretroviral therapy group at month 6 but then decreased further for the antiretroviral group while the non-antiretroviral group returned to baseline levels at month 12 (p<0.001).  **Alternative results:**  **Unintended consequences:** NA |
| Watt, 2011 [68]  **Country:** Tanzania  **Study design:** Post only  **Sample size:** N = 28  28 patients and 11 treatment supporters  **Domain:** Internalized stigma | **Participant details:** Patients presenting at a government-supported faith-based clinic who had been taking ART for fewer than 6 months, and their treatment supporters (family member or friend who knew about their HIV status and provided, or had the potential to provide, support to help them live positively with HIV)  **Cooccurring stigmas:** NA  **Context / setting:** Community  **Study objective:** To assess the acceptability of a group intervention for HIV patients on antiretroviral therapy and their supporters  **Implementer:** Academic researchers  **Definitions:**  NA | **Intervention**  Participants (antiretroviral patients and their treatment supporters) attended a 4-hour seminar led by 2 HIV+ facilitators; activities included a presentation, roleplaying, and small group discussions; topics focused on basics about HIV and ART, caring for someone taking ART, stigma, and disclosure; roleplaying helped create a safe environment for participants to learn communication and behavioral skills and to facilitate open dialogue between patients and their supporters; antiretroviral patients and the treatment supporters were invited to attend separate focus group meetings the next week when the facilitators obtained feedback about the successes or failures of the intervention  **Duration (months):** 4 hours  **Comparator**  NA  **Length of follow up:** 0.25 months | **Stigma results:** Participants felt that the intervention reduced both their perceived and internalized stigma. Patients said they gained knowledge about adherence, felt empowered to tackle stigma and disclosure of their HIV positive status, and experienced reductions in feelings of loneliness. Both patients and supporters discussed how the intervention helped them to view HIV as a normal disease that affected many people, and that it therefore reduced their own perceptions of HIV stigma.  **Alternative results:**  **Unintended consequences:** NA |
| Watt, 2020 [69]  **Country:** Tanzania  **Study design:** RCT NCT03600142  **Sample size:** N = 1543  **Domain:** Internalized stigma | **Participant details:** Women who were pregnant, at least 18 years old, able to understand Swahili, were attending the first antenatal care appointment at one of the two government health centers, male partner accompanying the enrolled women were able to enroll  **Cooccurring stigmas:** Anticipated stigma  **Context / setting:** Healthcare  **Study objective:** The Maisha intervention aimed to address HIV stigma to improve early care engagement and to reduce HIV stigmatizing attitudes  **Implementer:** Academic researchers  **Definitions:**  Internalized stigma = negative beliefs and feelings about oneself  Enacted stigma = actual prejudice and discrimination from others  Anticipated stigma = fear of mistreatment that may occur if one's status is known | **Intervention**  Delivered by the facilitator to either the individual woman or the woman and her partner; sessions were conducted in a private research room; session 1 included a video and brief counseling session (depicting a couple who test positive for HIV during a first antenatal care visit, and follows them as they learn to accept their status, navigate disclosing their status to her mother-in-law, and commit to taking daily therapy); counseling provides psycho-education on various components of HIV stigma, lead participants to reflect on HIV stigmatizing attitudes, and encourage empathy and inclusion toward people living with HIV in the community; session 2 was delivered immediately following the first antenatal appointment to all women identified living with HIV, along with their male partners, if enrolled, returning to content to discuss how issues of stigma might relate to the couple's situation and to reduce barriers of HIV care; session 3 asked women living with HIV to come alone to discuss relationship concerns, Third Wave cognitive behavioral concepts are introduced in this session, the facilitator emphasizes that one’s feelings (internalized stigma) and thoughts (anticipated stigma) are connected. Finally, the counselor and participants consolidate the components of Maisha into an individualized action plan, which includes goals for HIV care engagement and antiretroviral therapy adherence, strengthening social support and considering selective HIV disclosures  **Duration (months):** 0.75 months  **Comparator**  Received the standard of care HIV testing and counseling protocol in the clinic administered by clinic nurses, education about HIV and HIV testing; if an individual tests positive, counseling is initiated and women living with HIV should be registered for prevention of mother-to-child transmission care and immediately initiated on antiretroviral therapy, which is provided during the clinic appointment.  **Length of follow up:** 3 months | **Stigma results:** Participants in the intervention condition had greater reductions in internalized stigma measured by Scale A of the HIV and Abuse Related Shame Inventory (HARSI) (β=− 3.5; 95% CI − 9.4, 2.4) and lesser reductions in anticipated stigma than the control group (β=2.0; 95% CI − 3.3, 7.3).  **Alternative results:**  **Unintended consequences:** NA |
| Yigit, 2020 [71]  Modi, 2018 [90]  **Country:** USA  **Study design:** RCT NCT01900236  **Sample size:** N = 372  **Domain:** Internalized stigma | **Participant details:** 18 years or older, HIV-positive, initiating HIV care at one of the four participating academically affiliated HIV clinics, spoke English, not planning on moving in 12 months  **Cooccurring stigmas:** NA  **Context / setting:** Healthcare  **Study objective:** To address HIV treatment adherence may have the added benefit of reducing internalized stigma  **Implementer:** Academic researchers  **Definitions:**  HIV-related stigma = complex social process in which people living with HIV experience prejudice, devaluation, discrimination, rejection, and other negative attitudes because of the fact that they are living with HIV  Internalized HIV-related stigma = accepting and adopting negative evaluations held in society about people living with HIV and applying these evaluations to oneself | **Intervention**  The first face-to-face intervention session focused on introducing the participant to the iENGAGE intervention, providing basic education on HIV and HIV care, including importance of attending all medical appointments, and an opportunity to process HIV diagnosis and develop trust and rapport with the iENGAGE counsellor. The follow-up sessions focused on maintaining a positive approach and participating in skills building modules with the counsellors. Another goal was to identify and combat barriers to treatment adherence individually for each participant, including HIV-related stigma. Each session included a review of goals from the prior session and adjustment to living with HIV. Counsellors addressed questions raised by participants. At the final session, methods for maintaining intervention gains in the future were discussed  **Duration (months):** 4 sessions  **Comparator**  Received standard of care at each participating clinic site; included various strategies to support antiretroviral therapy adherence and retention in care  **Length of follow up:** 12 months | **Stigma results:** There was a statistically significant reduction in internalized stigma (HIV Stigma Scale) for those in the intervention arm at follow-up compared to baseline (p=0.000).  **Alternative results:**  **Unintended consequences:** NA |

Notes: CI confidence interval, duration: intervention duration; length of follow up: outcome assessment from baseline; NA not available, not applicable

The evidence table shows all studies meeting eligibility criteria (either the intervention focused on internalized stigma, or the study assessed the effect on internalized stigma). Main analyses across all studies are shown in the main manuscript. Restricting to the smaller set of studies that evaluated interventions with a specific focus on internalized stigma reduction as a sensitivity analysis showed a reduction across studies, but it was not statistically significant (SMD 0.30; CI 0.03, 3.26; 5 studies) and heterogeneity remained high (I^2^ 99%).

## Geographical Distribution of Identified Interventions to Address Internalized Stigma


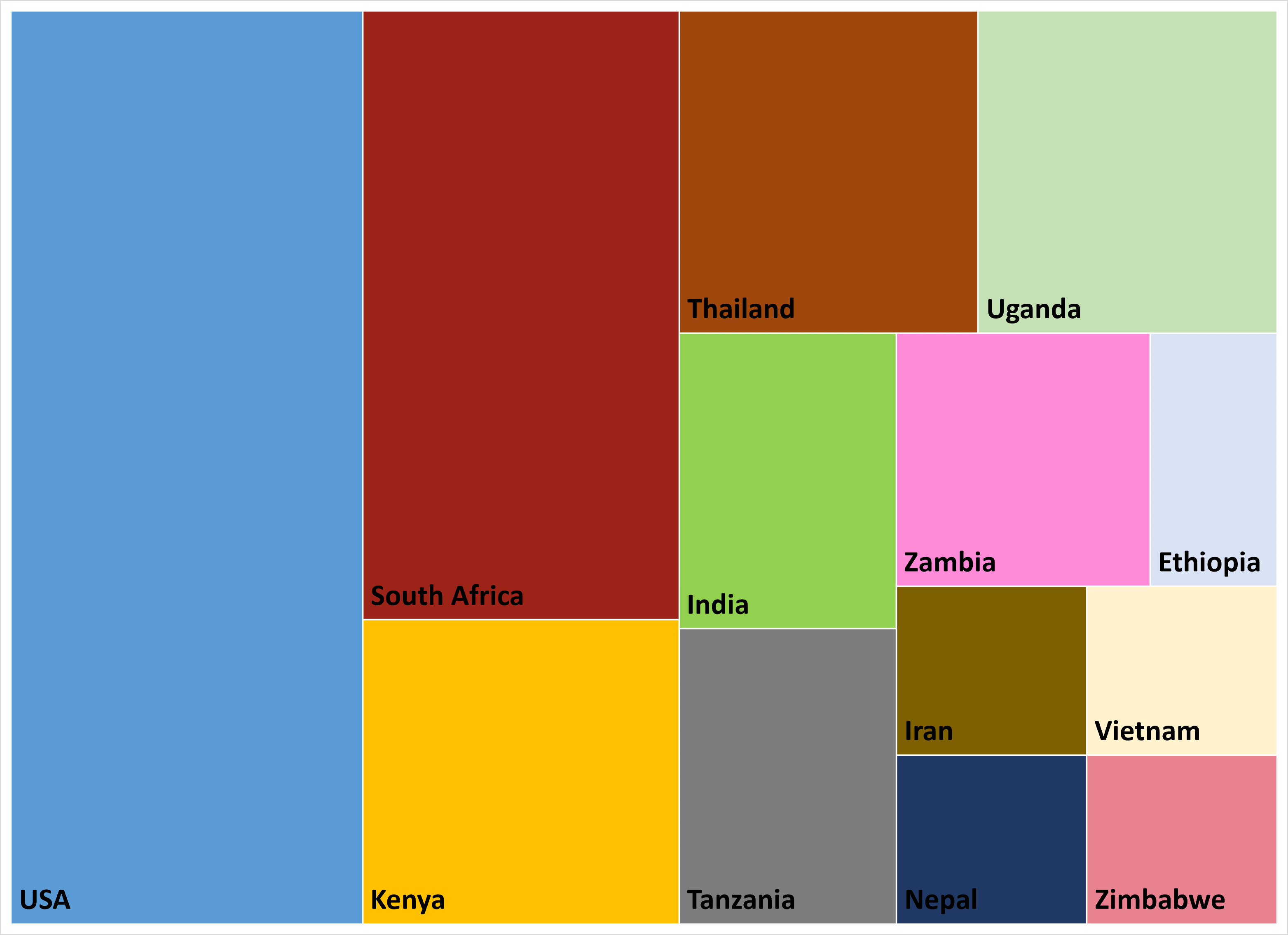


## Evidence table: Interventions for Healthcare Settings

| **Author, year**  **Country**  **Study design**  **Domain** | **Participant details**  **Sample size**  **Study objective**  **Cooccurring stigmas**  **Definitions** | **Intervention**  **Duration**  **Comparator**  **Duration**  **Length of follow up** | **Stigma results**  **Alternative results**  **Unintended consequences** |
| --- | --- | --- | --- |
| Arora, 2014 [4]  **Country:** India  **Study design:** RCT  **Domain:** Healthcare | **Participant details:** Female student nurses studying in third year of BSc nursing and General nursing  **Sample size:** N=65  **Study objective:** To assess the effectiveness of an empowering programme on student nurses' understanding and beliefs related to HIV/AIDS  **Implementer:** Academic researchers  **Cooccurring stigmas:** NA  **Definitions:**  NA | **Intervention**  A five day empowering program was prepared in consultation with eight experts from community medicine and nursing field to expand the understanding of student nurses and modify their beliefs related to HIV/AIDS. Content focused on the magnitude, basic dynamics, mode of transmission and prevention of HIV/AIDS, and altering the beliefs of student nurses about HIV infection and AIDS; lecture, group discussion, role play and case based scenarios were used in the role plays to bring about a change in the thought process of students  **Duration (months)**: 0.2  **Comparator**  No intervention  **Length of follow up:** 0.1 months | **Stigma results:** There was a significant difference observed in the mean posttest understanding and belief scores (questionnaire assessing magnitude of HIV/AIDS, mode of transmission,  management and prevention) between the experimental and control group (p<0.05).  **Alternative results:**  **Unintended consequences:** |
| Kaponda, 2009 [20]  **Country:** Malawi  **Study design:** Pre-post  **Domain:** Healthcare | **Participant details:** Malawian urban clinical and non-clinical hospital workers  **Sample size:** N=855  **Study objective:** To affect health workers’ personal HIV prevention behaviors as well as HIV prevention at the workplace in clinic  **Implementer:** Academic researchers  **Cooccurring stigmas:** NA  **Definitions:**  Stigma = negative attitudes held by health workers include stigmatization of persons living with HIV; reluctance to discuss sexuality issues, especially with young people; and a sense of hopelessness about combating the epidemic | **Intervention**  Sessions were implemented by trained peer-group facilitators; the Mzake ndi Mzake intervention for community hospital workers consisted of 6 sessions focused on the HIV epidemic and stigmatization; human sexuality and sexually transmitted infections; HIV prevention, AIDS, and testing; partner negotiation; condom use; and how to contribute to community HIV prevention; 4 new sessions were added for health workers only on HIV issues they reported that they frequently encountered: a nontechnical overview of HIV treatment and symptom management, universal precautions, helping individuals and families address HIV prevention, and ethical issues for health workers related to HIV. Every session included guided discussions, role plays, return demonstrations with corrective feedback, and an assignment to practice a specific skill before the next session  **Duration (months)**: 90-120 minutes; 0.03 months  **Comparator**  Pre intervention data on urban hospital workers, using unmatched samples of health workers  **Length of follow up:** 10 months | **Stigma results:** Compared with the baseline survey, after the intervention, workers had higher knowledge of HIV transmission and prevention; more positive attitudes including more hope, less stigmatization of persons with HIV.  **Alternative results:** Despite increased self-efficacy for safer sex and increased discussions about safer sex with partners, hospital workers showed no change in their reported risky sexual behaviors from baseline to the final evaluation. The proportion of hospital workers who reported that they had an HIV test in the previous 12 months increased significantly from 20.8% at baseline to 40.8% at the final evaluation. Health workers reported whether during the previous 2 months they had engaged in each of six HIV prevention activities when outside the hospital environment. The mean number of HIV prevention activities increased significantly from 3.98 to 4.92, an increase of nearly one more activity.  **Unintended consequences:** NA |
| Li, 2013 [22]  Li, 2015 [91]; Li, 2013 [92]; Li, 2014 [93]; Li, 2013 [94]  **Country:** China  **Study design:** RCT  **Domain:** Healthcare | **Participant details:** Health providers (aged 18 or older) who had regular contact with patients, including doctors, nurses, and lab technicians  **Sample size:** N=1760  40 hospitals; 2 provinces  **Study objective:** To reduce service providers’ stigmatizing attitudes and behaviors toward people living with HIV  **Implementer:** Academic researchers  **Cooccurring stigmas:** NA  **Definitions:**  HIV-related stigma and discrimination = refusal of care, suboptimal services, excessive precautions, isolation, mandatory testing, breach of confidentiality, humiliation, and blaming | **Intervention**  Popular opinion leaders attended 4 group sessions over a 1-month period and 3 reunion sessions; sessions covered complying with universal precaution procedures and ensuring occupational safety, fighting against stigma and improving the provider---patient relationship, taking actions and making efforts to care for patients, and overcoming difficulties and building up a better medical environment; trained popular opinion leaders providers were inspired to serve as behavior change endorsers and disseminate intervention messages to their coworkers; interactive techniques were used to effectively deliver the messages to other providers in the hospital  **Duration (months)**: 1 month, sessions usually lasted about 1.5 hours  **Comparator**  Popular opinion leaders among the health service providers were not identified or trained for the hospitals in the control group.  Both intervention and control hospitals received standard information packages on general safety in medical procedures and the same amount of universal precaution supplies (e.g., disposable sharps containers, medical disposable clothes, waterproof aprons, protective goggles, and rubber gloves) from the Chinese Center for Disease Control and Prevention  **Length of follow up:** 12 months | **Stigma results:** Mean reductions in prejudicial attitudes (formed on the basis of the 12-item priority stigma indicator) reported by providers in the intervention were 2.72 (13% reduction from baseline) and 4.63 (22.1% reduction from baseline) at the 6- and 12-month follow-ups, whereas those in the control group were less than 5% reduction from baseline; showing significant improvements in pre-post intervention time trends for the intervention group in reducing prejudicial attitudes (P< .001).  **Alternative results:**  **Unintended consequences:** NA |
| Lohiniva, 2016 [24]  **Country:** Egypt  **Study design:** Controlled trial  **Domain:** Healthcare | **Participant details:** All physicians and nurses in the surgical units in the selected hospitals were invited to voluntarily join the study  **Sample size:** N=471  **Study objective:** To evaluate the impact of an intervention to reduce stigma associated with HIV among doctors and nurses working in surgical unit  **Implementer:** Government and NGO  **Cooccurring stigmas:** NA  **Definitions:**  Stigma in healthcare = denial of care and medicines, passing HIV-positive patients from one health provider to another, isolating HIV-positive patients from other patients, and use of unnecessary infection control measures | **Intervention**  Five interactive training modules including discussions and practical exercises: a module on HIV background and stigma, on medical ethics, on childbirth, and two modules addressing infection prevention and control measures, including standard precautions and aseptic techniques for invasive procedures  **Duration (months)**: 4 months  **Comparator**  No intervention in control hospital  **Length of follow up:** 3 months | **Stigma results:** Value-based and fear-based stigma (measures developed in the study) showed significant decreases from baseline compared to the control group (p<0.001).  **Alternative results:**  **Unintended consequences:** NA |
| Mak, 2015 [27]  **Country:** China  **Study design:** RCT  **Domain:** Healthcare | **Participant details:** Healthcare professional students  **Sample size:** N=88  **Study objective:** To test HIV stigma-reduction intervention that focused on increasing sensitivity to the feelings of people living wit HIV  **Implementer:** Government and NGO  **Cooccurring stigmas:** Sexual orientation  **Definitions:**  NA | **Intervention**  The intervention focused on increasing sensitivity to the feelings of people living with HIV through experiential games followed by group discussion; inspired by Cornett’s empathic learning intervention, activities were designed with three goals in mind: to allow participants to gain first-hand experience of potentially stressful situations that people with HIV might encounter on a day-to-day basis; to encourage participants to experience the feelings and thoughts of people living with HIV and to allow them to acknowledge their own sense of vulnerability and helpfulness in the face of similar stressors; and to provide a platform for participants to talk openly; immediately following a 30-minute didactic session on HIV/AIDS knowledge, the group participated in two different experiential games.  **Duration (months)**: 90-minute sharing session; 0.03 months  **Comparator**  The in vivo contact group listened to a 90-minute sharing session hosted by two PLHIV  **Length of follow up:** 1 months | **Stigma results:** The effectiveness of the game-based experiential approach in reducing HIV-related stigmatizing attitudes (14 items from three previous studies) was similar to that of in vivo contact (difference p >0.05).  **Alternative results:**  **Unintended consequences:** NA |
| Mbela, 2011 [30]  **Country:** Malawi  **Study design:** RCT  **Domain:** Healthcare | **Participant details:** Intervention and control clinical and nonclinical district health workers at five rural health centers  **Sample size:** N=417  **Study objective:** To examine the impact of a peer group intervention on personal and community HIV prevention-related knowledge, attitudes, and behaviors for rural health workers  **Implementer:** Academic researchers  **Cooccurring stigmas:** NA  **Definitions:**  NA | **Intervention**  The intervention addressed primary prevention of HIV infection for health workers in their personal lives and workplace; 10 sessions provided information and skills; the first 6 sessions focused on HIV transmission, stigmatization, safer sex, and partner negotiation and were used for both health workers and community members; the remaining sessions addressed universal precautions and teaching clients about HIV; sessions included guided discussions, role playing, return demonstrations with corrective feedback, and skill-building assignments; 2 co-facilitators offered the intervention to mixed gender groups of 10-12 health workers; groups were divided into clinical and nonclinical workers except at very small clinics; project staff offered the initial peer groups but some health workers volunteered to be co-facilitators; volunteers received training in the peer group content, learning activities, and group facilitation skills with practice and corrective feedback  **Duration (months)**: 10 sessions each lasting 90-120 minutes over undeclared time period  **Comparator**  no intervention  **Length of follow up:** 30 months | **Stigma results:** At 30 months, intervention district workers also reported less stigmatizing attitudes (measured by blaming a PLWH for being infected and acceptance of casual contact toward persons living with AIDS).  **Alternative results:**  **Unintended consequences:** NA |
| Mockiene, 2011 [32]  **Country:** Lithuania  **Study design:** RCT  **Domain:** Healthcare | **Participant details:** Lithuanian registered nurses working in three randomly selected hospitals among the nine largest hospitals in Lithuania, and in primary health care centers attached to those  **Sample size:** N=206  Assignment by hospital, 3 hospitals  **Study objective:** To test whether an education intervention could increase nurses’ knowledge and positively change their attitudes toward HIV-positive patients  **Implementer:** Academic researchers and NGO (Lithuanian AIDS Centre)  **Cooccurring stigmas:** Homophobia  **Definitions:**  NA | **Intervention**  Group 1: 2-day workshop and distribution of written material  Group 2: distribution of only written materials  **Duration (months)**: 2 days  **Comparator**  No intervention  **Length of follow up:** 0.1 months | **Stigma results:** The 2-day workshop and written materials had a positive effect on knowledge levels (p=0.001) but written materials alone failed to improve nurses’ knowledge or change their attitudes.  **Alternative results:**  **Unintended consequences:** |
| Norr, 2012 [34]  Ferrer, 2011 [95]  **Country:** Chile  **Study design:** RCT  **Domain:** Healthcare | **Participant details:** Participants were selected from two municipalities, Interested community clinic health workers from select intervention or control clinics in Chile; 5 non-STD health clinics in each municipality (total clinics = 10) supplied the participants (community clinic workers)  **Sample size:** N=555  **Study objective:** To describe a professionally facilitated peer group intervention for HIV prevention community health workers in Chile  **Implementer:** Academic researchers  **Cooccurring stigmas:** NA  **Definitions:**  NA | **Intervention**  The intervention (Mano a Mano Para Trabajadores de Salud) is a professionally-assisted peer group intervention with groups of 10-12 health workers attending 8 sessions (90 minutes each) covering (a) the importance of community HIV prevention; (b) standard precautions in the health care setting; (c) HIV testing treatment in Chile; (d) offering care that respects human dignity and confidentiality; (e) human sexuality, sexual transmission of HIV, and HIV transmission through drug use and blood; (f) partner communication and HIV prevention; (g) counseling about HIV infection; and (h) teaching HIV prevention to clients and families; each session incorporated active learning activities such as role-plays; separate groups were created for the professional/technical staff and the less educated paraprofessional / ancillary staff  **Duration (months)**: 8 sessions lasting 90 minutes  **Comparator**  Waitlist, same intervention offered to control group three months after intervention group  **Length of follow up:** 3 months | **Stigma results:** The intervention group expressed less stigmatizing attitudes toward general contact (p < 0.01) and patient contact (p < 0.001) compared to the control group after the intervention.  **Alternative results:**  **Unintended consequences:** NA |
| Nyblade, 2018 [36]  **Country:** Tanzania  **Study design:** Pre-post  **Domain:** Healthcare | **Participant details:** Facility staff and clients living with HIV (adults and youth)  **Sample size:** N=564  **Study objective:** To implement a total facility approach to reduce HIV-related stigma and discrimination in health facilities that focuses on stigma  **Implementer:** Academic researchers, NGO (Kimara Peer Educators and Promoters Trust), and Government  **Cooccurring stigmas:** NA  **Definitions:**  NA | **Intervention**  Health Policy Plus project total facility approach to stigma and discrimination reduction all stages of the treatment cascade in health facilities; the intervention promoted a sustainable response through capacity building, facility ownership, and youth engagement; champion teams were identified in each facility and empowered by facility management to work collaboratively to reduce stigma and discrimination; activities carried out by the teams included declaring and drawing attention to facilities’ commitment to stigma-free care via community TV and radio spots and promoting client engagement and accountability through the creation and display of codes of conduct, signboards, nametags, t-shirts, and reporting boxes  **Duration (months)**: 3 months  **Comparator**  Baseline  **Length of follow up:** 7 months | **Stigma results:** Significant reductions in stigmatizing attitudes among facility staff, as measured by levels of agreement with a series of attitudinal questions capturing judgment and shame toward people living with HIV, women living with HIV, and youth. The largest change was in agreement with the statement that people living with HIV do not care if they infect others (decline from 80% to 34%; p=.000). Composite indicators showed a decline in stigmatizing attitudes about women living with HIV (from 91% to 58%; p=.000) and a decline in stigmatizing attitudes toward sexually active adolescents (from 97% to 74%; p=.000). The health facility environment also improved; the hesitancy of staff to be tested for HIV in their facilities due to fear of others’ reactions declined (from 41% to 7%; p=.000) and perceptions that colleagues living with HIV would be hesitant to seek treatment in their facility also declined (from 45 to 15%, p=.000).  **Alternative results:**  **Unintended consequences:** NA |
| Nyblade, 2020 [37]  Nyblade, 2018 [96]; PEDFAR, 2020 [97]  **Country:** Ghana  **Study design:** Controlled trial  **Domain:** Healthcare | **Participant details:** Facility management and clinical and non-clinical health staff  **Sample size:** N=2308  10 health care facilities  **Study objective:** To evaluate the impact of a total facility stigma-reduction intervention on the drivers and manifestations of stigma and discrimination among health facility staff in Ghana  **Implementer:** Champion teams lead by NGO  **Cooccurring stigmas:** Sexual orientation  **Definitions:**  Stigma = powerful social process characterized by labelling (distinguishing differences), stereotyping (attributing negative characteristics to the distinguished differences), separation (through physical and social isolation), leading to status loss (soc | **Intervention**  Participatory data dissemination and review workshops with staff; in the workshops, staff identified stigma and discrimination challenges and generated potential solutions; targeting the whole facility beyond HIV services, the intervention approach included a 2-day participatory stigma-reduction training for all staff levels (clinical and non-clinical) with delivery by staff and clients from the facilities trained as stigma-reduction facilitators; trainings were delivered to all categories of staff, with a target of reaching 70% of the facility workforce; trainings were based on pre-existing global training materials targeted towards actionable HIV stigma drivers and were adapted to the Ghanaian context through an in-country stake- holder workshop; training coverage in individual facilities ranging from 61% to 97%; each facility also created an 8-10 member “champion team,” which was provided $5000 USD to develop facility-specific ancillary activities, including launch events, anti-stigma-and-discrimination banners and posters, additional staff trainings, printed codes of ethics, reporting mechanisms, and staff name tags to enable identification and reporting of stigma and discrimination  **Duration (months)**: 3 months  **Comparator**  Non-intervention facilities health care staff received only HIV information and identification of stigma and discrimination challenges and identified potential solutions  **Length of follow up:** 6 months | **Stigma results:** Favorable intervention effects for all outcome domains except for stigmatizing attitudes (3 variables) in both the full sample and the medical staff-only subsample. Preferring not to provide services to people living with HIV or a key population member improved by 11.1% points (CI 3.2, 19.0) more in the intervention than comparison facility respondents. Reported observation of stigma and discrimination incidents fell by 7.4% points more among intervention than comparison facility respondents (p = 0.06). Respondents at intervention facilities were 19.0% (CI 12.2, 25.8) more likely to report that staff behavior towards PLHIV had improved over the last year than those at comparison facilities.  **Alternative results:**  **Unintended consequences:** NA |
| Odeny, 2013 [38]  **Country:** Kenya  **Study design:** Pre-post  **Domain:** Healthcare | **Participant details:** 40 health facilities staffed by 200 healthcare workers (144 were nurses); recruited patients were ≥18-year old, HIV-positive and already enrolled in HIV care  **Sample size:** N=343  **Study objective:** To assess the patient-level effect of the integration of HIV services into primary care with a focus on patient satisfaction and perceived stigma  **Implementer:** Civil Society (FACES advocacy), UCSF academic research center, and Kenya government  **Cooccurring stigmas:** Perceived  **Definitions:**  NA | **Intervention**  Co-location and sharing of services and resources for HIV care and primary care including clinic space, clinicians, lab work, and health education for patients; intensive training on commodity management and streamlined supply chain, staff training for HIV  **Duration (months)**: NA  **Comparator**  Baseline  **Length of follow up:** 12 months | **Stigma results:** Patient perception of stigma at the health facility was assessed using three items adapted from standardized items validated for use in this context. Items covered privacy and confidentiality, equitable treatment for HIV-positive people, and discomfort receiving care at the health facility due to the possibility of other people finding out one’s HIV status. At 12 months after integration, respondents were more likely to be satisfied with reception services (adjusted odds ratio, aOR 2.71, CI 1.32–5.56), HIV education  (aOR 3.28, CI 1.92–6.83), and wait time (aOR 1.97 CI 1.03–3.76). Men’s comfort with receiving care at an integrated clinic did not change (aOR = 0.46 95% CI 0.06–3.86). Women were more likely to express discomfort after integration (aOR 3.37 95% CI 1.33–8.52). Integration did not heighten perceived stigma. Before and after integration, patients generally agreed that care was provided confidentially and equitably regardless of HIV status although women expressed increased discomfort with receiving care at integrated clinics.  **Alternative results:**  **Unintended consequences:** NA |
| Pulerwitz, 2015 [43]  Khuat, 2008 [98]  **Country:** Vietnam  **Study design:** Controlled trial  **Domain:** Healthcare | **Participant details:** Health care staff (clinical, administrative, and support)  **Sample size:** N=795  **Study objective:** To discover if combined a fear-based plus social stigma reduction intervention was more effective than an intervention that primarily focused on fear alone reduction  **Implementer:** NGO  **Cooccurring stigmas:** Fear-based stigma, value-based stigma, enacted stigma, compounded stigma  **Definitions:**  Stigma = attribute that is deeply discrediting within a particular social interaction  HIV stigma = pattern of prejudice, discounting, discrediting and discrimination directed at people perceived to have HIV, their significant others and close associates, | **Intervention**  The intervention included 6 key components: establishment of a hospital steering committee, staff training, hospital policy development, provision of material supplies to facilitate the practice of universal precautions, provision of educational materials, and monthly monitoring; staff from the other northern and southern hospitals received a half-day training on basic HIV/AIDS information that included testimonials from HIV-positive individuals, and a full day on Universal Precautions; in addition, an extra half-day training on social stigma; training sessions on HIV and stigma were co-facilitated by people living with HIV  **Duration (months)**: Day and a half of training; 0.07 months  **Comparator**  Half-day training on basic HIV/AIDS information that included testimonials from HIV-positive individuals, and a full day on Universal Precautions only  **Length of follow up:** 7 months | **Stigma results:** Following the intervention, the mean score on both the fear- and value-based stigma indices had significantly decreased in all 4 hospitals (p < .05). The stigma intervention showed more impact: hospital workers reported significantly less fear about changing the clothes of a patient with HIV (p < .001), hospital workers who felt that HIV/AIDS is a punishment for bad behavior declined (p < .001). Four items that measured fear-based stigma and five items that measured value-based stigma were each summed to create a composite score (Fear: Cronbach’s alpha = .74; Value: Cronbach’s alpha = .81).  **Alternative results:**  **Unintended consequences:** NA |
| Siraprapasiri, 2020 [51]  **Country:** Thailand  **Study design:** Pre-post  **Domain:** Healthcare | **Participant details:** Health-facility staff and people living with HIV clients  **Sample size:** N=7482  7482 staff in 44 hospitals  **Study objective:** To share Thailand’s journey through development, implementation, and lessons learned for one of the first national stigma and discrimination-reduction responses globally  **Implementer:** Government officials and agencies; civil society advocates  **Cooccurring stigmas:** Sexual orientation, gender identity, sex work  **Definitions:**  Stigma = social process that consists of identification and labeling of difference, attribution of negative attributes to that difference, separation (us versus them), leading to status loss and discrimination  Discrimination = unfair and unjust action tow | **Intervention**  Three building blocks were implemented: policy and its translation into a roadmap for action; measurement development and routinization to inform intervention design and track progress; and intervention development and implementation of a health facility stigma-reduction package which included 10 training modules spanning 12 hours and covering the actionable drivers of stigma and discrimination, human rights, a client panel, and action planning  **Duration (months)**: 6 hours of training delivered in either for 1 full day or across 2 afternoons  **Comparator**  Baseline  **Length of follow up:** 12 months | **Stigma results:** The Health Facility Staff questionnaire demonstrated significant reduction in key stigma indicators as measured with HFS and PLHIV-clients; use of unnecessary infection control precautions with people living with HIV dropped from 61 to 43.2% (P< 0.01).  People living with HIV reported experienced discrimination in health facilities in the past 12 months dropped from 10.9 to 7.9% (P< 0.01).  **Alternative results:**  **Unintended consequences:** NA |
| Sommerland, 2020 [53]  **Country:** South Africa  **Study design:** RCT  **Domain:** Healthcare | **Participant details:** Professional healthcare workers (doctors, nurses, and allied health professionals such as social workers and physiotherapists); administrative and management staff; and support staff (e.g. porters, cleaners, security staff, and household staff such as kitchen and laundry workers)  **Sample size:** N=652  8 hospitals  **Study objective:** To develop and test an HIV- and TB stigma-reduction intervention in public hospitals in the Free State Province of South Africa  **Implementer:** Change agents from academic research center  **Cooccurring stigmas:** Tuberculosis  **Definitions:**  Stigma = process of differentiation and othering that can cause discrimination and modified behavior for those targeted; occurs when a certain characteristic is negatively regarded and the person labeled is devalued | **Intervention**  The intervention to reduce HIV and tuberculosis stigma among health care workers consisted of a workshop for health care workers who would educate other health care workers on how to reduce stigma among colleagues, and a social marketing campaign to help reinforce and disseminate the single key anti-stigma message in the workplace: ‘Let’s Stop Stigma – Be kind to yourself and others; it was also translated into Afrikaans and Sesotho; in the social and behavior change communication intervention, participants were taught about different forms and effects of stigma and the related health rights and responsibilities in the workplace, and were equipped to start conversations about HIV and TB stigma in the workplace with co- workers underpinned through an exercise in confronting one’s own beliefs, values, attitudes, and behaviors; they were also taught how to respond to stigmatizing situations in the workplace, received social marketing materials (e.g. branded wristbands and pens) to support their stigma-reduction communications; staff identified change agents; there was a visible social marketing campaign, including posters that the health care workers could point to, to spark conversations or highlight what they are saying; medical doctors who were not able to attend the general staff training were educated stigma through presentations based on workshop materials  **Duration (months)**: 12 months  **Comparator**  Unit of randomization was the hospitals in which the participants worked; from each hospital, a selection of healthcare workers participated  **Length of follow up:** 24 months | **Stigma results:** There was no significant intervention effect on the respondents’ external HIV stigma (stigmatising others either through perceptions, attitudes or behaviors; p = 0.34), and the rank-biserial correlation of the ranks was r = 0.50 in the direction that stigma was lower in the control group compared to the intervention. Neither did co-workers’ external HIV stigma show an intervention effect (p = 0.49), even if the effect size (r = 0.38) was in the direction that hospital-level stigma scores tended to be higher in the intervention group.  **Alternative results:** Qualitative evidence reported new awareness and changed behavior related to HIV and TB stigma among individual healthcare workers, but a combination of factors including strong social hierarchies in the workplace and the down-scaling of the original version of the intervention seemed to reduce the impact  **Unintended consequences:** NA |
| Srinivasan, 2021 [55]  Ekstrand, 2020 [99]; Radhakrishna, 2017 [100]; Nyblade, 2018 [101]  **Country:** India  **Study design:** RCT  **Domain:** Healthcare | **Participant details:** Nursing students from 28 nursing schools that included private, nonprofit, and government-run nursing schools in the state of Karnataka, India who were aged 18 years or older and  willing to participate  **Sample size:** N=3182  28 hospitals  **Study objective:** To examine the mediating effects of changes in stigma drivers following an intervention for nursing students from south India  **Implementer:** Academic researchers and NGO  **Cooccurring stigmas:** NA  **Definitions:**  Instrumental Stigma = fears and misconceptions regarding HIV transmission during casual contact  Symbolic Stigma = pre-existing negative attitudes toward marginalized groups vulnerable to HIV | **Intervention**  The intervention comprised of 2 self-guided sessions administered on a computer tablet and a skills-based group session co-led by study staff and a person living with HIV from the local network; there were 4 modules in each session and a study member was available to guide the participants through the sessions and answer questions; the intervention used videos and interactive exercises to increase awareness of stigma in health care settings, improve HIV transmission knowledge, develop empathy for people living with HIV, address casual transmission fears, and teach the correct use of standard precautions with all patients; the group session with people living with HIV included a discussion of experiences with health providers, review of key lessons learned, role playing exercises using common stigma situations encountered in hospital settings, and the session concluded with participants making stigma reduction commitments  **Duration (months)**: 3 sessions total; 2 self-guided sessions and 1 group session  **Comparator**  Wait list  **Length of follow up:** 12 months | **Stigma results:** Instrumental stigma in high risk professional situations was reduced in the intervention group (p<0.001) - intervention participants had a greater reduction in blame, symbolic stigma, and transmission misconceptions postintervention than control participants.  **Alternative results:**  **Unintended consequences:** NA |
| Uys, 2009 [64]  **Country:** Lesotho, Malawi, South Africa, Swaziland, Tanzania  **Study design:** Pre-post  **Domain:** Healthcare | **Participant details:** People living with HIV and AIDS and nurses; the nurses were identified by the nurse managers of their facilities as being interested in or involved with HIV/AIDS care, and people living with HIV/AIDS were identified by support groups active in the area  **Sample size:** N=175  134 nurses, 41 PLWHA  **Study objective:** To explore the results of an HIV stigma intervention involving both information giving and empowerment in five African health care settings  **Implementer:** Academic researchers  **Cooccurring stigmas:** NA  **Definitions:**  Stigma = undesirable or discrediting attribute that an individual possesses, which affects the person's status in society  Internal stigma = thoughts and behavior stemming form the person's own negative perceptions about themselves based on their HIV statu | **Intervention**  The intervention combined (1) sharing information (e.g., information on impact of stigma), (2) increasing contact with the affected group, and (3) improving coping through empowerment, and consisted of bringing together a team of approximately 10 nurses and 10 people living with HIV or AIDS in each setting and facilitating a process in which they planned and implemented a stigma reduction intervention  **Duration (months)**: Ranged from 35-49 hours by site  **Comparator**  Baseline  **Length of follow up:** 1 months | **Stigma results:** Nurses who participated in intervention teams showed no change in stigma (HIV=AIDS Stigma Instrument–Nurse; p=0.812) while those living with HIV or AIDS showed a significant reduction in overall perceived stigma (HIV=AIDS Stigma Instrument–PLWA; p=0.003) and workplace stigma (p=0.015).  **Alternative results:**  **Unintended consequences:** NA |
| Varas-Diaz, 2013 [66]  Marzan-Rodriguez, 2015 [102]  **Country:** Puerto Rico  **Study design:** RCT  **Domain:** Healthcare | **Participant details:** Second year medical students recruited from the four largest medical schools in Puerto Rico  **Sample size:** N=507  **Study objective:** To assess the efficacy of an intervention to reduce HIV stigma attitudes among medical students  **Implementer:** Academic researchers  **Cooccurring stigmas:** NA  **Definitions:**  Structural stigma = societal-level conditions, cultural norms, and institutional policies that constrain the opportunities, resources, and wellbeing of the stigmatized | **Intervention**  The SPACES workshops were facilitated by health professionals that had experience with HIV-related patients; sessions targeted the sources and functions of HIV stigma, issues that can increase the severity of consequences, and instrumental and symbolic stigmas that manifest for HIV and included HIV knowledge, role of negative emotions in interactions, and behavioral skills  **Duration (months)**: 3 workshops, 3 hours each of individual sessions; 0.03 months  **Comparator**  Control group received only basic HIV epidemiology workshop information as a time and attention-matched experience and not the SPACES intervention workshop  **Length of follow up:** 12 months | **Stigma results:** Mean levels of HIV stigma (Spanish HIV Stigma Scale) were significantly lower in the intervention group compared to control (Cohen's d -0.30).  **Alternative results:**  **Unintended consequences:** NA |
| Wu, 2008 [70]  **Country:** China  **Study design:** RCT  **Domain:** Healthcare | **Participant details:** Service providers consisting of doctors, nurses, and lab technicians from 4 county hospitals  **Sample size:** N=138  **Study objective:** To reduce stigma and increase level of comfort working with people living with HIV/AIDS for service providers  **Implementer:** Academic researchers and NGO (Yunnan Province of Drug Addiction)  **Cooccurring stigmas:** NA  **Definitions:**  NA | **Intervention**  After a small group discussion on discriminatory language, attitudes, and behaviors in a medical setting, participants engaged in two rounds of a role-play session on “Discrimination among us,” which emphasizes the prevalence of discrimination in society and how it can make everyone a potential victim; all facilitators participated in training prior to the intervention  **Duration (months)**: 4 hour session  **Comparator**  No intervention  **Length of follow up:** 6 months | **Stigma results:** Service providers in the intervention condition were significantly more likely to report better protection of patients’ confidentiality (p=0.0018) and right to HIV testing (P<0.0001), lower levels of negative feelings toward people living with HIV/AIDS (p=0.0395), and more accurate understanding and practice of universal precautions (e.g., wearing gloves, p=0.0013).  **Alternative results:**  **Unintended consequences:** NA |
| Yiu, 2010 [72]  **Country:** China  **Study design:** RCT  **Domain:** Healthcare | **Participant details:** Nursing students enrolled in the bachelor’s program in nursing in Hong Kong  **Sample size:** N=89  **Study objective:** To compare the effectiveness and sustainability of an AIDS knowledge-only program with a combined program of knowledge and contact with people having HIV/AIDS  **Implementer:** NGO (AIDS Concern) and Academic researchers  **Cooccurring stigmas:** NA  **Definitions:**  NA | **Intervention**  A 50-min standardized lecture and a question-and-answer session plus in-vivo contact with PHA (50-min sharing session given by two male PHA)  **Duration (months)**: 50 minutes; 0.03 months  **Comparator**  A 50-min standardized lecture and a question-and-answer session alone  **Length of follow up:** 1.5 months | **Stigma results:** There was significantly less stigmatizing attitudes at post-test than pre-test in both the knowledge group (p=0.03) and the knowledge-contact group (p< 0.001) but no significant difference was found at 6-months follow up, suggesting a fading out of the effect.  **Alternative results:**  **Unintended consequences:** |

Notes: CI confidence interval, duration: intervention duration; length of follow up: outcome assessment from baseline; NA not available, not applicable

Main analyses are shown in the manuscript. Note that only a very small number of studies included a total facility approach (n=3) or an awareness campaign (n=2). We did not detect a systematic effect of the intervention category that was statistically significant (p=0.4246) but the number of studies represented in each of the individual categories was small. While interventions to reduce stigma and discrimination in healthcare settings showed less heterogeneity across studies than internalized stigma interventions, there was no linear effect of the context on the effect estimate (p=0.7506).

## Geographical Distribution of Interventions to Address Stigma and Discrimination in Healthcare Settings


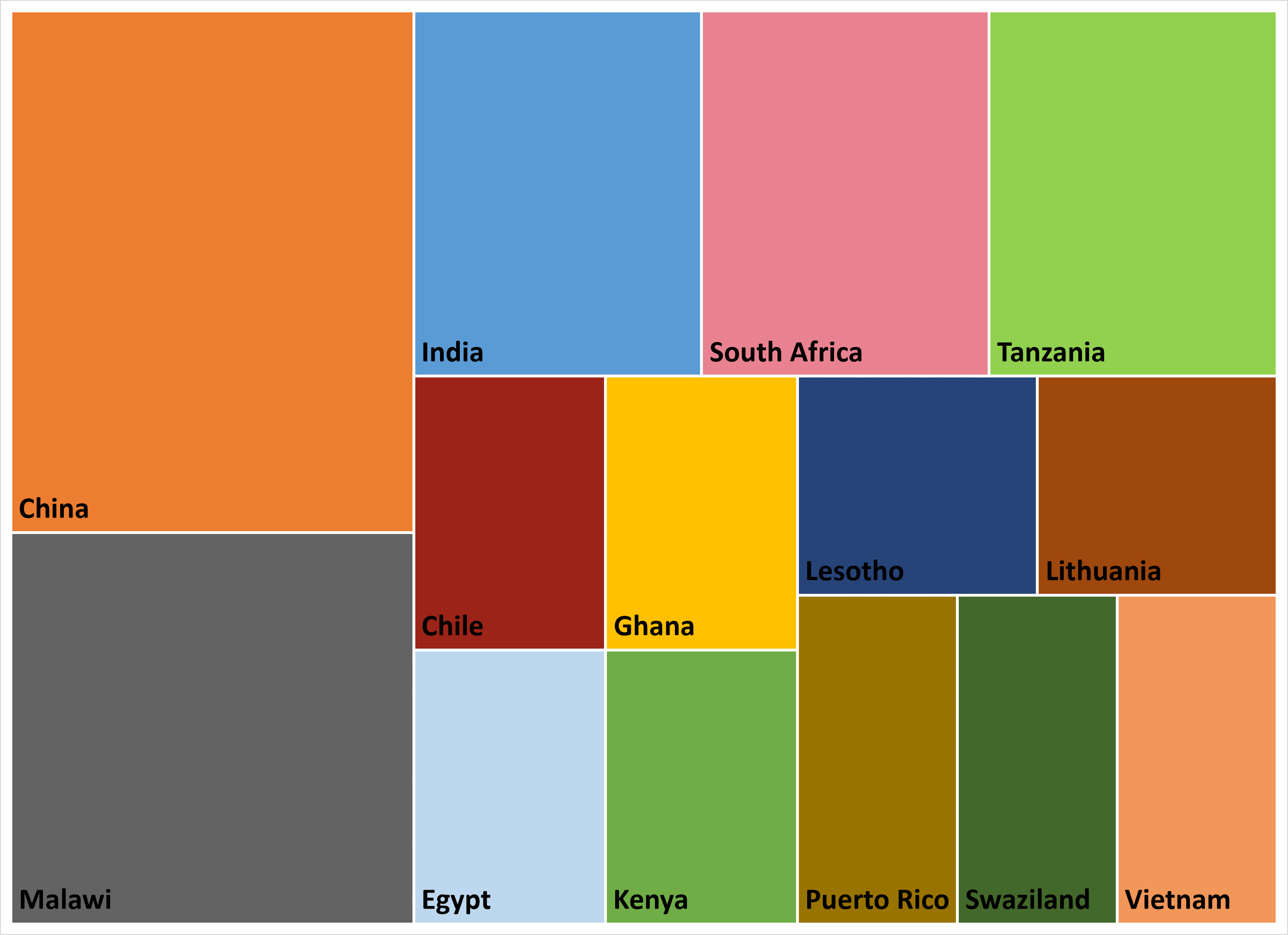


## Evidence table: Law and Policy Interventions

| **Author, year**  **Country**  **Study design**  **Domain** | **Population**  **Cooccurring stigmas**  **Context/setting**  **Study objective** | **Intervention**  **Comparator**  **Duration**  **Definitions** | **Stigma results**  **Other effects**  **Unintended consequences** |
| --- | --- | --- | --- |
| Adia, 2020 [3]  **Country:** Philippines  **Study design:** Post only  **Domain:** Law | **Population**  HIV-positive men who have sex with men in Manila, Philippines  **Cooccurring stigmas:** Sexual orientation  **Context / setting:** Community  **Study objective:** To examine how MSM living with HIV perceive HIV-relevant legal protections in the Philippines and how these protections influenced their lives | **Type:** Legal change  **Intervention**  The Philippines has adopted a targeted approach to eliminating discrimination based on HIV status using HIV-specific legislation  **Comparator**  NA  **Duration:** NA  **Definitions**  NA | **Stigma results:** Overall, participants viewed legal protections as both empowering and protective. Empowerment was achieved as protections helped participants manage internalized stigma, feel as if they had a weapon to fight discrimination, and perceive a more equitable climate around HIV within broader society. While participants felt as though the law sent positive signals that the government wanted to protect PLHIV, they doubted the actual legal process of bringing suits, leading to harms. Overall, this research presents ways in which legal protections can considered in interventions to empower PLHIV and also identifies opportunities to improve research and advocacy in settings with similarly protective laws.  **Other effects:**  **Unintended consequences:** NA |
| Brito, 2015 [10]  **Country:** USA  **Study design:** Post only  **Domain:** Law | **Population**  People living with HIV in Florida  **Cooccurring stigmas:** NA  **Context / setting:** Other : School  **Study objective:** To urge states to revisit criminal statutes in order to ensure that they are consistent with current knowledge of HIV transmission | **Type:** Court decision  **Intervention**  Ray vs School District of DeSoto County in Florida in 1987  **Comparator**  NA  **Duration:** Since 1998  **Definitions**  NA | **Stigma results:** NA  **Other effects:** A preliminary injunction was issued against the DeSoto County School Board. The concern and fear of the parents is not substantiated by science and the harm that the Ray children could pose to the public was merely theoretical, and the irreparable injury that would be done to the children by depriving them of their education outweighed any theoretical harm that they could possibly pose to society by attending school. The case ended in the order to reintegrate children with HIV/AIDS into the school district.  **Unintended consequences:** NA |
| Gallently, 2012 [15]  **Country:** USA  **Study design:** Post only  **Domain:** Law | **Population**  HIV-positive Michigan residents  **Cooccurring stigmas:** NA  **Context / setting:** Other : Michigan's HIV exposure law  **Study objective:** To discuss an empirical study of Michigan's HIV exposure law | **Type:** Discriminatory law or policy  **Intervention**  Michigan's criminal HIV exposure law: The law requires persons living with HIV to disclose their HIV-positive serostatus to prospective sex partners prior to engaging in sexual penetration with that partner. Sexual penetration is defined very broadly to include not only anal, vaginal, and oral intercourse but also “...any other intrusion, however slight, of any part of a person's body or of any object into the genital or anal openings of another person's body...”  **Comparator**  NA  **Duration:** NA  **Definitions**  HIV-related stigma = internalization of negative character traits associated with being a PLWH | **Stigma results:** NA  **Other effects:** A survey indicated that persons unaware of Michigan's law perceived more HIV-related stigma, perhaps because social isolation led both to their ignorance of the law and their internalization of stigmatizing attitudes. Comparisons of aware and unaware participants’ scores on the internalized HIV-related stigma scale revealed a significant difference. There was an inverse relationship between internalized stigma and awareness of the state HIV exposure law.  **Unintended consequences:** Concerns about the harmful effects of HIV exposure laws on people living with HIV were not supported by the data. Law-aware HIV-positive participants did not perceive greater societal hostility toward people living with HIV than those who were unaware of the law. Participants’ comfort with serostatus disclosure also did not differ significantly between persons who were and were not aware of the law, suggesting that the law does not have an inadvertent deterrent effect on seropositive status disclosure. |
| Gruskin, 2013 [17]  **Country:** Kenya  **Study design:** Controlled trial  **Domain:** Law | **Population**  Open Society Foundations supported legal integration activities at the following sites: the AMPATH facility, where Legal Aid Centre of Eldoret (LACE) operates, in Eldoret; Kenyatta National Hospital’s Gender-based Violence Recovery Centre, which hosts the Coalition on Violence against Women (COVAW) legal integration program; and Christian Health Association of Kenya (CHAK) facilities in Mombasa and Naivasha for clients, most of whom are people living with HIV  **Cooccurring stigmas:** NA  **Context / setting:** Healthcare  **Study objective:** To evaluate the impact of legal empowerment programs on health and human rights for those living with HIV | **Type:** Legal empowerment  **Intervention**  Interventions include training of clients and health providers on legal and human rights issues, provisioning of legal representation in formal judicial processes, helping clients resolve cases through informal conflict resolution mechanisms for legal aid services, and referring clients to legal and non-legal services to increase legal empowerment  **Comparator**  Control groups of untrained clients  **Duration:** NA  **Definitions**  NA | **Stigma results:** NA  **Other effects:** Notable increase in practical knowledge and awareness about how to access legal aid and claim rights and an enhanced ability to communicate with healthcare providers and to improve their access to healthcare and justice. Providers became more adept at identifying human rights violations and other legal difficulties, enabling them to give clients basic information about their rights, refer them to legal aid and assist them in accessing needed support.  **Unintended consequences:** NA |
| Lane, 2011 [21]  **Country:** Russia  **Study design:** Post only  **Domain:** Law | **Population**  People living with HIV in Russia  **Cooccurring stigmas:** NA  **Context / setting:** Healthcare  **Study objective:** To discuss the European Court of Human Rights decision that found Russia's public health concerns do not outweigh the right to privacy of individuals living with HIV | **Type:** Court decision  **Intervention**  The European Council of Human Rights found that HIV/AIDS-positive status is not proper grounds for discrimination in residency permit applications as health status should be considered an area of prohibited discrimination (refusing residency to anyone who cannot prove their HIV-negative status thus pulling families apart), in Kifutir v Russia  **Comparator**  NA  **Duration:** NA  **Definitions**  NA | **Stigma results:** With the support of the United Nations and Council of Europe, the ECHR found that health status should be considered an area of prohibited discrimination under article 14, and thus, this case could properly be tried under article 14, in consideration with article 8. The ECHR found that “such an indiscriminate refusal of residence permit, without an individualized judicial evaluation and solely based on a health condition, cannot be considered compatible with the protection against discrimination enshrined in Article 14 of the Convention.” Thus, taking into account the fact that Kiyutin belonged to a vulnerable group whose status is protected under article 14, and that Russia was not able to provide reasonable justifications for its discrimination, the ECHR found that Kiyutin was a victim of discrimination in violation of article 14 in combination with article 8 of the Convention. The landmark decision essentially inserts health status, specifically a person's HIV/AIDS status, into areas of discrimination regulated by article 14 of the Convention.  **Other effects:**  **Unintended consequences:** NA |
| Mahajan, 2016 [26]  **Country:** USA  **Study design:** Cohort study  **Domain:** Law | **Population**  Patients attending two publicly funded safety-net ambulatory clinics in Los Angeles County  **Cooccurring stigmas:** NA  **Context / setting:** Healthcare  **Study objective:** To determine whether opt-out HIV screening as compared to opt-in HIV screening is associated with increased uptake of HIV testing | **Type:** Policy change  **Intervention**  The physician-initiated opt-out model was implemented at the two clinics during the first 2 months; in the subsequent 4 months, the nurse- initiated screening models were implemented; for the physician initiated opt-out model, the physician offered the test during the regular course of his or her face-to-face encounter with the patient; physicians underwent two 60 min training sessions on the basic procedures of initiating opt-out HIV screening; for both the opt-in and opt-out models, the screening initiator (nurse or physician) explained that HIV testing is being offered to all patients in the clinic at no charge and described the process of rapid testing with an oral swab  **Comparator**  In one of the clinics, the nurse-initiated opt-out model was implemented for 2 months, followed by 2 months of the nurse initiated opt-in model; in the other clinic, the nurse- initiated opt-in model was implemented first, followed by the nurse-initiated opt-out model; a licensed vocational nurse served as the ‘nurse’ initiator for the screening models that were nurse initiated (i.e., nurse initiated opt-in and nurse initiated opt-out); nurses underwent two 90 min training sessions on the basic procedures of offering both opt-in and opt-out HIV screening; in the opt-in model only, the nurse initiator read a verbal consent checklist together with the patient prior to offering the patient an HIV test  **Duration:** 6 months  **Definitions** | **Stigma results:** There were no significant interaction effects in logistic regression models to test for potential effect modification of HIV screening model on relationship between stigma and HIV test acceptance (blame/isolation sub-scale, p=0.30; contagion sub-scale, p=0.93; abandonment sub-scale, p=0.10).  **Other effects:** NA  **Unintended consequences:** NA |
| Miller, 2021 [31]  **Country:** Jamaica, Zimbabwe, Dominican Republic  **Study design:** Post only  **Domain:** Law | **Population**  Members at MPact or one of the 7 lead partner organizations, were sexual and gender minority community members, staff representatives of civil society organizations collaborating with one of the lead partner organizations, health care professionals, journalists and government officials  **Cooccurring stigmas:** Sexual orientation, gender identify  **Context / setting:** Healthcare,Community,Other : policies, laws  **Study objective:** To identify outcomes from community-led advocacy initiatives targeting barriers to HIV care for gay and bisexual men and transgender women in African and Caribbean countries | **Type:** Legal advocacy  **Intervention**  Coordinated set of community-led advocacy initiatives targeting structural changes that might eliminate barriers to HIV care for gay and bisexual men and transgender women in five African and two Caribbean countries. For example, in Jamaica, transgender people were coached through the process of participating in the Universal Periodic Review on Jamaica’s human rights record and on shadow reporting, In Zimbabwe, nursing staff underwent training to become Project ACT advocacy champions.  **Comparator**  NA  **Duration:** 20 months  **Definitions**  NA | **Stigma results:** Results included the first transgender-specific submission from Jamaica to a treaty body in the United Nation and a community-based organization that serves vulnerable youth drafted its first-ever policy on transgender youth. In Zimbabwe, sensitization training became a standard part of the curriculum in one of the largest nursing training programs in the western region of the country and a clinic established a policy that all new employees regardless of their position must undergo sensitization training. In the Dominican Republic, the Ministry of Health signed a two-party Memorandum of Understanding that guarantees health care workers’ attendance at sensitization training and establishes a national training calendar. Overall, skills gained through the process of learning to document stigma and discrimination, intentional efforts to mobilize the community, and efforts to reduce self-stigma were the primary contributors to the vast majority (81%) of advocacy capacity outcomes.  **Other effects:**  **Unintended consequences:** Person who agreed to be the subject of a small media video produced for the project; its unexpected popularity had made it an unwelcome intrusion in her life. Health care  providers were stigmatized by colleagues for attending sensitization trainings in Cameroon. Radio hosts reported they were shunned and stigmatized by colleagues for putting sexual and gender minority people on air; Individuals who participated in self-stigma reduction activities that were part of mobilizing service demand experienced a loss of personal resources after seeking to claim their rights or honor their identities. In one case, an individual was jailed after calling the police to report he was being harassed over his sexuality. In the other, a minor transgender youth suffered the loss of family support and access to basic resources, including housing, food, and tuition. |
| Onyemelukwe, 2017 [39]  **Country:** Nigeria  **Study design:** Post only  **Domain:** Law | **Population**  Nigerians living with HIV/AIDS  **Cooccurring stigmas:** Sexual orientation, drug use, sex work  **Context / setting:**  **Study objective:** To analyze the law in Nigeria and to argue that the time has come to amend the constitution to include health status upon which one cannot be unduly discriminated against | **Type:** Legal change  **Intervention**  Federal HIV/AIDS Anti-Discrimination Act 2014 (purpose of the Act is to protect the rights of persons living with HIV through the elimination of all forms of discrimination, creating a supportive environment to encourage such persons working as long as they are medically fit to do so, creating a safe working environment for all), a judicial decisions in 2012 (Ahamefule v. Imperial Medical Centre and DrAlex Molokwu) and 2016 (XXX v. Smridu Nigeria Limited) addressed discrimination on the basis of HIV status  **Comparator**  NA  **Duration:** since 2012  **Definitions**  Stigma = reflects the prejudice, tension, conflict, silence, subterfuge and hypocrisy; it is an attitude of ‘us’ versus ‘them’, which makes an ‘other’ out of the other person | **Stigma results:** Developments suggest a positive shift of the law to full protection of the rights of persons living with HIV/AIDS in Nigeria, particularly in the area of employment. The court ruled that Mrs Ahamefule posed no material risk and therefore her termination was done in bad faith and actuated by malice. The National Industrial Court found that routine, mandatory testing was in contravention of the Lagos State Law and that the termination was done on the discriminatory grounds of HIV status.  **Other effects:**  **Unintended consequences:** NA |
| Schwartz, 2015 [47]  **Country:** Nigeria  **Study design:** Pre-post  **Domain:** Law | **Population**  16 years or older, assigned male sex at birth, had engaged in insertive or receptive anal sex with a man in the past 12 months, spoke English or Hausa, presented with a valid study coupon, and were willing to be followed up for 18 months, including regular HIV testing and clinical monitoring  **Cooccurring stigmas:** Sexual orientation (men who have sex with men)  **Context / setting:** Healthcare,Community  **Study objective:** To assess the immediate effect of the prohibition act on stigma, discrimination, and engagement in HIV prevention and treatment services | **Type:** Discriminatory law or policy  **Intervention**  Participants began enrolling in the cohort in March 2013, about nine months before the Same-Sex Marriage Prohibition Act was publicly announced as law on Jan 13, 2014; the cohort remained open as of June 2015. Participants completed a baseline behavioral questionnaire (visit 0) prior to the effective date of the Act, returned within two weeks to complete HIV counseling and testing, testing for sexually transmitted infections, and clinical and laboratory examinations (visit 1). Quarterly visits were scheduled after the baseline visit and included a condensed behavioral questionnaire, HIV counseling and testing or HIV treatment monitoring and STI testing. All men having sex with men living with HIV were offered antiretroviral treatment independent of CD4 cell count.  **Comparator**  Pre-law data  **Duration:** Since 2014  **Definitions**  NA | **Stigma results:**  **Other effects:** Incidence of fear of seeking health care was 2.57 (CI 1.29, 5.10) times greater in the postlaw period than in the prelaw period.  **Unintended consequences:** NA |
| Sears, 2013 [48]  **Country:** USA  **Study design:** Post only  **Domain:** Law | **Population**  People living with HIV receiving dental care in Los Angeles, CA  **Cooccurring stigmas:** NA  **Context / setting:** Healthcare  **Study objective:** To discuss the established view in the medical community that dentist can effectively and safely treat patients with HIV | **Type:** Court decision  **Intervention**  In a 1998 case of Bragdon v. Abbott, the U.S. Supreme Court determined that people living with HIV/AIDS, even if asymptomatic, were covered by the Americans with Disabilities Act. The case involved a dentist who refused to provide services at his office to a woman living with HIV.  **Comparator**  NA  **Duration:** Since 1998  **Definitions**  NA | **Stigma results:** 90% of all dental offices contacted in Los Angeles County (551) responded that they would treat people living with HIV/AIDS; 5% had an unlawful blanket policy of refusing dental services to people living with HIV/AIDS, an additional 5% indicated they would treat people living with HIV/AIDS differently than other patients in ways that could potentially violate state and federal antidiscrimination laws.  **Other effects:**  **Unintended consequences:** NA |
| Sourcebook, 2020 [54]  **Country:** USA  **Study design:** Post only  **Domain:** Law | **Population**  People living with HIV  **Cooccurring stigmas:** NA  **Context / setting:** Community,Other : Policy  **Study objective:** To include laws and illustrative cases in each state, U.S. territory, and federal law on the treatment of people living with HIV in the criminal legal system | **Type:** Court decision  **Intervention**  In People v. Plunkett, the Court of Appeals of New York shifted away from considering bodily fluid of PLWHA a deadly weapon. In 2014 State v. Hogg the Supreme Court of Tennessee clarified the meaning of significant risk. The defendant had been convicted of seven counts of criminal exposure to HIV, amongst other crimes, and challenged the sufficiency of the evidence supporting his conviction on appeal. The court rejected the defendant’s argument that substantial risk required risks so great they are almost certain to materialize if nothing is done. Instead, the court held that significant risk requires a possibility of HIV transmission that is more definite than a faint, speculative risk, as shown by expert medical proof. The court explained that this determination requires a fact-specific inquiry, including an assessment of both the severity of consequences and the likelihood that HIV will be transmitted. Applying this standard and relying on testimony by an infectious disease physician, the court found that there was insufficient evidence to support the defendant’s conviction for three of the seven counts of criminal exposure to HIV.  **Comparator**  NA  **Duration:** Since 2014  **Definitions**  NA | **Stigma results:** PLWHA may also be charged with general criminal laws, such as aggravated assault, or maybe criminally liable for a range of acts. PLWHA convicted of the criminal exposure statute may be required to register as sex offenders. PLWHA may be prosecuted for specific intent to transmit disease. PLWHA may receive increased sentences or aggravated assault charges for sex crimes. PLWHA may face criminal penalties for sharing needles. PLWHA are prohibited from donating blood or blood products, organs, human tissue, semen, ova, or breast milk except under specific circumstances. PLWHA can be prosecuted for failing to disclose their HIV status to sexual partners. PLWHA may face enhanced criminal penalties for prostitution and solicitation of prostitution. PLWHA is also prosecuted under the Felonious Assault Statute for spitting or biting.  **Other effects:**  **Unintended consequences:** NA |
| UNAIDS, 2021 [60]  **Country:** India, Philippines  **Study design:** Post only  **Domain:** Law | **Population**  People living with HIV and key populations in Asia and the Pacific  **Cooccurring stigmas:** Sexual orientation, gender identity, sex work, drug use  **Context / setting:** Community  **Study objective:** To provide a better understanding of laws and policies that hinder an effective HIV response in Asia and the Pacific essential to ending AIDS by 2030 | **Type:** Legal change  **Intervention**  In 2015, 193 United Nations Member States agreed on the 2030 Agenda for Sustainable Development; this included a goal of ending the AIDS epidemic by 2030 and leaving no one behind through a multi-sectoral, rights-based, people-centered approach that addresses the determinants of health.  **Comparator**  NA  **Duration:** Agreement from 2015, developments followed through 2019  **Definitions**  NA | **Stigma results:** NA  **Other effects:** India has passed a comprehensive national HIV law in 2017. The Philippines revised its national HIV law in 2018. Both countries introduced comprehensive new HIV legislation confirming a human rights-based approach to managing their HIV epidemics. These laws address multiple aspects of the national HIV response in a single law, including legal frameworks for prevention, testing, treatment, discrimination, legal redress mechanisms, national leadership and coordination mechanisms. They provide useful models for other countries in the region. In both cases, these achievements occurred after extensive stakeholder consultations and community debates about the appropriate legal response to managing a public health challenge affecting some of the most stigmatized and marginalized populations of these countries.  **Unintended consequences:** NA |
| UNDP, 2016 [61]  **Country:** Jamaica  **Study design:** Post only  **Domain:** Law | **Population**  NA  **Cooccurring stigmas:** NA  **Context / setting:** Healthcare,Workplace,Community  **Study objective:** To determine Jamaica's own contribution towards strengthening national capacity to ensure citizen’s security and human rights for reform of HIV related law and policy | **Type:** Legal advocacy  **Intervention**  UNDP worked with the National HIV Programme to support legal reform initiatives by providing support to advocacy on legislative and policy changes in 2014  **Comparator**  NA  **Duration:** Implemented in 2014, evaluated in 2016  **Definitions**  NA | **Stigma results:**  **Other effects:** The extent to which the project has effected any sustained legal reform is too early to determine but it has successfully created a springboard from which advocacy for policy and legislative changes can be increased. For example, the project established a plan of action for amendment of HIV-related legislation and policy.  **Unintended consequences:** NA |
| UNDP, 2019 [62]  Ferguson, 2018 [103]; UNDP, 2018 [104]; UNDP, 2015 [105]  **Country:** Democratic Republic of the Congo. Ghana, Sierra Leone  **Study design:** Post only  **Domain:** Law | **Population**  LGBT people and women and girls affected by HIV and AIDS in Sub-Saharan Africa  **Cooccurring stigmas:** Sex worker, sexual orientation, gender identity, incarceration  **Context / setting:** Healthcare,Community  **Study objective:** To strengthen national and regional legal environments relating to HIV and sexual and reproductive health and rights | **Type:** Legal advocacy  **Intervention**  The United Nations Development Programme (UNDP) launched the project Strengthening Regional and National Legislative Environments for HIV/SRHR to Support the Enjoyment of Human Rights of LGBT People and Women and Girls in Sub-Saharan Africa – Phase II which followed a project Strengthening Regional and National Legislative Environments to Support the Human Rights of LGBT People and Women and Girls affected by HIV and AIDS in Sub-Saharan Africa  **Comparator**  NA  **Duration:** 2013-2019, evaluated in 2019  **Definitions**  NA | **Stigma results:** NA  **Other effects:** Ghana AIDS Commission Act will support the operationalization of its anti-stigma provisions. In Sierra Leone, the National AIDS Commission Act 2011 was reviewed and protections against stigma improved. Other improvements in laws include provisions in Ghana's AIDS Control Bill to protect and promote the rights of people living with HIV; an increase in the minimum age of marriage to 19 (from 14 for girls and 16 for boys) in the Democratic Republic of the Congo.  **Unintended consequences:** Started difficult conversations around adolescent sexual and reproductive health and abortion as an unintended outcome. |
| UNDP, 2021 [63]  **Country:** Global report (India, the Philippines)  **Study design:** Post only  **Domain:** Law | **Population**  NA  **Cooccurring stigmas:** NA  **Context / setting:** Community,Other : Judicial system  **Study objective:** To examine the relationship between HIV and the law, and includes recommendations covering the breadth of the HIV response | **Type:** Legal change  **Intervention**  In 2010, UNDP, on behalf of the Joint UN Programme on HIV/AIDS (UNAIDS), convened the Global Commission on HIV and the Law (the Global Commission) to examine legal and human rights issues through an HIV lens, increase awareness among key constituencies on issues of rights and law, and engage with and strengthen civil society  **Comparator**  NA  **Duration:** Established in 2010, evaluated in 2021  **Definitions**  NA | **Stigma results:** The fact that follow-up activities to the Global Commission have been carried out across so many different countries around the world is a testament to the relevance of its work everywhere. Regional-level work has been followed by national level follow-up tailored to the context. For example, in 2018, the Supreme Court India overturned Section 377 of the Indian Penal Code which stated that “carnal intercourse against the order of nature” was a criminal offense punishable by life imprisonment.” The court used evidence presented by the Commission report. In 2015, the Botswana Court of Appeal upheld a ruling that foreign prisoners should receive free HIV treatment. Presiding were judges who had attended  the Regional Judges’ Forum.  **Other effects:**  **Unintended consequences:** NA |

Notes: CI confidence interval, duration: intervention duration; length of follow up: outcome assessment from baseline; NA not available, not applicable

**Geographical Distribution of Identified Interventions to Address Stigma and Discrimination in Law and Policy**


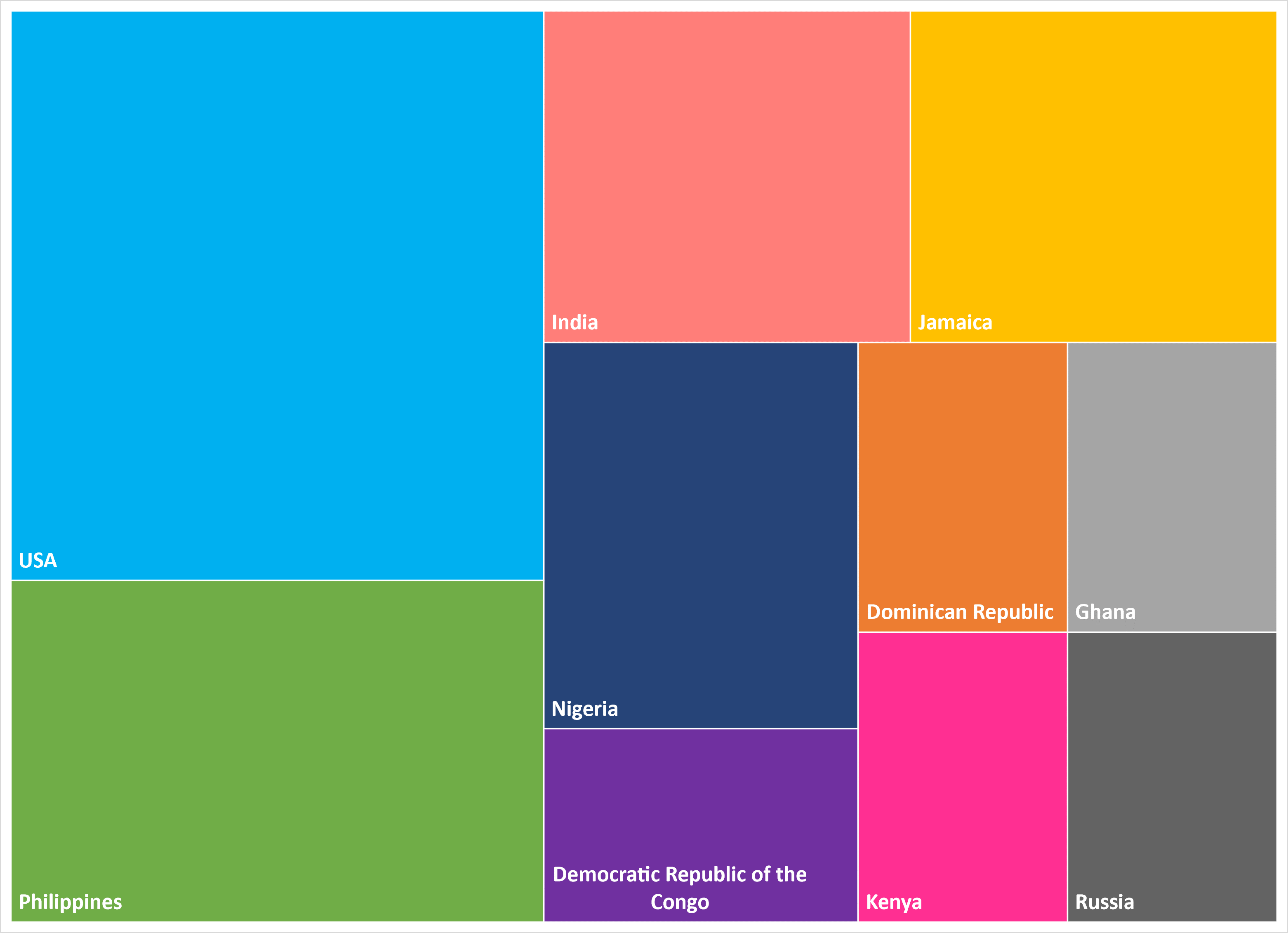


## Qualitative Comparative Analysis Results

### Component Analysis of Interventions to Address Internalized Stigma or Stigma and Discrimination in Healthcare Settings

| **Study ID** | **Context** | **Intervention type** | **Rights-holder** | **Duty-bearer** | **Conceptual framework** | **Internalized focus** | **Support person** | **Cooccurring stigma^a^** | **Community** | **PLHIV** | **Education** | **Counseling** | **Awareness** | **Referral** | **HIV specialist** | **Total facility approach** |
| --- | --- | --- | --- | --- | --- | --- | --- | --- | --- | --- | --- | --- | --- | --- | --- | --- |
| Barroso, 2014 [31] | I | Individual level only | √ |  |  |  |  |  |  |  | √ |  |  |  |  |  |
| Batchelder, 2020 [32] | I | Individual level only | √ |  | √ |  |  | √ |  |  |  | √ |  |  |  |  |
| Bauermeister, 2019 [33] | I | Individual and relational levels | √ |  |  | √ |  | √ |  |  |  | √ |  |  |  |  |
| Bhatta, 2017 [34] | I | Individual and structural levels | √ |  | √ |  |  |  |  |  | √ | √ |  |  | √ |  |
| Bogart, 2021 [35] | I | Individual level only | √ |  |  |  |  | √ | √ |  |  | √ |  |  |  |  |
| Chidrawi, 2016 [37] | I | Individual and structural levels | √ |  | √ |  |  |  | √ |  | √ |  |  |  |  |  |
| Denison, 2020 [38] | I | Individual and relational levels | √ |  |  |  | √ |  |  |  |  | √ |  |  |  |  |
| Denison, 2021 [39] | I | Individual and relational levels | √ |  |  |  | √ |  |  |  | √ | √ |  |  | √ |  |
| France, 2019 [40] | I | Individual and relational levels | √ |  | √ | √ | √ |  | √ |  |  | √ |  |  |  |  |
| Go, 2015 [42] | I | Individual and relational levels | √ |  |  | √ |  | √ | √ |  | √ | √ |  |  |  |  |
| Harper, 2014 [44] | I | Individual and relational levels | √ |  |  |  |  |  | √ |  | √ | √ |  | √ |  |  |
| Hickey, 2021 [45] | I | Individual and relational levels | √ |  |  |  | √ |  | √ |  | √ | √ |  |  | √ |  |
| Lifson, 2017 [49] | I | Individual and structural levels | √ |  |  |  | √ |  | √ | √ | √ | √ |  | √ | √ |  |
| Lowther, 2018 [51] | I | Individual level only | √ |  |  |  |  |  |  |  |  | √ |  | √ |  |  |
| Maluccia, 2017 [54] | I | Individual and structural levels | √ |  |  |  |  |  |  |  |  | √ |  |  | √ |  |
| Masquillier, 2015 [55] | I | Individual level only | √ |  |  |  | √ |  |  |  |  | √ |  |  |  |  |
| Nestadt, 2019 [59] | I | Individual and relational levels | √ |  |  |  | √ |  | √ |  |  | √ |  |  | √ |  |
| Nyamathi, 2013 [61] | I | Individual and structural levels | √ |  |  | √ |  |  |  |  | √ | √ |  |  |  |  |
| Peltzer, 2018 [66] | I | Individual and relational levels | √ |  |  |  |  |  |  |  | √ | √ |  |  |  |  |
| Petersen, 2014 [67] | I | Individual level only | √ |  |  | √ |  |  |  |  |  | √ |  |  |  |  |
| Prinsloo, 2017 [68] | I | Individual and relational levels | √ |  |  | √ |  |  | √ | √ | √ | √ |  |  |  |  |
| Rao, 2012 [70] | I | Individual level only | √ |  |  | √ |  | √ | √ | √ | √ | √ |  |  |  |  |
| Rao, 2018 [71] | I | Individual level only | √ |  |  | √ | √ | √ | √ | √ |  | √ |  |  |  |  |
| Rongkavilit, 2015 [72] | I | Individual level only | √ |  |  |  |  | √ | √ |  |  | √ |  |  | √ |  |
| Shamsaei, 2020 [75] | I | Individual level only | √ |  |  |  |  |  |  |  | √ |  |  |  |  |  |
| Singh, 2020 [76] | I | Individual and structural levels | √ |  |  | √ | √ | √ | √ |  |  | √ |  | √ | √ |  |
| Skinta, 2015 [78] | I | Individual level only | √ |  |  |  |  | √ |  |  |  | √ |  |  | √ |  |
| Step, 2020 [81] | I | Individual level only | √ |  |  |  | √ |  |  |  | √ | √ |  |  |  |  |
| Tsai, 2013 [83] | I | Structural level only | √ |  |  |  |  |  |  |  |  | √ |  | √ | √ |  |
| Tsai, 2017 [84] | I | Structural level only | √ |  |  |  |  |  |  |  |  |  |  |  |  |  |
| Tshabalala, 2011 [85] | I | Individual level only | √ |  |  | √ |  |  |  |  |  | √ |  |  |  |  |
| Van Tam, 2012 [91] | I | Individual level only | √ |  |  |  | √ |  |  |  |  |  |  |  |  |  |
| Wagner, 2012 [93] | I | Structural level only | √ |  |  |  |  |  |  |  |  | √ |  | √ | √ |  |
| Watt, 2011 [94] | I | Individual and relational levels | √ |  | √ |  | √ |  | √ | √ | √ | √ |  |  | √ |  |
| Watt, 2020 [95] | I | Individual and relational levels | √ |  | √ | √ | √ |  |  |  |  | √ |  |  | √ |  |
| Yigit, 2020 [97] | I | Individual level only | √ |  | √ |  |  |  |  |  | √ | √ |  |  | √ |  |
| Arora, 2014 [30] | HC | Information-Skills |  | √ |  |  |  |  |  |  | √ |  |  |  |  |  |
| Kaponda, 2009 [46] | HC | Information-Skills |  | √ |  |  | √ |  |  |  | √ |  |  |  |  |  |
| Li, 2013 [48] | HC | Information-Skills-Structural |  | √ |  |  |  |  |  |  | √ |  |  |  |  |  |
| Lohiniva, 2016 [50] | HC | Information-Skills-Structural |  | √ |  |  |  |  | √ |  | √ |  |  |  |  |  |
| Mak, 2015 [53] | HC | Information-Skills |  | √ |  |  |  | √ | √ |  | √ |  |  |  |  |  |
| Mbela, 2011 [56] | HC | Information-Skills |  | √ |  |  | √ |  | √ |  | √ | √ |  |  |  |  |
| Mockiene, 2011 [58] | HC | Information |  | √ |  |  |  |  | √ |  | √ |  |  |  |  |  |
| Norr, 2012 [60] | HC | Information |  | √ |  |  | √ |  |  |  | √ |  |  | √ |  |  |
| Nyblade, 2018 [63] | HC | Information-Skills-Structural |  | √ | √ |  |  |  | √ |  | √ | √ |  |  |  | √ |
| Nyblade, 2020 [62] | HC | Information-Skills-Structural |  | √ |  |  |  | √ | √ |  | √ |  | √ |  |  | √ |
| Odeny, 2013 [64] | HC | Information-Structural |  | √ |  |  |  |  | √ |  | √ |  |  | √ | √ |  |
| Pulerwitz, 2015 [69] | HC | Information-Skills-Structural-Contact |  | √ | √ |  |  |  |  | √ | √ |  |  |  |  | √ |
| Siraprapasiri, 2020 [77] | HC | Information-Skills-Structural |  | √ | √ |  |  | √ | √ |  | √ |  |  | √ |  |  |
| Sommerland, 2020 [79] | HC | Information-Structural |  | √ | √ |  |  | √ |  |  | √ |  | √ |  |  |  |
| Srinivasan, 2021 [80] | HC | Information-Skills-Contact |  | √ | √ |  |  |  |  | √ | √ | √ |  |  |  |  |
| Uys, 2009[90] | HC | Information-Skills-Contact | √ | √ |  |  |  |  | √ | √ | √ | √ |  |  |  |  |
| Varas-Diaz, 2013 [92] | HC | Information |  | √ |  |  |  |  |  |  | √ |  |  |  |  |  |
| Wu, 2008 [96] | HC | Information-Skills-Contact |  | √ |  |  |  |  |  | √ |  |  |  |  |  |  |
| Yiu, 2010 [98] | HC | Information-Contact |  | √ |  |  |  |  |  | √ | √ |  |  |  |  |  |
| **Grand Total** | **55** | **N/A** | **36** | **19** | **2** | **11** | **16** | **13** | **23** | **10** | **34** | **35** | **2** | **9** | **15** | **3** |

Notes: I Internalized stigma, HC stigma and discrimination in healthcare, N/A not applicable; for definitions of components see text

a Co-occurring stigma: This indicates studies in which the study population may have experienced other types of stigma beyond HIV-related stigma e.g. on the basis of race/ethnicity or sexual orientation, irrespective of whether the intervention itself was designed to address this additional stigma.

We reviewed different possible component combinations predicting success. To address the limited diversity in our sample, we selected a small number of configurations, informed by the boosted regression analysis and conceptual considerations.

### Internalized Stigma and Healthcare Dataset combined

**Truth Table (Community, Education, Counseling, Support person, HIV specialist; combined analysis)**

     Community Education Counseling HIV_specialist Support_person   OUT    n  incl  PRI  
 1       0         0         0            0              0           1     2  1.000 1.000
 2       0         0         0            0              1           0     1  0.000 0.000
 5       0         0         1            0              0           0     3  0.333 0.333
 6       0         0         1            0              1           1     2  1.000 1.000
 7       0         0         1            1              0           1     4  0.750 0.750
 8       0         0         1            1              1           1     1  1.000 1.000
 9       0         1         0            0              0           1     7  0.714 0.714
10       0         1         0            0              1           1     2  1.000 1.000
13       0         1         1            0              0           1     3  1.000 1.000
14       0         1         1            0              1           1     1  1.000 1.000
15       0         1         1            1              0           1     2  1.000 1.000
16       0         1         1            1              1           1     1  1.000 1.000
21       1         0         1            0              0           1     1  1.000 1.000
22       1         0         1            0              1           1     2  0.500 0.500
23       1         0         1            1              0           0     1  0.000 0.000
24       1         0         1            1              1           0     1  0.000 0.000
25       1         1         0            0              0           1     5  1.000 1.000
27       1         1         0            1              0           1     1  1.000 1.000
29       1         1         1            0              0           0     6  0.333 0.333
30       1         1         1            0              1           1     1  1.000 1.000
32       1         1         1            1              1           1     3  1.000 1.000

Note: Condition selected based on boosted regression. Of 32 possible combinations, no cases exist for 21 condition combinations. OUT successful (1) vs not), n number of studies, incl consistency, PRI proportional reduction in inconsistency.

**Coverage after Minimization (Community, Education, Counseling, Support person, HIV specialist; combined analysis)**

| inclS PRI covS covU  ------------------------------------------------------------------------------------------------   1. ~Community*Education*Counseling 1.000 1.000 0.189 0.135   2 ~Community*Counseling*Support_person 1.000 1.000 0.135 0.081  3 Education*Counseling*Support_person 1.000 1.000 0.162 0.108  4 ~Community*Education*~HIV_specialist*Support_person 1.000 1.000 0.081 0.054  5 Community*Education*~Counseling*~Support_person 1.000 1.000 0.162 0.162  6 ~Community*~Education*~Counseling*~HIV_specialist*~Support_person 1.000 1.000 0.054 0.054  7 Community*~Education*Counseling*~HIV_specialist*~Support_person 1.000 1.000 0.027 0.027  ------------------------------------------------------------------------------------------------  M1 1.000 1.000 0.676 |
| --- |

Note: inclS consistency of sufficiency, PRI proportional reduction in inconsistency, covS raw coverage for sufficiency, covU unique coverage.

### Internalized Stigma Dataset

**Truth Table (Community, Counseling, Support person, PLWH; internalized stigma dataset)**

Support_person Community Counseling PLWH OUT n incl PRI

1 0 0 0 0 1 3 1.000 1.000

3 0 0 1 0 0 11 0.727 0.727

5 0 1 0 0 1 1 1.000 1.000

7 0 1 1 0 0 4 0.500 0.500

8 0 1 1 1 0 2 0.000 0.000

9 1 0 0 0 0 1 0.000 0.000

11 1 0 1 0 1 5 1.000 1.000

15 1 1 1 0 0 3 0.667 0.667

16 1 1 1 1 0 3 0.667 0.667

Note: Condition selected based on conceptual considerations. Of 16 possible combinations, 9 have been tested empirically. PLWH person openly living with HIV. OUT successful (1) vs not), n number of studies, incl consistency, PRI proportional reduction in inconsistency.

**Coverage after Minimization (Community, Counseling, Support person, PLWH; internalized stigma dataset)**

inclS PRI covS covU

-------------------------------------------------------------------------

1 ~Support_person*~Counseling*~PLWH 1.000 1.000 0.174 0.174

2 Support_person*~Community*Counseling*~PLWH 1.000 1.000 0.217 0.217

-------------------------------------------------------------------------

M1 1.000 1.000 0.391

Note: inclS consistency of sufficiency, PRI proportional reduction in inconsistency, covS raw coverage for sufficiency, covU unique coverage.

**Truth Table (Community, Education, Counseling, Support person, PLWH; internalized stigma dataset)**

Community Education Counseling Support_person PLWH OUT n incl PRI

1 0 0 0 0 0 1 1 1.000 1.000

3 0 0 0 1 0 0 1 0.000 0.000

5 0 0 1 0 0 0 7 0.571 0.571

7 0 0 1 1 0 1 3 1.000 1.000

9 0 1 0 0 0 1 2 1.000 1.000

13 0 1 1 0 0 1 4 1.000 1.000

15 0 1 1 1 0 1 2 1.000 1.000

21 1 0 1 0 0 0 2 0.500 0.500

23 1 0 1 1 0 0 2 0.500 0.500

24 1 0 1 1 1 0 1 0.000 0.000

25 1 1 0 0 0 1 1 1.000 1.000

29 1 1 1 0 0 0 2 0.500 0.500

30 1 1 1 0 1 0 2 0.000 0.000

31 1 1 1 1 0 1 1 1.000 1.000

32 1 1 1 1 1 1 2 1.000 1.000

Note: Condition selected based on conceptual and empirical considerations. Of 32 possible combinations, 15 have been tested empirically. PLWH person openly living with HIV. OUT successful (1) vs not), n number of studies, incl consistency, PRI proportional reduction in inconsistency.

**Coverage after Minimization (Community, Education, Counseling, Support person, PLWH; internalized stigma dataset)**

inclS PRI covS covU (M1) (M2)

------------------------------------------------------------------------------------------

1 ~Community*~Counseling*~Support_person*~PLWH 1.000 1.000 0.130 0.043 0.043 0.043

2 ~Community*Counseling*Support_person*~PLWH 1.000 1.000 0.217 0.130 0.130 0.217

3 Community*Education*Counseling*Support_person 1.000 1.000 0.130 0.130 0.130 0.130

4 Education*~Counseling*~Support_person*~PLWH 1.000 1.000 0.130 0.043 0.043 0.043

------------------------------------------------------------------------------------------

5 ~Community*Education*Counseling*~PLWH 1.000 1.000 0.261 0.000 0.174

6 ~Community*Education*~Support_person*~PLWH 1.000 1.000 0.261 0.000 0.174

------------------------------------------------------------------------------------------

M1 1.000 1.000 0.696

M2 1.000 1.000 0.696

Note: inclS consistency of sufficiency, PRI proportional reduction in inconsistency, covS raw coverage for sufficiency, covU unique coverage.

### Stigma and Discrimination in Healthcare Settings Dataset

**Truth Table (Community, Education, Total facility approach; healthcare dataset)**

Education Community Total_facility_approach OUT n incl PRI

1 0 0 0 1 1 1.000 1.000

5 1 0 0 0 7 0.714 0.714

6 1 0 1 1 1 1.000 1.000

7 1 1 0 1 6 0.833 0.833

8 1 1 1 1 2 1.000 1.000

Note: Condition selected based on conceptual considerations. Of 8 possible combinations, 5 have been tested empirically. OUT successful (1) vs not), n number of studies, incl consistency, PRI proportional reduction in inconsistency.

**Coverage after Minimization (Community, Education, Total facility approach; healthcare dataset)**

inclS PRI covS covU

-----------------------------------------------------------------------------

1 Education*Community 0.875 0.875 0.500 0.357

2 Education*Total_facility_approach 1.000 1.000 0.214 0.071

3 ~Education*~Community*~Total_facility_approach 1.000 1.000 0.071 0.071

-----------------------------------------------------------------------------

M1 0.900 0.900 0.643

Note: inclS consistency of sufficiency, PRI proportional reduction in inconsistency, covS raw coverage for sufficiency, covU unique coverage.

# REFERENCES

1. Global Network Of People Living with HIV (GNP+). **The People Living with HIV Stigma Index**. 2008. Available at: https://www.stigmaindex.org/ [Accessed 22 February 2021]

2. Thomas J, O'Mara-Eves A, Brunton G. **Using qualitative comparative analysis (QCA) in systematic reviews of complex interventions: a worked example**. *Syst Rev* 2014; 3:67.

3. Adia AC, Restar AJ, Lee CJ, Payawal MP, Quilantang MI, Nazareno J, et al. **Sword and Shield: Perceptions of law in empowering and protecting HIV-positive men who have sex with men in Manila, Philippines**. *Global Public Health* 2020; 15(1):52-63.

4. Arora SS, Jyoti S, Chakravarty S. **Effectiveness of an empowering programme on student nurses' understanding and beliefs about HIV/AIDS**. *International Journal of Nursing Education* 2014; 6(1):88-92.

5. Barroso J, Relf MV, Williams MS, Arscott J, Moore ED, Caiola C, et al. **A randomized controlled trial of the efficacy of a stigma reduction intervention for HIV-infected women in the Deep South**. *AIDS Patient Care STDS* 2014; 28(9):489-498.

6. Batchelder AW, Moskowitz JT, Jain J, Cohn M, Earle MA, Carrico AW. **A Novel Technology-Enhanced Internalized Stigma and Shame Intervention for HIV-Positive Persons With Substance Use Disorders**. *Cogn Behav Pract* 2020; 27(1):55-69.

7. Bauermeister JA, Muessig KE, LeGrand S, Flores DD, Choi SK, Dong W, et al. **HIV and Sexuality Stigma Reduction Through Engagement in Online Forums: Results from the HealthMPowerment Intervention**. *AIDS Behav* 2019; 23(3):742-752.

8. Bhatta DN, Liabsuetrakul T. **Efficacy of a Social Self-Value Empowerment Intervention to Improve Quality of Life of HIV Infected People Receiving Antiretroviral Treatment in Nepal: A Randomized Controlled Trial**. *AIDS and behavior* 2016.

9. Bogart LM, Barreras JL, Gonzalez A, Klein DJ, Marsh T, Agniel D, et al. **Pilot Randomized Controlled Trial of an Intervention to Improve Coping with Intersectional Stigma and Medication Adherence Among HIV-Positive Latinx Sexual Minority Men**. *AIDS Behav* 2021; 25(6):1647-1660.

10. Brito M. **On an Alternative to a Punitive Standard in Response to a More Modern Understanding of the HIV/AIDS Epidemic in Florida Notes and Comments**. *Nova L Rev* 2015; 40:285-348.

11. Chidrawi HC, Greeff M, Temane QM, al. e. **HIV stigma experiences and stigmatisation before and after an intervention**. *Health SA Gesondheid* 2016; 21:196-205.

12. Denison JA, Burke VM, Miti S, Nonyane BAS, Frimpong C, Merrill KG, et al. **Project YES! Youth Engaging for Success: A randomized controlled trial assessing the impact of a clinic-based peer mentoring program on viral suppression, adherence and internalized stigma among HIV-positive youth (15-24 years) in Ndola, Zambia**. *PLoS One* 2020; 15(4):e0230703.

13. Denison JA, Packer C, Nyambe N, Hershow RB, Caldas S, Miti S, et al. **Family Connections randomized controlled trial: assessing the feasibility and acceptability of an intervention with adolescents living with HIV and their caregivers in Ndola, Zambia**. *AIDS Care* 2021:1-10.

14. France NF, Macdonald SHF, Conroy RR, Chiroro P, Cheallaigh DN, Nyamucheta M, et al. **‘We are the change’—An innovative community-based response to address self-stigma: A pilot study focusing on people living with HIV in Zimbabwe**. *PLoS ONE* 2019; 14(2):24.

15. Galletly CL, Pinkerton SD, DiFranceisco W. **A quantitative study of Michigan's criminal HIV exposure law**. *AIDS Care* 2012; 24(2):174-179.

16. Go VF, Frangakis C, Minh NL, Latkin C, Ha TV, Mo TT, et al. **Efficacy of a Multi-level Intervention to Reduce Injecting and Sexual Risk Behaviors among HIV-Infected People Who Inject Drugs in Vietnam: A Four-Arm Randomized Controlled Trial**. *PLoS One* 2015; 10(5):e0125909.

17. Gruskin S, Safreed-Harmon K, Ezer T, Gathumbi A, Cohen J, Kameri-Mbote P. **Access to justice: evaluating law, health and human rights programmes in Kenya**. *J Int AIDS Soc* 2013; 16(3 Suppl 2):18726.

18. Harper GW, Lemos D, Hosek SG. **Stigma reduction in adolescents and young adults newly diagnosed with HIV: findings from the Project ACCEPT intervention**. *AIDS Patient Care STDS* 2014; 28(10):543-554.

19. Hickey MD, Ouma GB, Mattah B, Pederson B, DesLauriers NR, Mohamed P, et al. **The Kanyakla study: Randomized controlled trial of a microclinic social network intervention for promoting engagement and retention in HIV care in rural western Kenya**. *PLoS One* 2021; 16(9):e0255945.

20. Kaponda CP, Jere DL, Chimango JL, Chimwaza AF, Crittenden KS, Kachingwe SI, et al. **Impacts of a peer-group intervention on HIV-related knowledge, attitudes, and personal behaviors for urban hospital workers in Malawi**. *J Assoc Nurses AIDS Care* 2009; 20(3):230-242.

21. Lane HH. **Kiyutin v. Russia: The European Court of Human Rights Acknowledges the Need for Protection of a Class of Individuals with HIV/AIDS Recent Developments**. *Tul J Int'l & Comp L* 2011; 20:505-518.

22. Li L, Wu Z, Liang LJ, Lin C, Guan J, Jia M, et al. **Reducing HIV-related stigma in health care settings: a randomized controlled trial in China**. *Am J Public Health* 2013; 103(2):286-292.

23. Lifson AR, Workneh S, Hailemichael A, Demisse W, Slater L, Shenie T. **Implementation of a Peer HIV Community Support Worker Program in Rural Ethiopia to Promote Retention in Care**. *J Int Assoc Provid AIDS Care* 2017; 16(1):75-80.

24. Lohiniva AL, Benkirane M, Numair T, Mahdy A, Saleh H, Zahran A, et al. **HIV stigma intervention in a low-HIV prevalence setting: a pilot study in an Egyptian healthcare facility**. *AIDS Care* 2016; 28(5):644-652.

25. Lowther K, Harding R, Simms V, Gikaara N, Ahmed A, Ali Z, et al. **Effect of participation in a randomised controlled trial of an integrated palliative care intervention on HIV-associated stigma**. *AIDS Care* 2018; 30(9):1180-1188.

26. Mahajan AP, Kinsler JJ, Cunningham WE, James S, Makam L, Manchanda R, et al. **Does the Centers for Disease Control and Prevention's Recommendation of Opt-Out HIV Screening Impact the Effect of Stigma on HIV Test Acceptance?** *AIDS Behav* 2016; 20(1):107-114.

27. Mak WW, Cheng SS, Law RW, Cheng WW, Chan F. **Reducing HIV-related stigma among health-care professionals: a game-based experiential approach**. *AIDS Care* 2015; 27(7):855-859.

28. Maluccio JA, Wu F, Rokon RB, Rawat R, Kadiyala S. **Assessing the Impact of Food Assistance on Stigma Among People Living with HIV in Uganda Using the HIV/AIDS Stigma Instrument-PLWA (HASI-P)**. *AIDS Behav* 2017; 21(3):766-782.

29. Masquillier C, Wouters E, Mortelmans D, le Roux Booysen F. **The impact of community support initiatives on the stigma experienced by people living with HIV/AIDS in South Africa**. *AIDS Behav* 2015; 19(2):214-226.

30. Mbeba MM, Kaponda CP, Jere DL, Kachingwe SI, Crittenden KS, McCreary LL, et al. **Peer group intervention reduces personal HIV risk for Malawian health workers**. *J Nurs Scholarsh* 2011; 43(1):72-81.

31. Miller RL, Rutledge J, Ayala G. **Breaking Down Barriers to HIV Care for Gay and Bisexual Men and Transgender Women: The Advocacy and Other Community Tactics (ACT) Project**. *AIDS Behav* 2021; 25(8):2551-2567.

32. Mockiene V, Suominen T, Valimaki M, Razbadauskas A, Martinkenas A, Caplinskas S. **The impact of an education intervention to change nurses' HIV-related knowledge and attitudes in Lithuania: a randomized controlled trial**. *J Assoc Nurses AIDS Care* 2011; 22(2):140-149.

33. Nestadt DF, Saisaengjan C, McKay MM, Bunupuradah T, Pardo G, Lakhonpon S, et al. **CHAMP+ Thailand: Pilot randomized control trial of a family-based psychosocial intervention for perinatally HIV-infected early adolescents**. *AIDS Patient Care and STDs* 2019; 33(5):227-236.

34. Norr KF, Ferrer L, Cianelli R, Crittenden KS, Irarrazabal L, Cabieses B, et al. **Peer group intervention for HIV prevention among health workers in Chile**. *J Assoc Nurses AIDS Care* 2012; 23(1):73-86.

35. Nyamathi A, Ekstrand M, Salem BE, Sinha S, Ganguly KK, Leake B. **Impact of Asha intervention on stigma among rural Indian women with AIDS**. *West J Nurs Res* 2013; 35(7):867-883.

36. Nyblade L, Mbuya-Brown RJ, Sabasaba AN, Ezekiel M, Kiwia P, al. e. **Understanding and Responding to Stigma and Discrimination in Health Facilities in Tanzania**. In. Washington, DC: Palladium, Health Policy Plus; 2018.

37. Nyblade L, Addo NA, Atuahene K, Alsoufi N, Gyamera E, Jacinthe S, et al. **Results from a difference-in-differences evaluation of health facility HIV and key population stigma-reduction interventions in Ghana**. *J Int AIDS Soc* 2020; 23(4):e25483.

38. Odeny TA, Penner J, Lewis-Kulzer J, Leslie HH, Shade SB, Adero W, et al. **Integration of HIV Care with Primary Health Care Services: Effect on Patient Satisfaction and Stigma in Rural Kenya**. *AIDS Res Treat* 2013; 2013:485715.

39. Onyemelukwe C. **Discrimination on the Basis of HIV Status: An Analysis of Recent Developments in Nigerian Law and Jurisprudence**. *Int'l J Discrimination & L* 2017; 17:160-179.

40. Peltzer K, Babayigit S, Rodriguez VJ, Jean J, Sifunda S, Jones DL. **Effect of a multicomponent behavioural PMTCT cluster randomised controlled trial on HIV stigma reduction among perinatal HIV positive women in Mpumalanga province, South Africa**. *Sahara j* 2018; 15(1):80-88.

41. Petersen I, Hanass Hancock J, Bhana A, Govender K. **A group-based counselling intervention for depression comorbid with HIV/AIDS using a task shifting approach in South Africa: a randomized controlled pilot study**. *J Affect Disord* 2014; 158:78-84.

42. Prinsloo CD, Greeff M, Kruger A, Khumalo IP. **HIV stigma experiences and stigmatisation before and after a HIV stigma-reduction community "hub" intervention**. *Afr J AIDS Res* 2017; 16(3):203-213.

43. Pulerwitz J, Oanh KT, Akinwolemiwa D, Ashburn K, Nyblade L. **Improving hospital-based quality of care by reducing HIV-related stigma: evaluation results from Vietnam**. *AIDS Behav* 2015; 19(2):246-256.

44. Rao D, Desmond M, Andrasik M, Rasberry T, Lambert N, Cohn SE, et al. **Feasibility, acceptability, and preliminary efficacy of the unity workshop: an internalized stigma reduction intervention for African American women living with HIV**. *AIDS Patient Care STDS* 2012; 26(10):614-620.

45. Rao D, Kemp CG, Huh D, Nevin PE, Turan J, Cohn SE, et al. **Stigma Reduction Among African American Women With HIV: UNITY Health Study**. *J Acquir Immune Defic Syndr* 2018; 78(3):269-275.

46. Rongkavilit C, Wang B, Naar-King S, Bunupuradah T, Parsons JT, Panthong A, et al. **Motivational interviewing targeting risky sex in HIV-positive young Thai men who have sex with men**. *Arch Sex Behav* 2015; 44(2):329-340.

47. Schwartz SR, Nowak RG, Orazulike I, Keshinro B, Ake J, Kennedy S, et al. **The immediate eff ect of the Same-Sex Marriage Prohibition Act on stigma, discrimination, and engagement on HIV prevention and treatment services in men who have sex with men in Nigeria: analysis of prospective data from the TRUST cohort**. *Lancet HIV* 2015; 2(7):e299-306.

48. Sears B, Cooper C, Younai FS, Donohoe T. **HIV Discrimination in Dental Care: Results of a Testing Study in Los Angeles County**. 2012.

49. Shamsaei F, Tahour N, Sadeghian E. **Effect of Stress Management Training on Stigma and Social Phobia in HIV-Positive Women**. *J Int Assoc Provid AIDS Care* 2020; 19:2325958220918953.

50. Singh RJ, Sarna A, Schensul JJ, Mahapatra B, Ha T, Schensul SL. **A multilevel intervention to reduce stigma among alcohol consuming men living with HIV receiving antiretroviral therapy: findings from a randomized control trial in India**. *Aids* 2020; 34 Suppl 1:S83-s92.

51. Siraprapasiri T, Srithanaviboonchai K, Chantcharas P, Suwanphatthana N, Ongwandee S, Khemngern P, et al. **Integration and scale-up of efforts to measure and reduce HIV-related stigma: the experience of Thailand**. *Aids* 2020; 34 Suppl 1:S103-s114.

52. Skinta MD, Lezama M, Wells G, Dilley JW. **Acceptance and compassion-based group therapy to reduce HIV stigma**. *Cogn Behav Pract* 2015; 22(4):481-490.

53. Sommerland N, Masquillier C, Rau A, Engelbrecht M, Kigozi G, Pliakas T, et al. **Reducing HIV- and TB-Stigma among healthcare co-workers in South Africa: Results of a cluster randomised trial**. *Soc Sci Med* 2020; 266:113450.

54. The Center for HIV Law & Policy. **HIV Criminalization in the United States: A Sourcebook on State and Federal HIV Criminal Law and Practice**. In. Third ed; 2020.

55. Srinivasan K, Heylen E, Raj T, Nyblade L, Devadass D, Pereira M, et al. **Reduction in Stigma Drivers Partially Mediates the Effect of a Stigma Reduction Intervention Among Nursing Students in India: The DriSti Cluster Randomized Controlled Trial**. *J Acquir Immune Defic Syndr* 2021; 86(2):182-190.

56. Step MM, Knight K, McMillen Smith J, Lewis SA, Russell TJ, Avery AK. **Positive Peers Mobile Application Reduces Stigma Perception Among Young People Living With HIV**. *Health Promot Pract* 2020; 21(5):744-754.

57. Tsai AC, Bangsberg DR, Bwana M, Haberer JE, Frongillo EA, Muzoora C, et al. **How does antiretroviral treatment attenuate the stigma of HIV? Evidence from a cohort study in rural Uganda**. *AIDS Behav* 2013; 17(8):2725-2731.

58. Tsai AC, Hatcher AM, Bukusi EA, Weke E, Lemus Hufstedler L, Dworkin SL, et al. **A Livelihood Intervention to Reduce the Stigma of HIV in Rural Kenya: Longitudinal Qualitative Study**. *AIDS Behav* 2017; 21(1):248-260.

59. Tshabalala J, Visser M. **Developing a cognitive behavioural therapy model to assist women to deal with HIV and stigma**. *S Afr J Psychol* 2011; 41(1):17-28.

60. UNAIDS. **Legal and policy trends impacting people living with HIV and key populations in Asia and the Pacific 2014–2019**. 2021.Available at <https://www.unaids.org/sites/default/files/media_asset/legal-and-policy-trends-asia-pacific_en.pdf> [Accessed 20 April 2021]

61. UNDP. **Evaluation of the "BUILDING CAPACITY FOR REFORM OF HIV RELATED LAW AND POLICY IN JAMAICA" Project"** 2016. Available at: https://erc.undp.org/evaluation/evaluations/detail/7308 [Accessed 20 April 2021]

62. UNDP. **End-term review HIV Law SIDA Project (Africa).** 2019. Available at: https://erc.undp.org/evaluation/evaluations/detail/9379 [Accessed 20 April 2021]

63. UNDP. **Global Project for the Evaluation of the Global Commission on HIV and the Law.** 2021. Available at: https://erc.undp.org/evaluation/evaluations/detail/9380# [Accessed 20 April 2021]

64. Uys L, Chirwa M, Kohi T, Greeff M, Naidoo J, Makoae L, et al. **Evaluation of a health setting-based stigma intervention in five African countries**. *AIDS Patient Care STDS* 2009; 23(12):1059-1066.

65. Van Tam V, Larsson M, Pharris A, Diedrichs B, Nguyen HP, Nguyen CT, et al. **Peer support and improved quality of life among persons living with HIV on antiretroviral treatment: a randomised controlled trial from north-eastern Vietnam**. *Health Qual Life Outcomes* 2012; 10:53.

66. Varas-Díaz N, Neilands TB, Cintrón-Bou F, Marzán-Rodríguez M, Santos-Figueroa A, Santiago-Negrón S, et al. **Testing the efficacy of an HIV stigma reduction intervention with medical students in Puerto Rico: the SPACES project**. *J Int AIDS Soc* 2013; 16(3 Suppl 2):18670.

67. Wagner GJ, Ghosh-Dastidar B, Garnett J, al. e. **Impact of HIV antiretroviral therapy on depression and mental health among clients with HIV in Uganda**. *Psychosom Med* 2012; 74(9):883-890.

68. Watt MH, Aronin EH, Maman S, Thielman N, Laiser J, John M. **Acceptability of a group intervention for initiates of antiretroviral therapy in Tanzania**. *Glob Public Health* 2011; 6(4):433-446.

69. Watt MH, Minja L, Knettel BA, Mwamba RN, Osaki H, Ngocho JS, et al. **Pilot Outcomes of Maisha: An HIV Stigma Reduction Intervention Developed for Antenatal Care in Tanzania**. *AIDS Behav* 2020; 25(4):1171-1184.

70. Wu S, Li L, Wu Z, Liang LJ, Cao H, Yan Z, et al. **A brief HIV stigma reduction intervention for service providers in China**. *AIDS Patient Care STDS* 2008; 22(6):513-520.

71. Yigit I, Modi RA, Weiser SD, Johnson MO, Mugavero MJ, Turan JM, et al. **Effects of an intervention on internalized HIV-related stigma for individuals newly entering HIV care**. *Aids* 2020; 34 Suppl 1(Suppl 1):S73-s82.

72. Yiu JW, Mak WW, Ho WS, Chui YY. **Effectiveness of a knowledge-contact program in improving nursing students' attitudes and emotional competence in serving people living with HIV/AIDS**. *Soc Sci Med* 2010; 71(1):38-44.

73. Relf MV, Silva SG, Williams MS, Moore E, Arscott J, Caiola C, et al. **Feasibility of using an iPod touch device and acceptability of a stigma reduction intervention with HIV-infected women in the Deep South**. *AIDS Behav* 2015; 19(10):1896-1904.

74. Relf MV, Williams M, Barroso J. **Voices of Women Facing HIV-Related Stigma in the Deep South**. *J Psychosoc Nurs Ment Health Serv* 2015; 53(12):38-47.

75. Hightow-Weidman L, Legrand S, Simmons R, Egger J, Choi SK, Muessig KE. **healthMpowerment: Effects of a mobile phone-optimized, Internet-based intervention on condomless anal intercourse among young black men who have sex with men and transgender women**. In: *9th IAS Conference on HIV Science*. Paris, France; July 23-26, 2017.

76. Denison JA, Burke VM, Miti S, Nonyane BAS, Frimpong C, Merrill KG, et al. **Correction: Project YES! Youth Engaging for Success: A randomized controlled trial assessing the impact of a clinic-based peer mentoring program on viral suppression, adherence and internalized stigma among HIV-positive youth (15-24 years) in Ndola, Zambia**. *PLoS One* 2020; 15(4):e0232488.

77. Hosek SG, Lemos D, Harper GW, Telander K. **Evaluating the acceptability and feasibility of Project ACCEPT: an intervention for youth newly diagnosed with HIV**. *AIDS Educ Prev* 2011; 23(2):128-144.

78. Lowther K, Selman L, Simms V, Gikaara N, Ahmed A, Ali Z, et al. **Nurse-led palliative care for HIV-positive patients taking antiretroviral therapy in Kenya: a randomised controlled trial**. *Lancet HIV* 2015; 2(8):e328-334.

79. Lowther K, Simms V, Selman L, Sherr L, Gwyther L, Kariuki H, et al. **Treatment outcomes in palliative care: the TOPCare study. A mixed methods phase III randomised controlled trial to assess the effectiveness of a nurse-led palliative care intervention for HIV positive patients on antiretroviral therapy**. *BMC Infect Dis* 2012; 12:288.

80. Maluccio JA, Palermo T, Kadiyala S, Rawat R. **Improving Health-Related Quality of Life among People Living with HIV: Results from an Impact Evaluation of a Food Assistance Program in Uganda**. *PLoS One* 2015; 10(8):e0135879.

81. Rawat R, Faust E, Maluccio JA, Kadiyala S. **The impact of a food assistance program on nutritional status, disease progression, and food security among people living with HIV in Uganda**. *J Acquir Immune Defic Syndr* 2014; 66(1):e15-22.

82. Wouters E, Masquillier C, Ponnet K, le Roux Booysen F. **A peer adherence support intervention to improve the antiretroviral treatment outcomes of HIV patients in South Africa: the moderating role of family dynamics**. *Soc Sci Med* 2014; 113:145-153.

83. Bhargava A, Booysen FLR, Walsh CM. **Health status, food insecurity, and time allocation patterns of patients with AIDS receiving antiretroviral treatment in South Africa**. *AIDS Care* 2018; 30(3):361-368.

84. Mellins CA, Nestadt D, Bhana A, Petersen I, Abrams EJ, Alicea S, et al. **Adapting Evidence-Based Interventions to Meet the Needs of Adolescents Growing Up with HIV in South Africa: The VUKA Case Example**. *Glob Soc Welf* 2014; 1(3):97-110.

85. Pardo G, Saisaengjan C, Gopalan P, Ananworanich J, Lakhonpon S, Nestadt DF, et al. **Cultural Adaptation of an Evidence-Informed Psychosocial Intervention to Address the Needs of PHIV+ Youth in Thailand**. *Glob Soc Welf* 2017; 4(4):209-218.

86. Prinsloo CD, Greeff M. **A Community "Hub" Network Intervention for HIV Stigma Reduction: A Case Study**. *J Assoc Nurses AIDS Care* 2016; 27(2):166-179.

87. Fabian K, Molina Y, Kemp CG, Nevin PE, McCoy K, Simoni JM, et al. **Internalized HIV-Related Stigma and Breast Health Beliefs Among African-American Women Receiving Care for HIV in the USA**. *J Racial Ethn Health Disparities* 2020; 7(1):45-51.

88. Cohen CR, Steinfeld RL, Weke E, Bukusi EA, Hatcher AM, Shiboski S, et al. **Shamba Maisha: Pilot agricultural intervention for food security and HIV health outcomes in Kenya: design, methods, baseline results and process evaluation of a cluster-randomized controlled trial**. *Springerplus* 2015; 4:122.

89. Weiser SD, Bukusi EA, Steinfeld RL, Frongillo EA, Weke E, Dworkin SL, et al. **Shamba Maisha: randomized controlled trial of an agricultural and finance intervention to improve HIV health outcomes**. *AIDS* 2015; 29(14):1889-1894.

90. Modi R, Amico KR, Knudson A, Westfall AO, Keruly J, Crane HM, et al. **Assessing effects of behavioral intervention on treatment outcomes among patients initiating HIV care: Rationale and design of iENGAGE intervention trial**. *Contemp Clin Trials* 2018; 69:48-54.

91. Li L, Liang LJ, Lin C, Wu Z. **Addressing HIV stigma in protected medical settings**. *AIDS Care* 2015; 27(12):1439-1442.

92. Li L, Lin C, Guan J, Wu Z. **Implementing a stigma reduction intervention in healthcare settings**. *J Int AIDS Soc* 2013; 16(3 Suppl 2):18710.

93. Li L, Liang LJ, Wu Z, Lin C, Guan J. **Assessing outcomes of a stigma-reduction intervention with venue-based analysis**. *Soc Psychiatry Psychiatr Epidemiol* 2014; 49(6):991-999.

94. Li L, Guan J, Liang LJ, Lin C, Wu Z. **Popular Opinion Leader intervention for HIV stigma reduction in health care settings**. *AIDS Educ Prev* 2013; 25(4):327-335.

95. Ferrer L, Cabieses B, Norr K, Cianelli R, Araya A, Irarrazabal L, et al. **[Effectiveness of an educational program about the Chilean AIDS law in primary care health workers]**. *Rev Med Chil* 2011; 139(5):625-632.

96. Nyblade L, Addo NA, Mingkwan P, Vormawor R, Stewart C, al. e. **Understanding and Responding to Stigma and Discrimination in Health Facilities in Ghana: Intervention Endline Report**. In. Washington, DC: Palladium, Health Policy Plus; 2018.

97. PEPFAR Solutions Platform. **Transforming Service Delivery for Improved Outcomes: A Total Facility Approach to Reducing Stigma and Discrimination**. January 16, 2020.

98. Khuat TO, Ashburn K, Pulerwitz J, Ogden J, Nyblade L. **Improving hospitalbased quality of care in Vietnam by reducing HIV-related stigma and discrimination, a Horizons final report**. In. Washington, DC: Population Council; 2008.

99. Ekstrand ML, Raj T, Heylen E, Nyblade L, Devdass D, Pereira M, et al. **Reducing HIV stigma among healthcare providers in India using a partly tablet-administered intervention: the DriSti trial**. *AIDS Care* 2020; 32(sup2):14-22.

100. Radhakrishna K, Dass D, Raj T, Rakesh D, Kishore R, Srinivasan K, et al. **Development of a Novel Tablet-based Approach to Reduce HIV Stigma among Healthcare Staff in India**. *Perspect Health Inf Manag* 2017; 14(Spring):1b.

101. Nyblade L, Srinivasan K, Mazur A, Raj T, Patil DS, Devadass D, et al. **HIV Stigma Reduction for Health Facility Staff: Development of a Blended- Learning Intervention**. *Front Public Health* 2018; 6:165.

102. Marzan-Rodriguez M, Varas-Diaz N, Neilands T. **Qualitative Contributions to a Randomized Controlled Trial Addressing HIV/AIDS-Stigma in Medical Students**. *Qual Rep* 2015; 20(12):2012-2024.

103. Ferguson L, Nicholson A, Henry I, Saha A, Sellers T, Gruskin S. **Assessing changes in HIV-related legal and policy environments: Lessons learned from a multi-country evaluation**. *PLoS One* 2018; 13(2):e0192765.

104. United Nations Development Programme. **Section 3: Terms of Reference (TOR): Ethiopia**. In. Ethioia: Signed by Mefin Geahun Haileyesus (Head, HIV, Health and Development Team, UNDP, RSCA [OIC]; 2018.

105. UNDP. **Mid-term Evaluation - UNDP RSC Africa: 'Strengthening Regional and National Legislative Environments to Support the Human Rights of LGBT People and Women and Girls affected by HIV and AIDS in Sub-Saharan Africa' (A SIDA Supported Project).** 2015.
